# Structural Analysis and Development of Notum Fragment Screening Hits

**DOI:** 10.1021/acschemneuro.2c00325

**Published:** 2022-06-22

**Authors:** Yuguang Zhao, William Mahy, Nicky J. Willis, Hannah L. Woodward, David Steadman, Elliott D. Bayle, Benjamin N. Atkinson, James Sipthorp, Luca Vecchia, Reinis R. Ruza, Karl Harlos, Fiona Jeganathan, Stefan Constantinou, Artur Costa, Svend Kjær, Magda Bictash, Patricia C. Salinas, Paul Whiting, Jean-Paul Vincent, Paul V. Fish, E. Yvonne Jones

**Affiliations:** † Division of Structural Biology, Wellcome Centre for Human Genetics, University of Oxford, The Henry Wellcome Building for Genomic Medicine, Roosevelt Drive, Oxford OX3 7BN, U.K.; ‡ Alzheimer’s Research UK UCL Drug Discovery Institute, University College London, The Cruciform Building, Gower Street, London WC1E 6BT, U.K.; § The Francis Crick Institute, 1 Midland Road, Kings Cross, London NW1 1AT, U.K.; ∥ Department of Cell and Developmental Biology, Laboratory for Molecular and Cellular Biology, University College London, London WC1E 6BT, U.K.

**Keywords:** Notum inhibitors, fragment screening, Diamond-SGC Poised Library (DSPL), hit-to-lead development, Wnt signaling

## Abstract

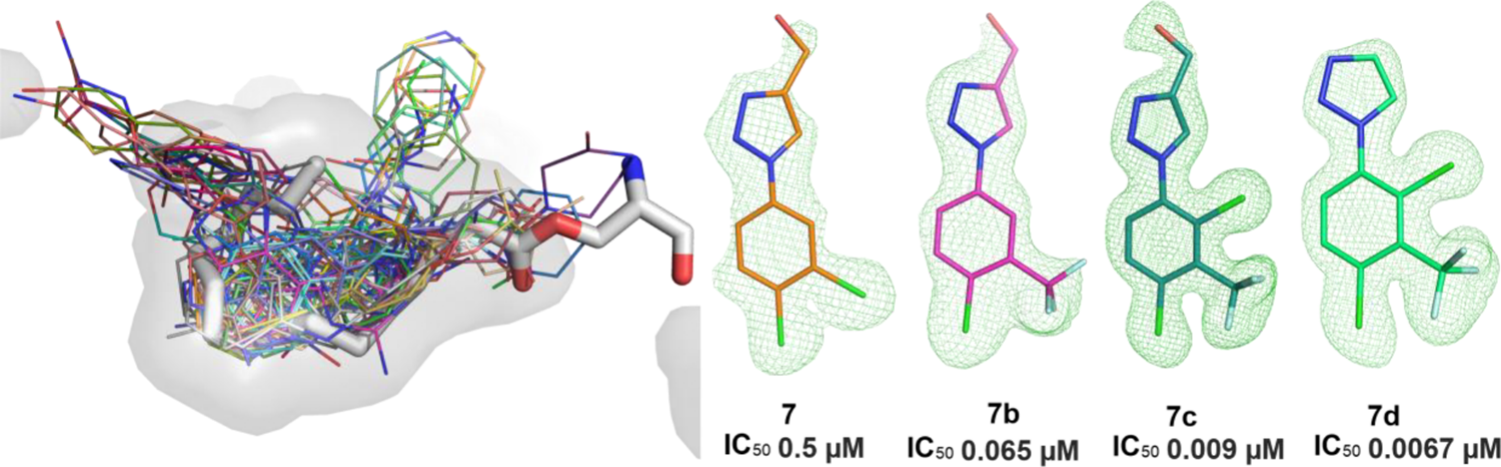

The Wnt signaling suppressor Notum is a promising target for osteoporosis, Alzheimer’s disease, and colorectal cancers. To develop novel Notum inhibitors, we used an X-ray crystallographic fragment screen with the Diamond-SGC Poised Library (DSPL) and identified 59 fragment hits from the analysis of 768 data sets. Fifty-eight of the hits were found bound at the enzyme catalytic pocket with potencies ranging from 0.5 to >1000 μM. Analysis of the fragments’ diverse binding modes, enzymatic inhibitory activities, and chemical properties led to the selection of six hits for optimization, and five of these resulted in improved Notum inhibitory potencies. One hit, 1**-**phenyl-1,2,3-triazole **7**, and its related cluster members, have shown promising lead-like properties. These became the focus of our fragment development activities, resulting in compound **7d** with IC_50_ 0.0067 μM. The large number of Notum fragment structures and their initial optimization provided an important basis for further Notum inhibitor development.

## Introduction

Wnt signaling pathways are fundamental for animal embryonic development, adult tissue homeostasis, and regeneration.^1^ The signaling is initiated when lipidated Wnt ligands engage both frizzled receptors and coreceptors such as LRP5/6 or ROR1/2.^1,2^ A conserved serine residue in Wnt proteins (such as serine-206 of Wnt7a) undergoes O-linked lipidation by the porcupine O-acyltransferase (PORCN).^3,4^ This post-translational modification is crucial for binding to frizzled receptors.^5,6^ Enzymatic removal of this covalently attached lipid disables Wnt function.^7^ The carboxyesterase Notum is the only known enzyme that can remove the Wnt palmiteoyl lipid.^7^ Thus, the inhibition of Notum activity could restore suppressed Wnt function, which may help in some Wnt hypoactive pathologies such as Alzheimer’s disease^8^ and osteoporosis.^9^ Surprisingly, in some Wnt hyperactive situations, Notum inhibition also shows promising beneficial effects. For example, in Wnt hyperactive adenomatous polyposis coli (APC)-mutant colorectal cancers, Notum protein is hugely overexpressed and Notum inhibition has been demonstrated to be able to limit cancer cell expansion and the formation of intestinal adenomas.^10^ These observations highlight the value of searching for powerful Notum inhibitory drugs. In addition, Notum may have other substrates, such as the serine O-linked ghrelin octanoyl lipid,^11^ meaning Notum inhibitors may have the potential to modulate activities other than Wnt signaling.

Small molecule inhibitors of Notum, such as LP-922056 and ABC99, have been identified that show promise in animal models to increase cortical bone thickness and strength,^12,13^ increase brain neuronal progenitor cell proliferation in the ventricular-subventricular zone (V-SVZ),^14^ and rejuvenate colon stem cells.^15^ These small molecule Notum inhibitors were discovered by high-throughput screening (HTS)^13^ or by an activity-based protein profiling (ABPP) with a library of activated carbamates.^16^

For innovative and effective drug development, high-throughput screening needs to be combined with structure-based drug design and chemistry for lead optimization. Fragment screening to identify small, low-molecular-weight organic molecules that bind to a target protein can be achieved in several ways, including surface plasmon resonance (SPR),^17,18^ isothermal titration calorimetry (ITC),^19^ thermal shift assays (TSA),^20^ nuclear magnetic resonance (NMR)^21,22^ and X-ray crystallography.^23,24^ Once a high-resolution structure of the target protein is determined, virtual screens can also be effective.^25^ Recent rapid advances in X-ray data collection automation at synchrotron radiation sources^26^ in combination with automated crystal handling and data analysis work streams such as the XChem platform^27^ at Diamond Light Source make crystallographic fragment screens most attractive. Hundreds or even thousands of compounds can be tested for target protein binding in crystals and structures determined in an efficient way.^28^ Such screens provide atomic detail for ligand orientation and interaction modes, indicating potential routes for fragment growing and chemical tractability for synthesis strategies. Crystallographic fragment screening is highly sensitive with the possibility to identify low affinity (millimolar range) hits, which are unlikely to be false positives.^29^ For these reasons, crystallographic fragment screening has become the gold standard for fragment hit identification and an essential component of fragment-based drug design (FBDD).^30^ Notum has a prominent enzymatic pocket,^7,31^ which affords opportunities for crystallographic fragment screening. By using the Diamond XChem platform with the DSPL library, we identified 59 fragment hits that bind to Notum. These hits were validated by biochemical assays, and several promising hits were chosen for fragment development.

## Results and Discussion

### X-Ray Crystallographic Fragment Screen

The X-ray crystallography-based fragment screen was performed at the XChem platform of Diamond Light Source in combination with synchrotron beamline I04-1 (Didcot, UK). The DSPL fragment library^32^ and Notum core protein^7^ crystals were used. The XChem platform provides a pipeline for crystal drop image analysis, which was used to guide the precise dispensing of compounds within crystal drops (without damage of crystals) by ECHO acoustic droplet ejection.^33^ A “Shifter” device was used to assist crystal harvesting and recording. X-ray diffraction data were collected in unattended mode, processed with Xia2, and analyzed with Pan-Dataset Density Analysis (PanDDA).^34^ The resulting difference maps were used for ligand fitting by Coot.^35^ The identified hits were subject to a Notum enzyme inhibitory assay, and promising hits were chosen for optimization. The general procedure is illustrated in Figure S1. We collected and analyzed 768 data sets, identifying an initial 61 potential hits. Two hits were excluded from later refinement because of inconclusive density for the corresponding ligands. We report here the analysis of all 59 confirmed Notum fragment complexes. All the structures were determined at high resolution (the majority at better than 2 Å), validated, deposited in the PDB, and released (the accession codes are listed in Table 1). The hit compounds vary considerably in their chemical structures (**1**–**59**) with a range of physicochemical properties (*M*_W_ 158–249; clog*P* −0.7 – 3.5; HBD 0–4) and variety in their chemical classes, although the majority are neutral molecules (neutral 47 hits, acids 7 hits, and bases 7 hits); see Table 1 for details.

**Table 1:**
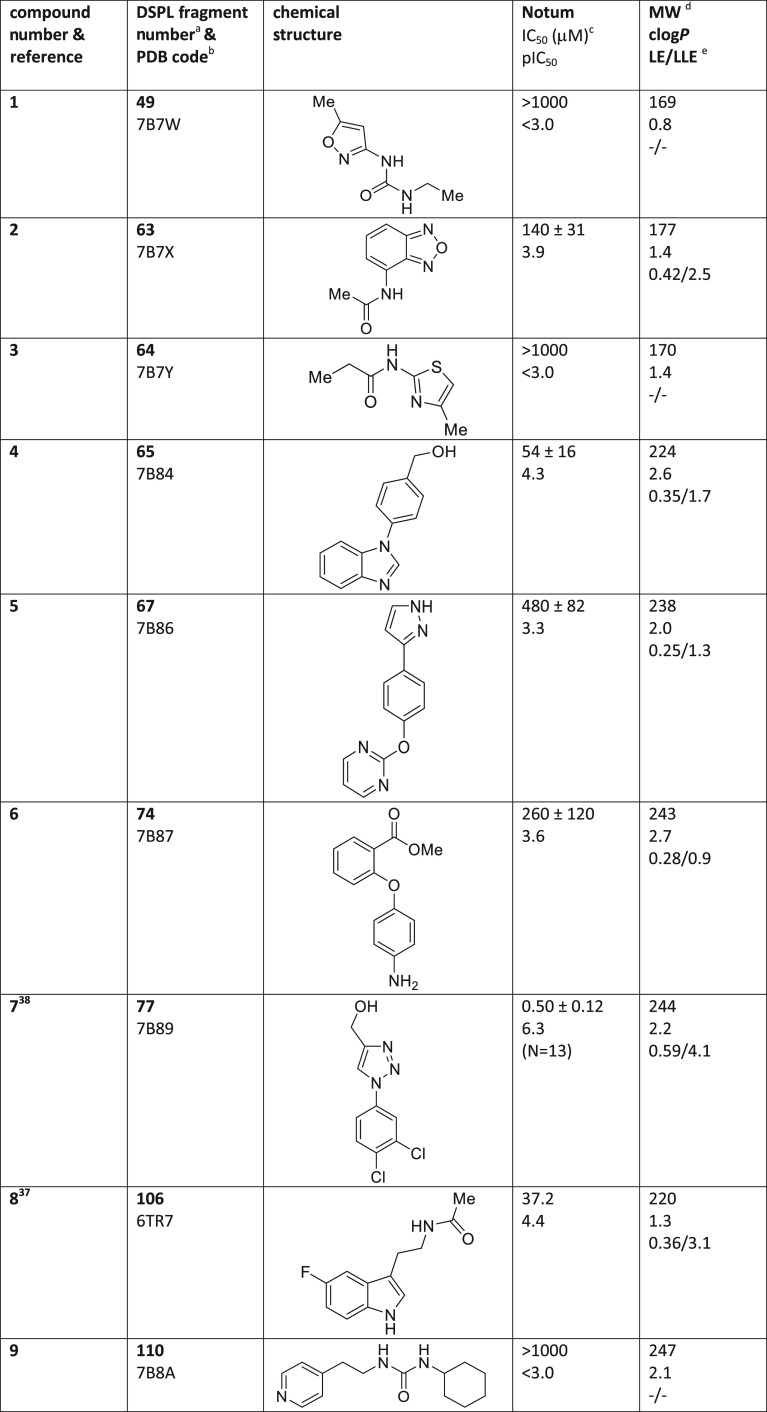

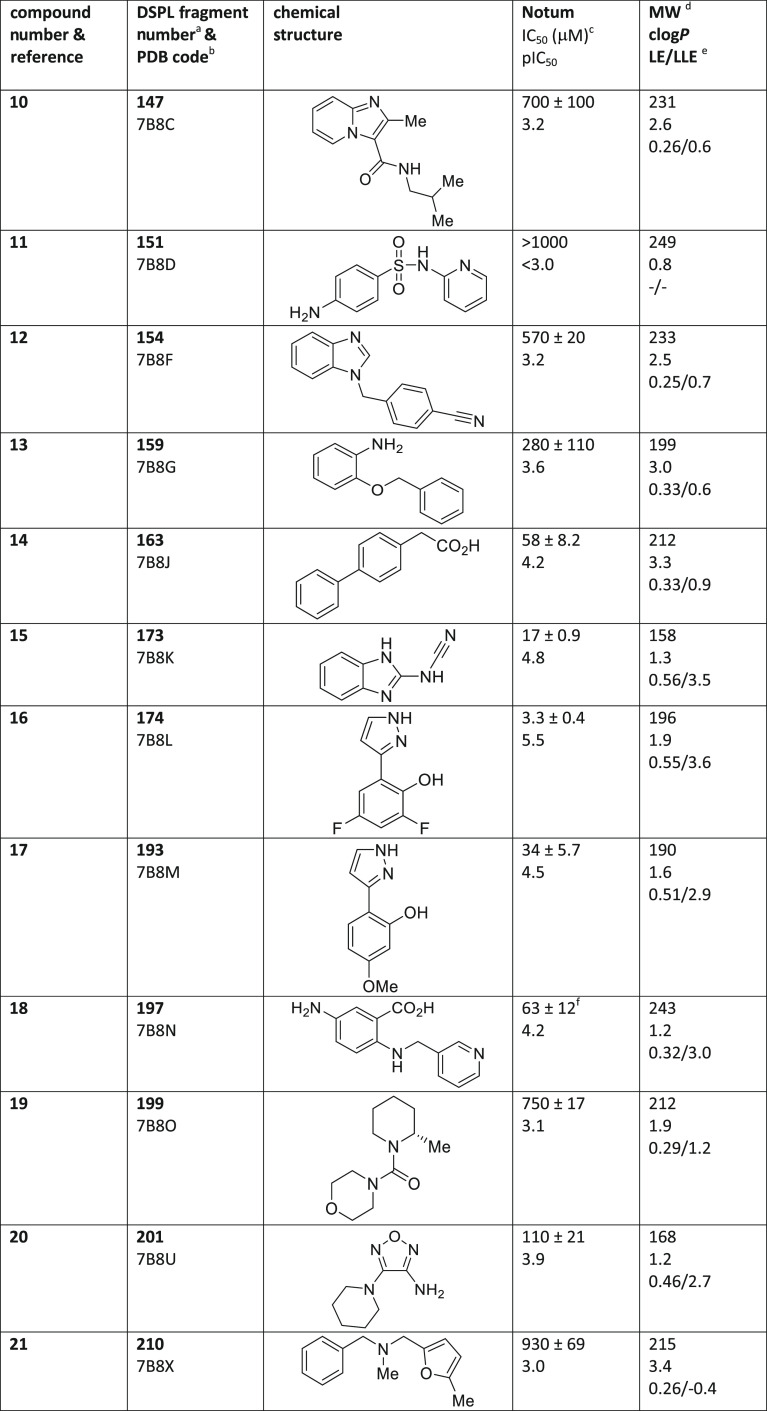

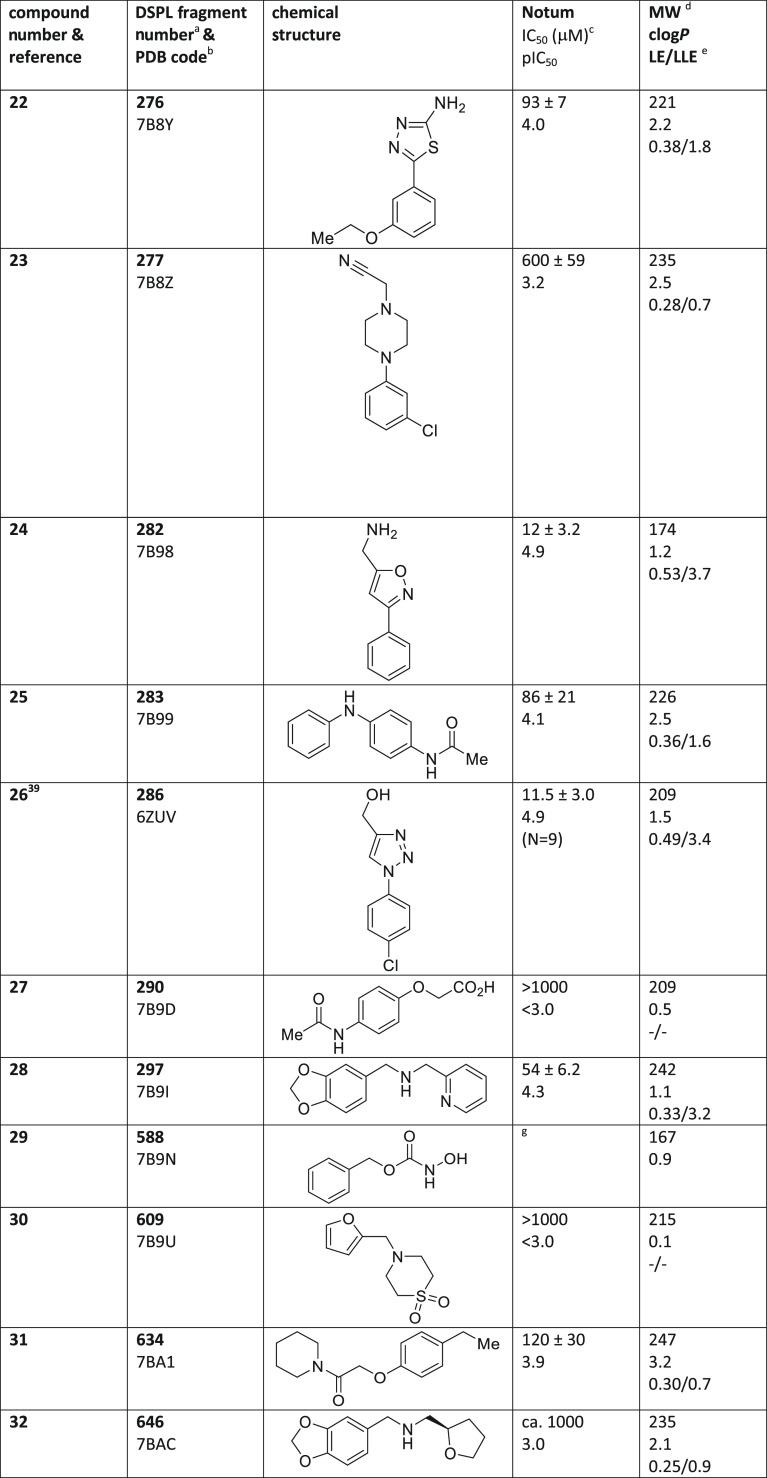

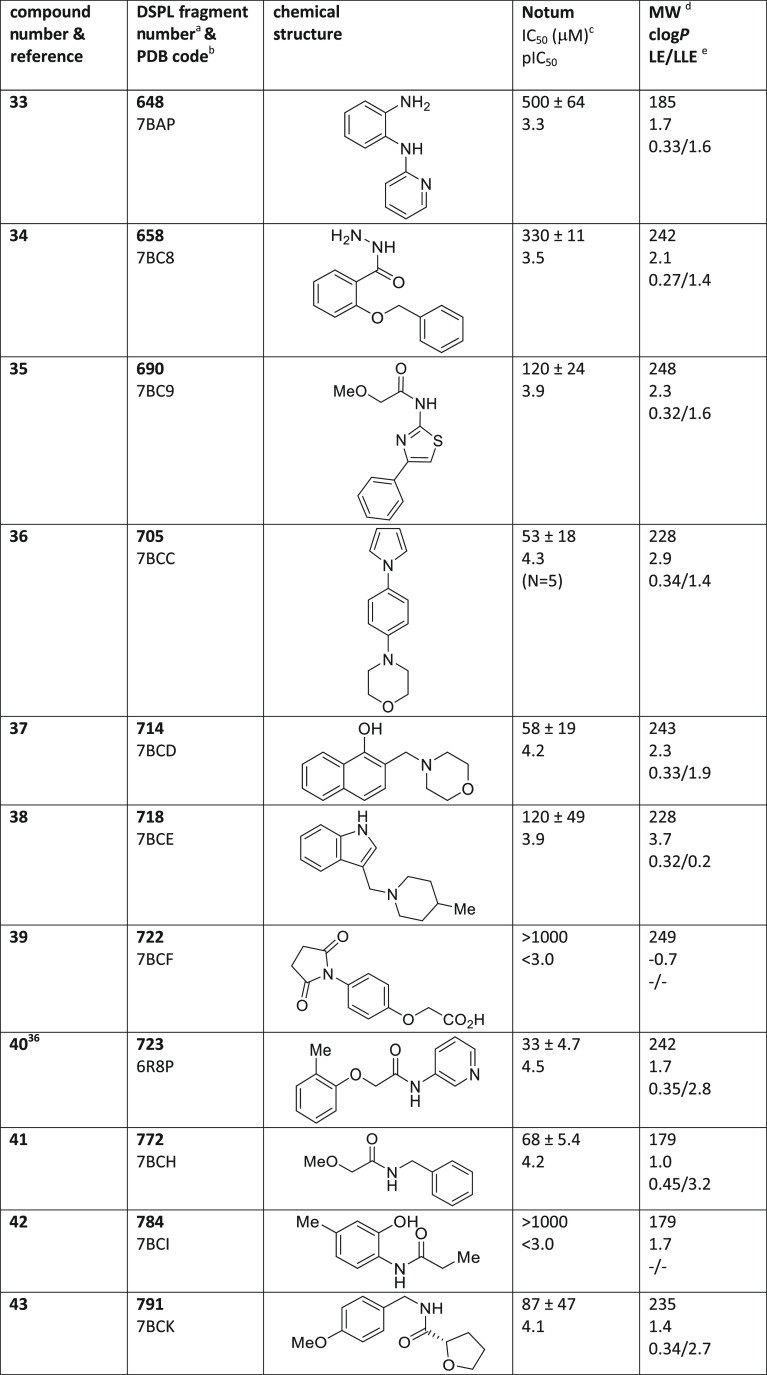

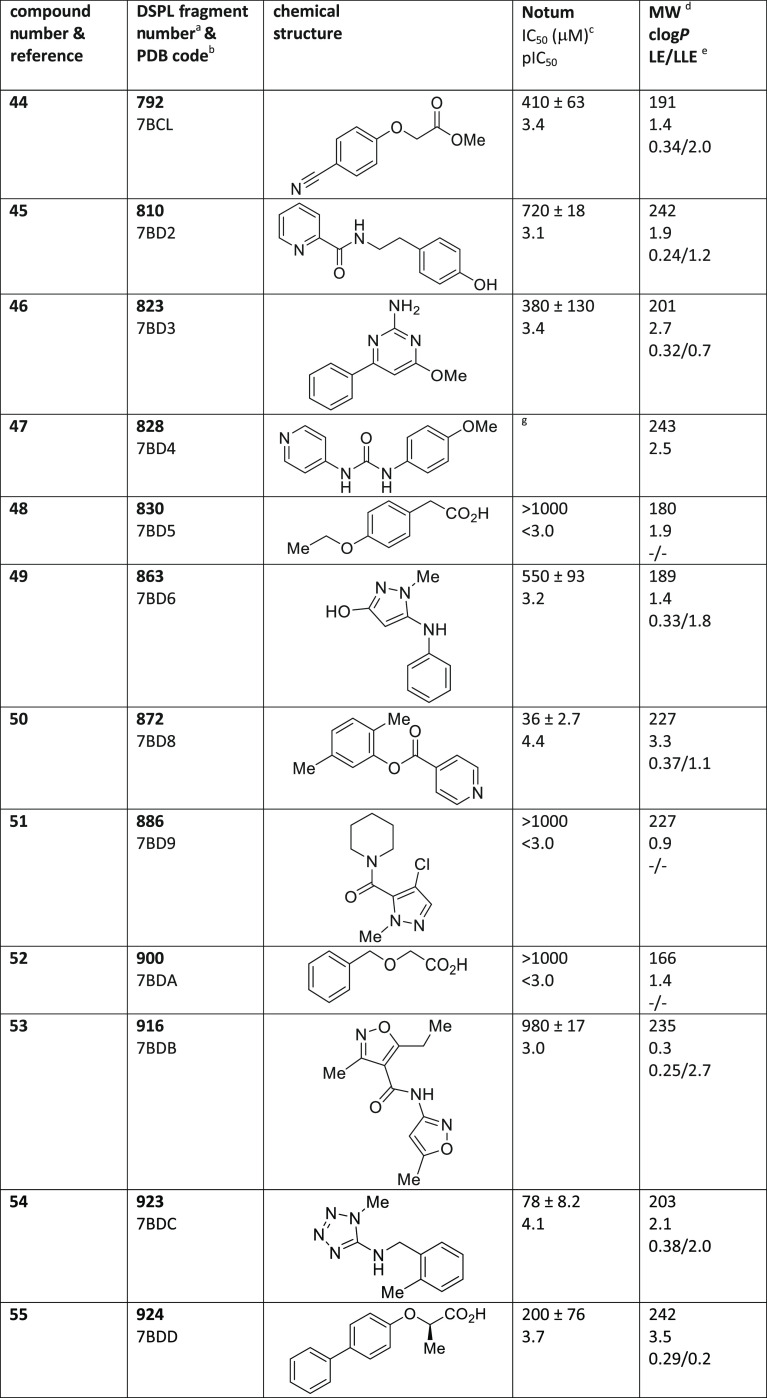

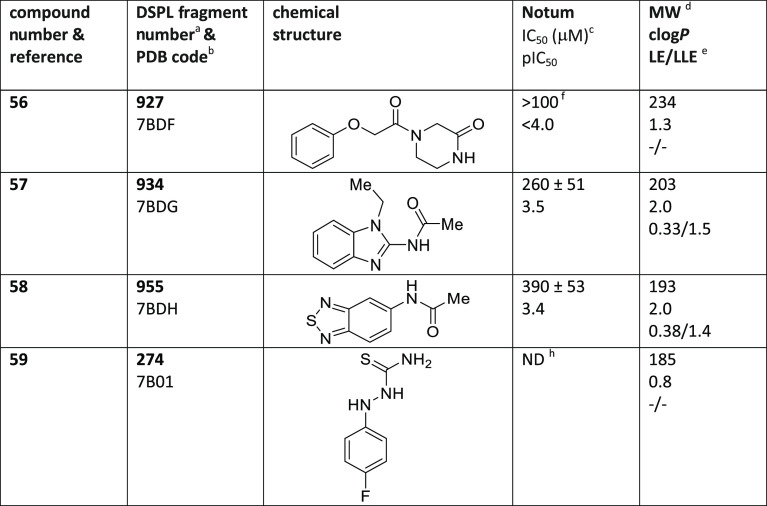
Notum Inhibition for Fragment Hits **1–59**

a Fragment number as annotated in the DSPL.

b Coordinates for these 59 X-ray structures have been published in the PDB.

c IC_50_ values are mean ± s.d. of *N* = 2–4 experiments quoted to 2 s.f. unless stated otherwise. Differences of <2-fold should not be considered as significant. Inhibitors with an IC_50_ > 1000 μM showed 20–45% inhibition @ 1 mM.

d MW and clog*P* calculated with ChemDraw Professional 16.0.1.4.

e Ligand efficiency, LE = 1.4(−pIC_50_)/HAC; lipophilic ligand efficiency, LLE = pIC_50_ – clog*P*.

f Compound showed a variable response upon retesting, IC_50_ > 100 μM.

g Compound illicited a variable, supramaximal response in fluoresence. Fragment was deselected.

h ND, not determined. Fragment was deselected.

### Fragment Hits Localized in the Notum Enzyme Pocket

**Figure 1:**
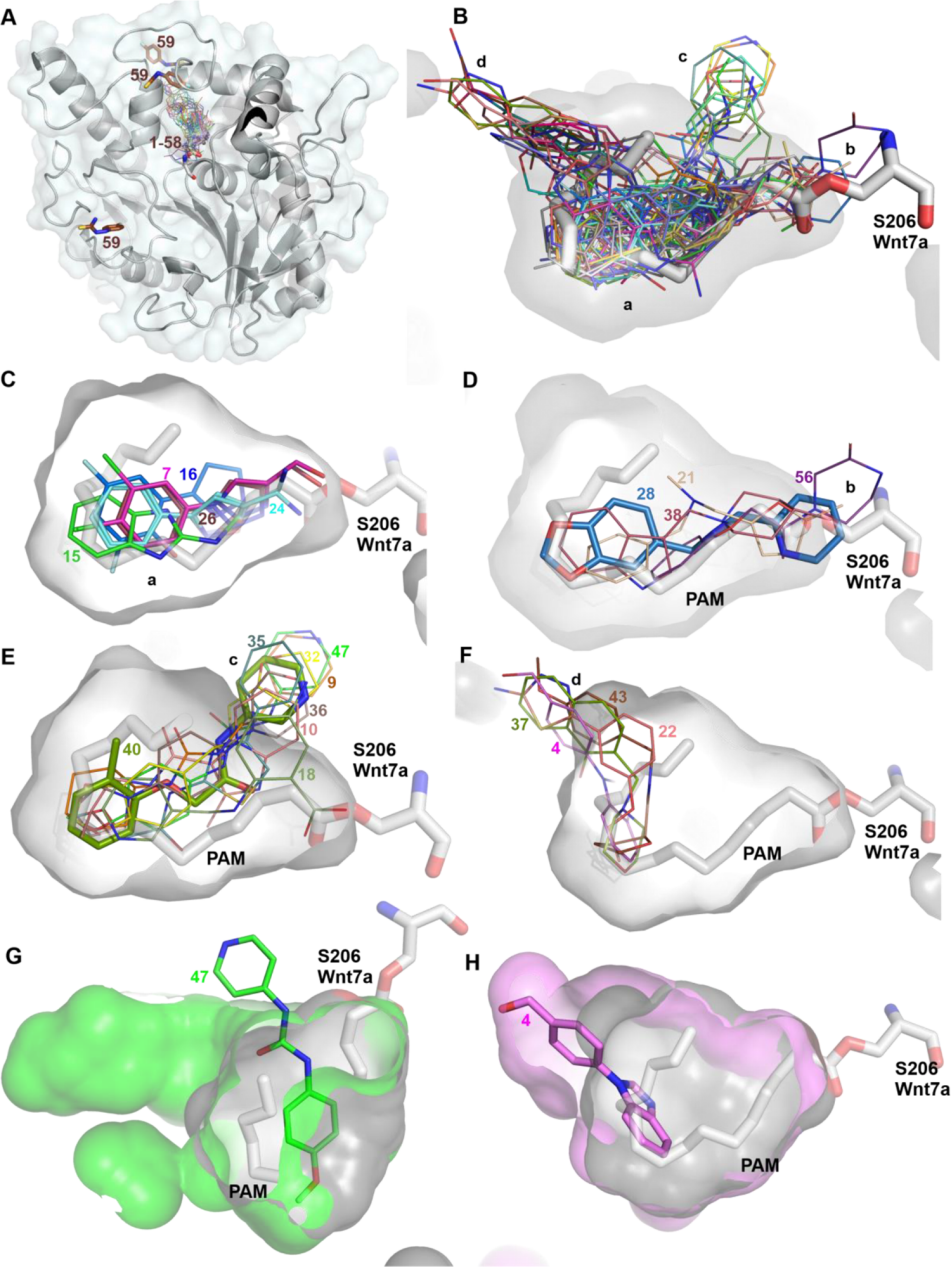
Position of fragment hits within the Notum structure. (A) Notum structure shown as cartoon and surface. All fragment hits (sticks) from complex structures were superimposed alongside the natural substrate (PAM)-bound structure (PDB:4UZQ). (B) Close-up view of the enzyme pocket hits. (C–F) Subpocket positions are clustered into four groups. The natural substrate (the PAM linked Wnt7a serine-206) is shown as thick gray sticks. Thinner fragment sticks indicate compounds with poor potencies (IC_50_ > 100 μM); thicker sticks indicate good potencies (IC_50_ < 100 μM). (G and H) Overlay of the Notum pocket bound to PAM (gray) and fragment **47** (green) or **4** (pink). All structures are deposited in the PDB with access codes listed in Table 1.

Among the 59 hits identified, only one (**59**) was found to not bind inside the enzyme pocket and was instead observed to bind at three other locations (Figure 1A). All other hits (**1**–**58**) were found bound exclusively within the pocket or with at least one molecule in the enzyme pocket. The majority of the hits are observed in the central area of the pocket (position a, Figure 1B, C), where they overlap with the binding site of the palmitoleate (PAM) lipid group covalently linked to Wnts. Hits with high potency (IC_50_ < 20 μM) tend to cluster in this position, including **7**, **15**, **16**, and **24 (**Figure 1C). Thus, hits from this cluster represent clear candidates for optimization. Some hits (**21**, **28**, **38**, **56**) show atoms that extend toward the entrance of the pocket (where the PAM-serine linkage is observed, position b, Figure 1B, D), but retain several interactions with the central pocket residues. Among these, **28** shows a modest IC_50_ value and was chosen for optimization. Another group of hits (**9**, **10**, **18**, **32**, **35**, **36**, **40**, **47**) extended toward the interior boundary of the pocket (position c, Figure 1B, E). Hit **40** from this group was subjected to optimization as reported.^36^ The fourth group extends toward the base of the pocket (position d, Figure 1B, F). This group of hits (**4**, **22**, **37**, **43**) showed IC_50_ values between 50 and 100 μM. This group of hits causes an expansion of the pocket volume, as seen for hit **4** in Figure 1H, which offers potential for exploring the chemical space created by induced fit. It is noteworthy that in a few compound-bound Notum structures, like **47**, some surrounding pocket residues become disordered, and the pocket becomes artificially expanded (Figure 1G). This kind of pocket volume, formed by the disordered residues, may be not useful for proactive structure-guided design.

### Hit Validation and Selection

Fragment hits were validated as inhibitors of Notum enzymic activity in a biochemical assay. All hit compounds were synthesied or purchased as authentic solid samples (see the Supporting Information), except **59**, which was deselected because of its nonpocket occupation. The inhibition of enzyme activity (IC_50_ value) was calculated from 10-point concentration-response curves with compound concentrations ranging from 30 nM to 1 mM (inhibition–concentration curves for compounds will be made available upon request). Two compounds, **29** and **47**, demonstrated variable, supramaxial responses in fluoresence at higher concentrations (>100 μM), which is the opposite outcome expected for a Notum inhibitor in this assay. Inspection of their chemical structures suggests that this outcome was most likely because of assay interference rather than enzyme activation. Ultimately, these two compounds would need to be screened with alternative technologies to determine their mode of action and were simply deselected as better options were available.

Twenty fragments had an IC_50_ < 100 μM with two examples, **7** and **16**, showing inhibition of Notum activity with an IC_50_ < 10 μM (Table 1). 1-Phenyl-1,2,3-triazole **7** (IC_50_ 0.5 μM) was the most active fragment from the screen and anchored a small cluster of six structurally related phenyl azoles (**7**, **16**, **17**, **22**, **24**, **26**). Additional azoles and azines (e.g., **5**, **35**, **36**, **46**), identified as weaker hits, provided some insight into preliminary SARs around this cluster.

**Figure 2:**
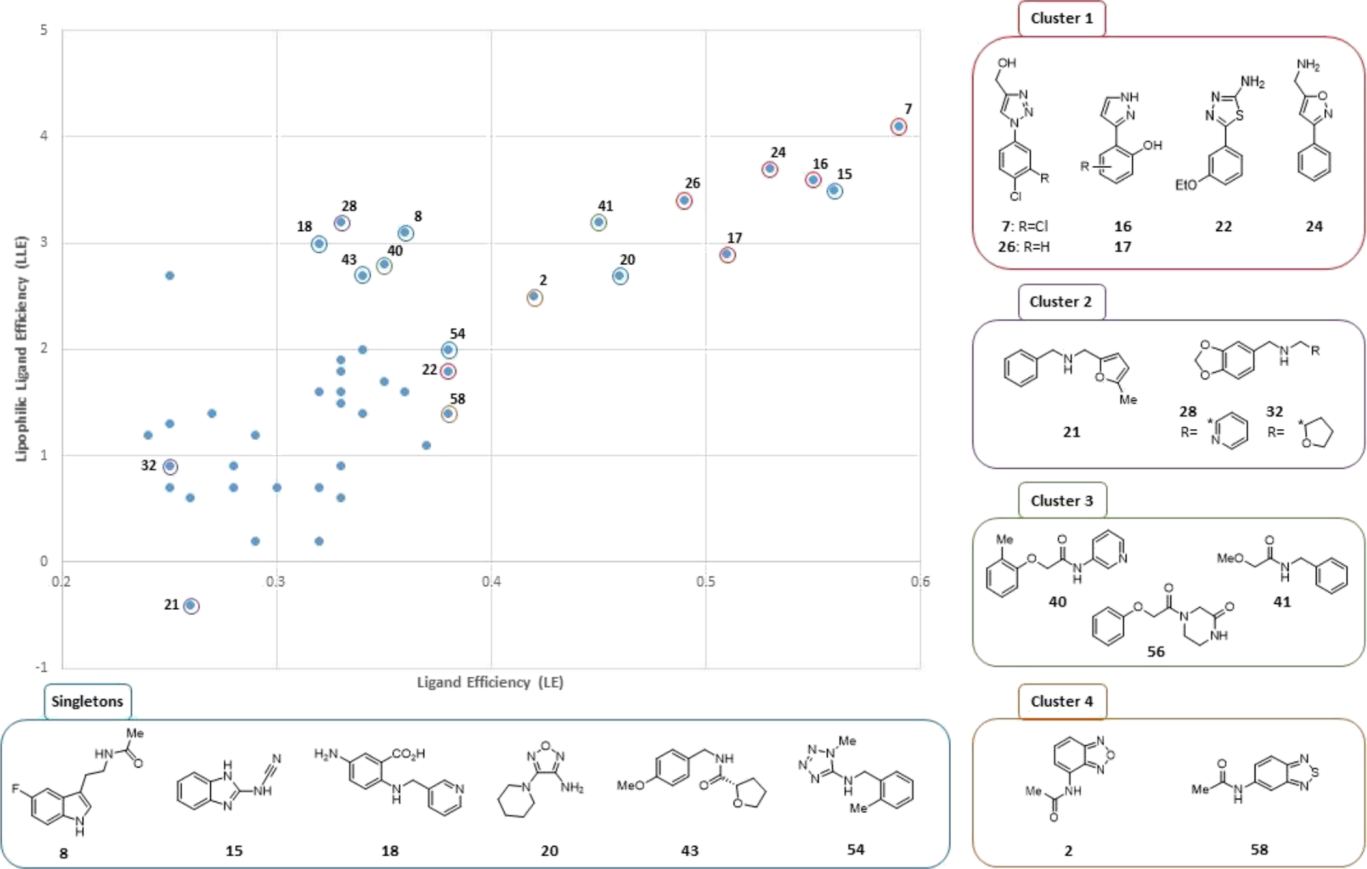
Plot of LLE vs LE for fragment hits. Plot only includes Notum inhibitors with a measured IC_50_ ≤ 1 mM.

In addition, the dataset was analyzed by the standard design metrics ligand efficiency (LE)^20^ and lipophilic ligand efficiency (LLE)^21^ in order to tease out smaller, less lipophilic hits that may also prove to be attractive starting points for fragment development (Figure 2). A plot of LLE vs LE clearly showed the phenyl azoles (cluster 1: LE ≥ 0.3, LLE ≥ 3) to be superior, and, interestingly, the majority of inhibitors in this cluster are structurally positioned in the central part of the enzyme pocket (position a, Figure 1C). This analysis also identified additional clusters and singletons for further investigation.

1,2,3-Triazole **7**, and close analogue **26** (IC_50_ 11.5 μM),^23^ became the main focus of our fragment development activities along with other members of cluster 1 (**16**, **24**). Limited efforts were made to investigate cluster 2 (**28**: IC_50_ 54 μM) as it was the least attractive option by LE and LLE. Cluster 3 (**40**: IC_50_ 33 μM) was also selected as a preferred hit because the template was highly chemically enabled with three points of diversity and offered the opportunity to quickly explore SAR to improve activity.^19^ Singleton benzimidazole **15** (IC_50_ 17 μM) scored highly in terms of both LE and LLE, and was selected for optimization. These six hits were simultaneously explored to evaluate their potential to deliver a potent inhibitor of Notum.

Singleton hit *N*-[2-(5-fluoro-1*H*-indol-3-yl)ethyl]acetamide (**8**: IC_50_ 37.2 μM) is closely related to the hormone melatonin, which was also shown to bind to Notum but with slightly weaker affinity (IC_50_ 75 μM; 6TR5). Full details of the ligand-bound structures with Notum and biophysical characterization of the ligand-Notum interactions have been reported.^37^

Resource limitations meant it was not possible to simultaneously explore all the hits, and so some clusters and singletons were paused to be reexamined should the front runners fail to deliver an advanced lead with the desired profile. Hits for fragment development were selected, or paused, based on the following criteria: IC_50_, LE, LLE, synthetic accessibility, multiple points of structural diversification to create SAR, and absence of metabolic or structural liabilities. Fragment hits **2** (IC_50_ 140 μM), **20** (IC_50_ 110 μM), and **43** (IC_50_ 87 μM) could also have been good starting points for development had they not been overshadowed by preferred hits **7** (cluster 1) and **40** (cluster 3).

### Fragment Development

#### 1,2,3-Triazoles

**Figure 3:**
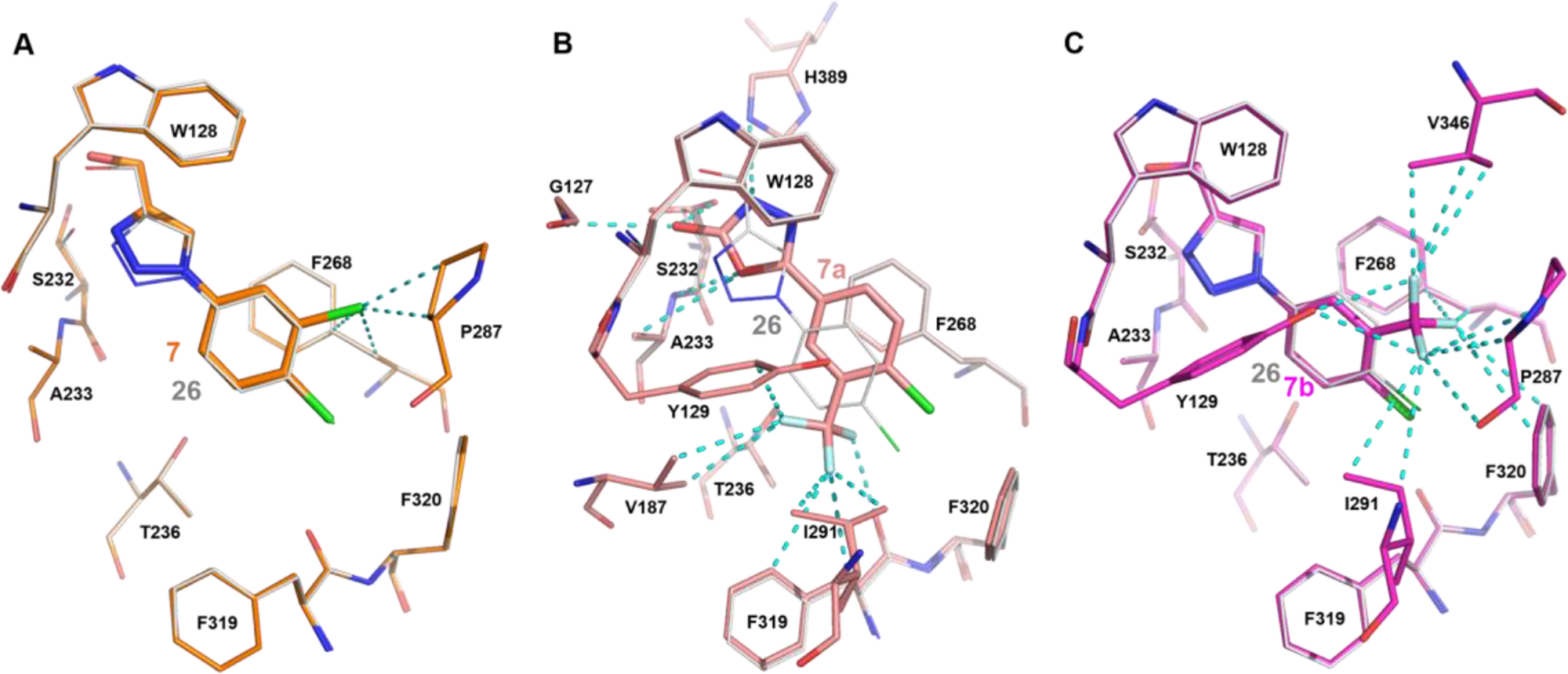
Notum ligand interaction details. (A) Comparison hits **7** and **26**. The compounds and Notum interaction residues are shown as sticks (gray for **26**, orange for **7**). (B) Comparison of **26** (gray sticks) and **7a** (salmon sticks). (C) Comparison of **26** (gray sticks) and **7b** (magenta sticks). The dash lines indicate the gained interaction by **7**, **7a**, and **7b** in comparison to **26**. All the compounds here form π–π stacking interactions with residues F268 and W128.

A pair of remarkable hits were 4-(hydroxmethyl)triazoles **7** and **26**, which also highlighted some early SAR.^36,39^ A 3-Cl-4-Cl disubstituted phenyl ring (**7**) conferred a 23-fold improvement in Notum inhibition when compared to a single 4-Cl (**26**). Structurally, the 3-Cl gained hydrophobic interaction with P287 and strengthened the interaction with F268 (Figure 3A). Triazole **7** has properties consistent with lead-like chemical space and scored highly when assessed by design metrics (LE 0.59; LLE 4.1), including a favorable prediction of brain penetration.^40−42^ Triazole **7** was further assessed in standard in vitro assays to determine its ADME properties and showed good aqueous solubility (100 mg/mL), moderate stability in liver microsomes (MLM, Cl_i_ 88 mL/min/mg protein; HLM, Cl_i_ 12 mL/min/mg protein), and excellent cell permeability (MDCK-MDR1, AB/BA *P*_app_ 57/59 × 10^–6^ cm/s, ER 1.0).^38^ The development of **26** by modifying the heterocyclic head group, along with the exploration of SAR of the phenyl ring, identified two complementary lead series: 1,3,4-oxadiazol-2(3*H*)-ones (**7a**)^39^ and 1,2,3-triazoles (**7b**).
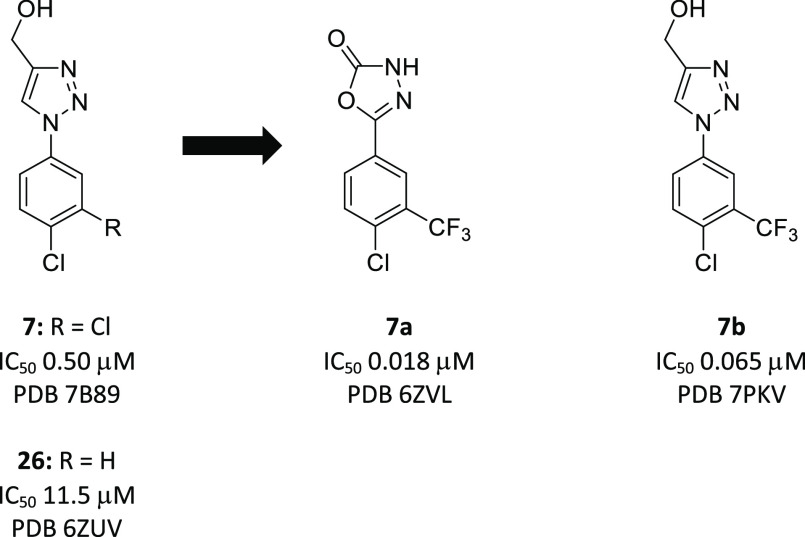


A systematic investigation of substitution on the phenyl ring in the oxadiazol-2-one series identified **7a** as a potent inhibitor.^39^ The mouse pharmacokinetic studies demonstrated good plasma exposure but only partial brain penetration.^39^ Structurally, the oxadiazole head group gains interactions with G127, H389, A233, and S232. These interactions cause the phenyl ring to be flipped in comparison to 3-Cl of **7**. The 3-CF_3_ gains a lot of interactions with the Notum pocket residues Y129, F319, and I291 (Figure 3B). These residues are located on the opposite side of the pocket when compared to the 3-Cl interaction residues of **7** (P287, F268, Figure 3A).

Comparison of preferred substitution patterns of the phenyl ring across several Notum-inhibitor chemotypes found that 3-CF_3_–4-Cl groups could give potent activity. Available structural information showed that these inhibitors tend to occupy the palmitoleate pocket more completely.

Application of the preferred 3-CF_3_–4-Cl substituents to the 1,2,3-triazole series gave **7b** as a credible early lead with improvement in the Notum inhibition activity (IC_50_ 0.065 ± 0.040 μM). Compound **7b** restored Wnt/β-catenin signaling in a cell-based TCF/LEF (Luciferase) reporter assay in the presence of Notum (EC_50_ 1.6 ± 1.2 μM)^39^ and, as expected, had similar in vitro ADME properties to **7**. Structurally, the 3-CF_3_ of **7b** conferred the same phenyl ring orientation as the 3-Cl of **7** retaining interaction of P287 and F268 but gained additional interactions with residues V346, F320, and I291 (Figure 3C). Compared to **7a**, of which 3-CF_3_ being on the opposite of the pocket, it suggests the head group of oxadiazol or triazole may influence the phenyl group interaction.

#### Pyrrazoles

**Figure 4:**
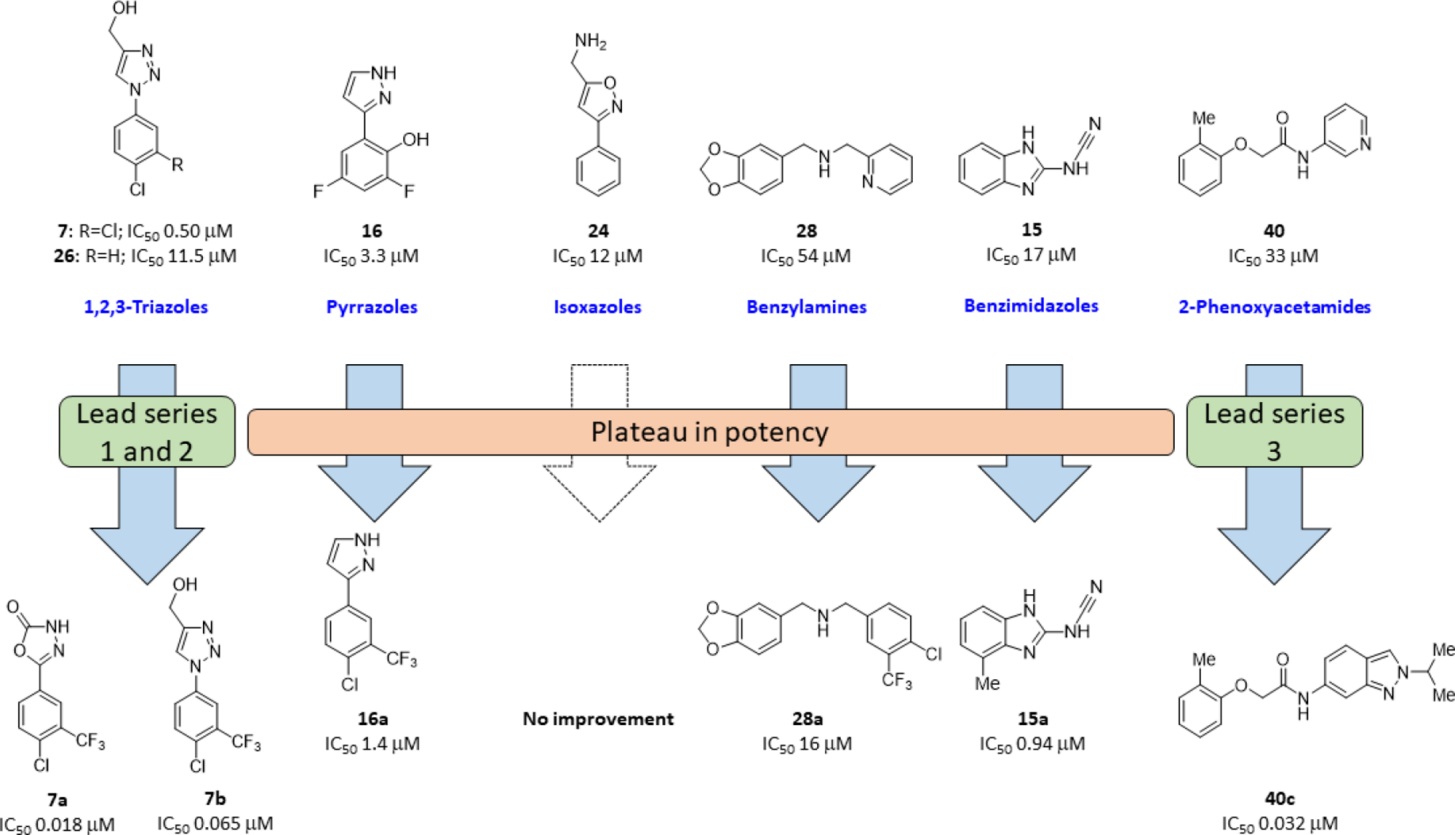
Summary of fragment development of six hits **7**/**26**, **16**, **24**, **28**, **15**, and **40**.

**Figure 5:**
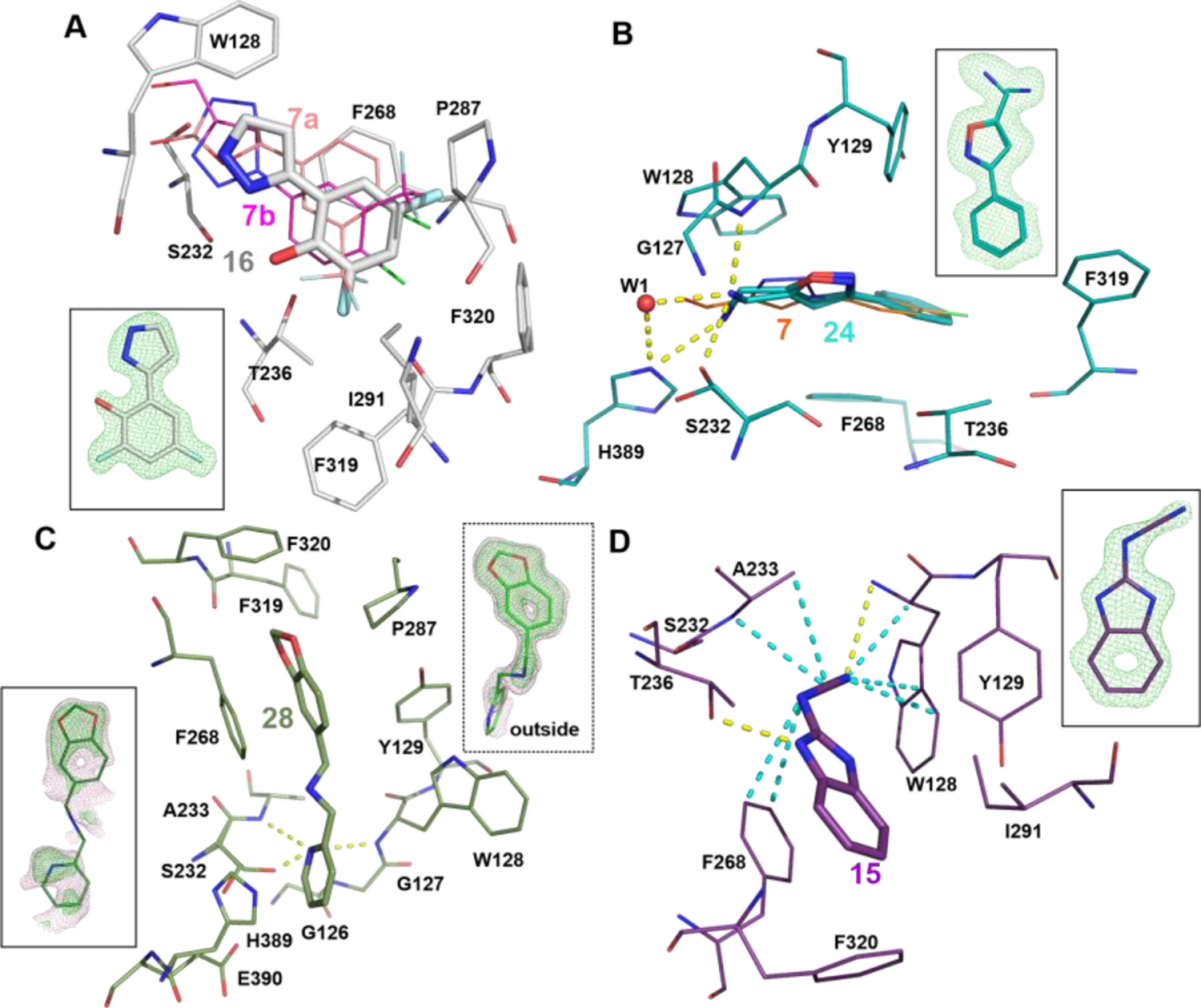
Notum fragment interaction details and |Fo–Fc| omit maps (green mesh at the contour level of 3σ, or pink at 2σ). (A) Hit **16** (gray sticks) forms hydrophobic interactions with Notum pocket residues. Aligned **7a** (salmon) and **7b** (magenta) are shown as thin sticks. (B) Hit **24** (light teal sticks) interaction details. Yellow dash lines indicate the polar interactions. Catalytic water (W1) is shown as a red sphere. (C) Hit **28** (dark green sticks) interaction details. There are two copies of **28**. The outside pocket copy of the omit map is shown as green sticks with a hash-lined box. (D) Hit **15** (purple sticks) interaction details. The cyanamide group mediated hydrophobic interactions were highlighted as cyan dash lines. All the compounds within the pocket form π–π stacking interactions with residue F268.

Initial efforts to improve **16** focused on N1 methylation and SAR of the aryl ring by substitution with halogens (F, Cl) (Table S1). Only the introduction of 3-CF_3_–4-Cl groups **16a** (IC_50_ 1.4 ± 0.1 μM) gave an incremental improvement in activity (2.3-fold) (Figure 4). Direct comparison of **16a** with alternative heterocyclic head groups showed the pyrazole to be significantly inferior to both the oxadiazol-2-one **7a** and 1,2,3-triazole **7b**. Comparison of the binding modes of **16** with **7a** and **7b** by superimposing their structures show that the 3-F and 5-F of **16** overlap with the CF_3_ group of **7b** and **7a**, respectively, gaining interactions with both sides of the pocket (Figure 5A).

#### Isoxazoles

Isoxazole **24** also belongs to cluster 1 and demonstrates good inhibition of Notum activity (IC_50_ 12 μM) (Figure 2). Structurally, the aminomethyl group shows two conformations (Figure 5B) and forms extensive polar interactions with the catalytic triad residues S232, H389 and the oxyanion residue W128, as well as catalytic water (Figure 5B). This catalytic water 1 (W1) may coordinate protonation/deprotonation of the catalytic triad and is important for the enzymatic action.^43^ Isoxazole **24** is positioned similarly to **7**, showing good hydrophobic interactions with other pocket residues, including F268, F319, Y129, and T236 (Figure 5B). However, despite its promise as a hit for fragment development, a small set of analogues that explored halogen substitution on the aryl ring or alternatives to the 5-aminomethyl group offered no improvement over **24** and were inferior to the triazoles.

#### Benzylamines

Benzylamine **28** was the preferred hit from cluster 2 albeit with weaker activity (IC_50_ 54 μM) compared to other clusters selected for hit development. The X-ray data refinement identified two copies of **28** bound to Notum; one was inside the enzyme pocket with very poor electron density (Figure 5C) and the other copy was located outside the pocket, bridging another crystal packing promoter. These non-pocket binders are unlikely to contribute to the inhibition of Notum’s enzyme activity as we observed previously,^37^ and so, only the interactions of the pocket binder copy were investigated (Figure 5C). The virtual chemical space of bis(benzyl)amines is incredibly large based on reliable chemical methods for their construction and available synthetic monomers. This could be further expanded by placing a third group on the nitrogen atom. Rather than initiate a large combinatorial approach, we elected to make a small pilot set of bis(benzyl)amines to establish their potential to compete with the triazole series. The benzodioxol-5-ylmethyl group was common to the two hits in cluster 2 and so was retained to provide single point changes with the variation of the second substituent. However, most changes were detrimental with only the 4-chloro-3-(trifluoromethyl)benzyl group **28a** (IC_50_ 16 ± 1.5 μM), providing a small increase in activity but at the expense of significant added lipophilicity (clog*P* 4.0) (Figure 2, Table S1).

#### Benzimidazoles

Benzimidazole **15** was selected as a singleton with good potency (IC_50_ 17 μM) and LE/LLE values similar to the leading members of cluster 1 (Figure 2). The benzo ring of **15** forms a ring-ring stack interaction with F268 and hydrophobic interactions with F320, I291, Y129, and W128 (Figure 5D). The cyanamide polar group (−NHCN) forms a polar interaction with oxyanion residue W128 and extensive hydrophobic interactions with W128, A233, and F268 (Figure 5D). New analogues were all prepared with the retention of the cyanamide group at C2 to retain these favorable interactions. Efforts to improve activity were limited to the exploration of benzimidazole at the N1, C4, C5, and C7 positions; combinations of preferred groups (N1-Me, 4-Me, 4-Cl) were not synergistic (Table S1). The most active inhibitor from this limited set was the simple 4-methyl analogue **15a** (IC_50_ 0.94 ± 0.06 μM), which represented a modest 18-fold increase in Notum activity accompanied by small gains in LE and LLE (Figure 2). However, there emerged a plateau in Notum inhibition with this series at around 1 μM.
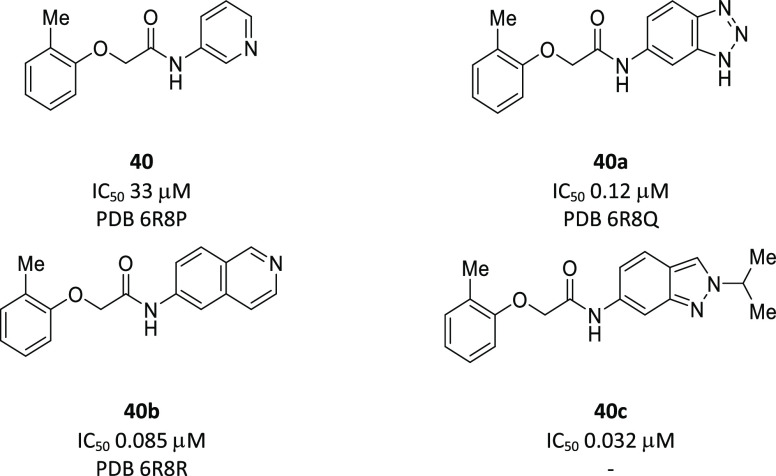


#### 2-Phenoxyacetamides

Fragment development of **40** from cluster 3 has been initiated earlier.^36^ Fragment **40** (IC_50_ 33 μM) was identified with poor density, but its analogues improved the ligand electron densities, following the replacement of the pyridine ring with benzotriazole (**40a**, IC_50_ 0.12 μM) and isoquinoline (**40b**, IC_50_ 0.085 μM).

Further optimization of hit **40** by SAR studies of the aryloxy ring, acetamide backbone, and amide group, guided by several Notum-inhibitor structures, gave indazole **40c** (IC_50_ 0.032 μM) as the most potent inhibitor from this series. However, it was not possible to combine Notum activity with metabolic stability as measured in mouse and human liver microsomes. It was of note that these compounds were metabolized in an NADPH-independent manner.^36^

**Figure 6:**
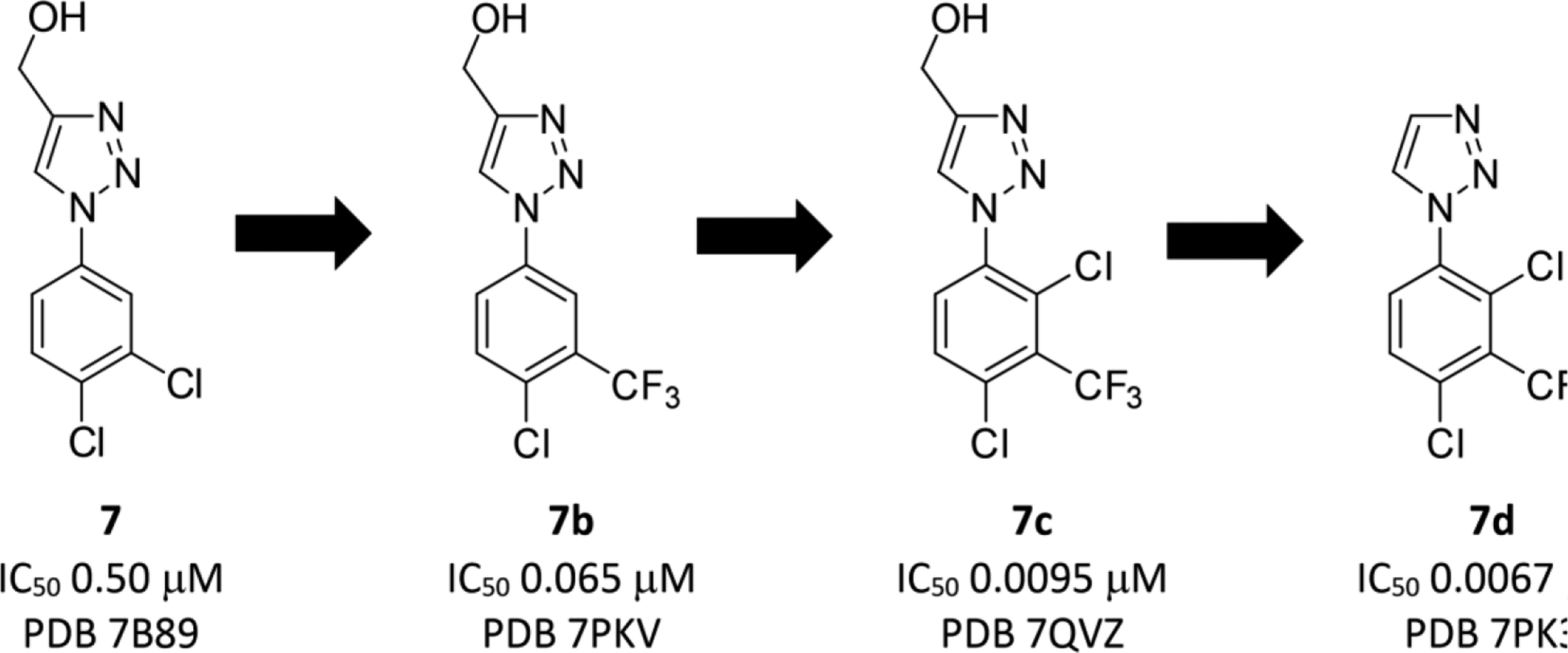
Optimization of fragment hit **7** through to **7d**.

With a plateau in potency at around 1–10 μM for the pyrrazoles **16**, bis(benzyl)amines **28,** and benzimidazoles **15**, and the 2-phenoxyacetamides **40** having poor metabolic stability, our efforts became entirely focused upon further optimization of the 1,2,3-triazole series **7** (Figure 6).

### Fragment Hit to Advanced Lead

**Figure 7:**
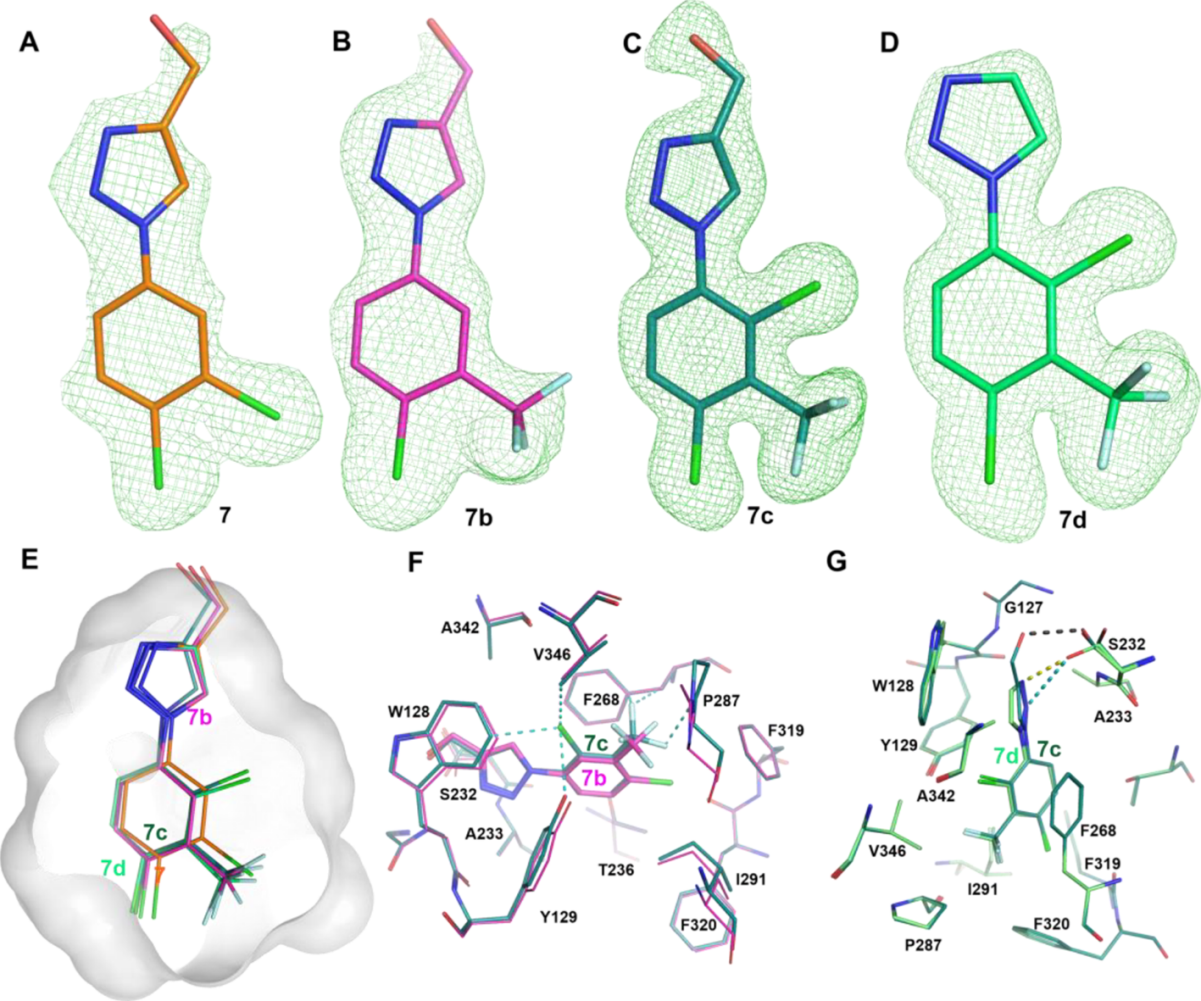
Structural character of **7d** development. (A–D) The |Fo–Fc| omit electron density maps (green mesh contoured at 3σ) for **7** (7B89, orange), **7b** (7PKV, magenta), **7c** (7QVZ, teal), **7d** (7PK3, light green), respectively. (E) Superimposed **7b**, **7c**, and **7d** complex structures. The **7d** pocket is outlined as the gray surface. (F) Interaction details of **7c** (teal sticks) with comparison to **7b** (magenta sticks). Cyan dash lines indicate **7c** gained hydrophobic interactions. (G) Interaction details of **7d** (green sticks) with comparison to **7c** (teal sticks). Dash lines indicate that **7d** gained polar (yellow) or hydrophobic (cyan) interactions, or the lost polar interaction (black) compared to **7c**. All the compounds here form π–π stacking interactions with residue F268.

Further development of **7b** through the introduction of aryl substituents that more optimally fill the palmitoleate pocket gave **7c**, and then deletion of the hydroxymethyl group identified **7d** as a potent inhibitor of Notum activity (IC_50_ 0.0067 ± 0.0016 μM) (Figure 7). Full details of these SAR studies, along with the profile of **7d**, have been presented in a recent publication.^38^

Structurally, all members of the 1,2,3-triazole series (**7**, **7b**–**d**) were crystallographically resolved at high resolution with good data collection and refinement statistics, and their omit maps are shown in Figure 7A-D. Oxadiazole **7a** has been published through optimization of **26**.^39^ The introduction of a 2-Cl on the aryl ring of **7b** gave **7c**, which further improved potency by 7-fold. Analysis showed that **7c** maintained the same position and orientation as **7b** within the pocket (Figure 7E). The newly added 2-Cl group gained hydrophobic interaction with W128, Y129, and V346 (Figure 7F). In addition, the 3-CF_3_ group of **7c** was repositioned such that one fluorine atom interacts with F268, which is already participating in a ring-ring stack interaction with the phenyl ring, while one other fluorine atom of the CF_3_ gains interaction with P287(Figure 7F).

Triazole **7d** was designed to improve pharmacokinetic properties in vivo by reducing clearance through phase 2 metabolism of the primary hydroxyl group, but it also became the most potent inhibitor from this series despite removal of the −CH_2_OH group.^38^ From the structure point of view, despite the loss of the hydrogen bond of the −CH_2_OH group compared to **7c**, the catalytic residue S232 presents an alternative rotamer conformation and forms a new hydrogen bond with -1N as well as interaction with -2N of the triazole head (Figure 7G). These additional interactions contribute to further increasing potency, and **7d** proved to be a potent inhibitor of Notum activity inhibition in both biochemical (OPTS) and cell-based reporter (TCF/LEF) assays (IC_50_ 0.0067 ± 0.0016 μM and EC_50_ 0.110 ± 0.100 μM, respectively).

### Synthesis of Inhibitors

Consistent with the design of the DSPL being a “poised” fragment library, the synthesis of hits and close analogues during hit development was a straightforward process. The chemistry was established, reliable, and involved relatively short synthetic sequences of usually just one step or telescoped routes. The resupply of the selected hits **1–58** as authenticated, solid samples was accomplished by purchase from commercial vendors (19/58) or resynthesis using standard methods (39/58), see the Supporting Information.

**Scheme 1:**
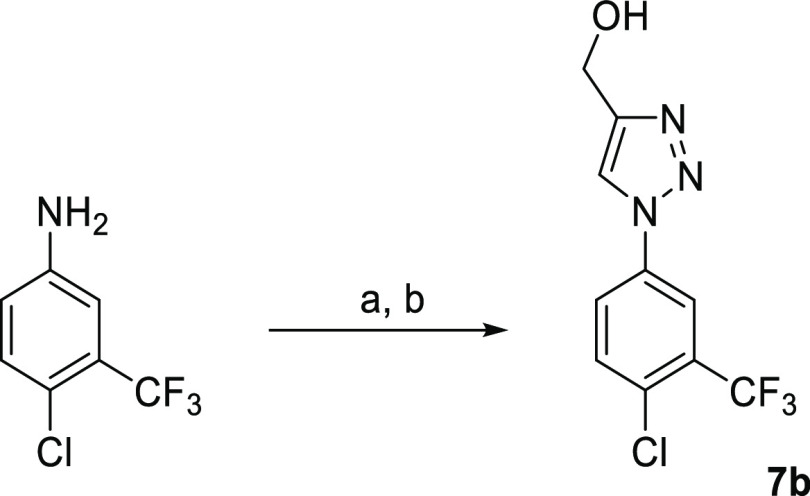
Synthesis of 1,2,3-Triazoles **7b** Reagents and conditions: (a) (i) NaNO_2_ (1.2 equiv), CF_3_CO_2_H, 0 °C → RT, 1.5 h; (ii) H_2_O, RT → 0 °C, (iii) NaN_3_ (1.1 equiv), 0 °C → RT, 1 h; (b) HC≡CCH_2_OH (1.0 equiv), sodium l-ascorbate (0.4 equiv), CuSO_4_·5H_2_O (0.2 equiv), *t*BuOH-H_2_O, 50 °C, 2 h, 83%.

General methods for the synthesis of new inhibitors during the fragment development phase (**7b**, **15a**, **16a**, and **28a**) are presented in Schemes 1^2^,3,4, illustrated with the most active inhibitor from the set of analogues (Figure 4 and Table S1). Detailed methods for the synthesis of 1,3,4-oxadiazol-2(3*H*)-ones **7a**,^39^ 1-phenyl-1*H*-1,2,3-triazoles **7c** and **7d**,^38^ and 2-phenoxyacetamides **40a**, **40b,** and **40c**(36) have been published.

**Scheme 2:**
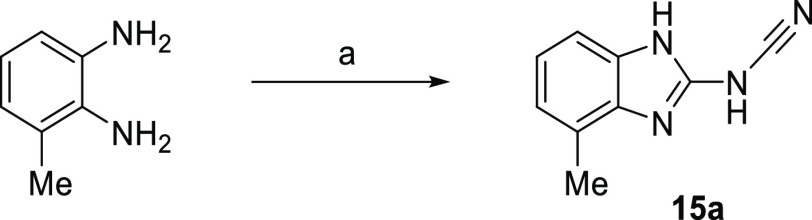
Synthesis of Benzimidazole **15a** Reagents and conditions: (a) (PhO)_2_C=N.CN (1.0 equiv), *i*PrOH, 50 °C, 3 h, 33%.

**Scheme 3:**
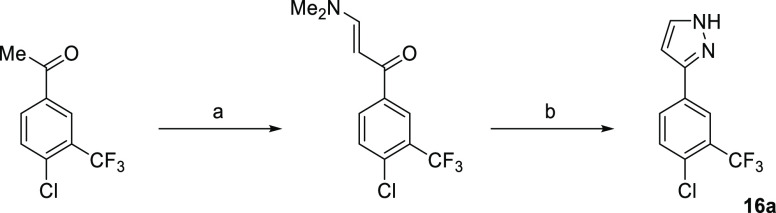
Synthesis of Pyrrazole **16a** Reagents and conditions: (a) Me_2_NCH(OMe)_2_ (3 equiv), DMF, 90 °C, 2 h; (b) NH_2_NH_2_·H_2_O (10 equiv), MeOH, 68 °C, 18 h, 41% over two steps.

**Scheme 4:**
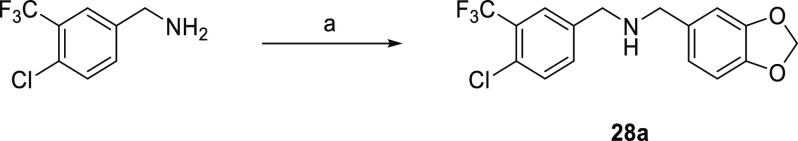
Synthesis of Bis(benzyl)amine **28a** Reagents and conditions: (a) (i) piperonal (1.2 equiv), MeOH, RT, 30 min; (ii) NaBH_4_ (1.2 equiv), RT, 4 h, 68%.

## Conclusions

X-ray crystallographic fragment screening provided an effective way for the identification of a large number of novel fragments bound to Notum. The three-dimensional atomic detail of ligand orientation and interaction modes from the 58 compound-Notum complexes provides a rich seam of information for Notum-inhibitor design and development. The pocket fragment hits have diverse binding modes with central pocket binding chemical groups overlaping with the space occupied by the natural substrate Wnt lipid PAM. Some fragments can show induced fit with expanded pocket volume. The screen is sensitive with the ability to pick out a substantial number of hits (11) with low affinity (IC_50_ > 1 mM). Many hits (20) show reasonable Notum inhibition potency (IC_50_ < 100 μM) with the best one having IC_50_ 0.5 μM. Structural and chemical property analysis lead us to choose six hits (**7**, **15**, **16**, **24**, **28**, and **40**) for fragment development with most delivering new analogues having better potencies. 1,2,3-Triazole **7** became the main focus of our activities and ultimately was optimized to deliver **7d** (ARUK3001185) as a potent, selective, and brain penetrant inhibitor of Notum activity suitable for use in animal models of disease.

## Experimental Section

### X-Ray Crystallographic Fragment Screen

Human Notum protein (Notum_core_ S81-T451 with a C330S mutation)^7^ was produced in HEK293S GNTI-cells and purified using standard procedures as described before.^37^ The crystals were grown in 96-well Swissci/MRC plates with reservoir solution of 1.5 M ammonium sulfate and 0.1 M sodium citrate, pH 4.2. Fragment-based screening was carried out using the XChem platform and beamline I04-1 at the Diamond Light Source (www.diamond.ac.uk/Instruments/Mx/Fragment-Screening). The crystal drops were imaged analyzed with TeXRank^44^ for ranking crystal quality and allocating drop regions for fragment dispensing so as not to damage crystals. The DSPL^32^ compounds (500 mM in DMSO) were dispensed (60 to 300 nL drop) by acoustic droplet ejection with the ECHO liquid handler (Labcyte INC).^33^ After one hour of soaking, the crystals were harvested (with no further cryoprotective reagent) with the help of a Shifter device (Oxford Lab Technologies). The X-ray diffraction data were collected on beamline I04-1, in automated unattended mode. The fragment information and data collection were managed by XChemExplorer.^27^ The data were analyzed by PanDDA^34^ and further confirmed and refined with REFMAC.^45^ All the 59 fragment hit-Notum complex structures have been validated and deposited to PDB with accession codes listed in Table 1 (all the PDB entries have been released).

### Notum Activity Assay with the OPTS Substrate

The lipase substrate OPTS has been successfully used to measure Notum enzyme activity and compound inhibitory potency. The experimental details have been described before.^36,38,39^ Briefly, the recombinant Notum protein, test compounds, and the fluorescent substrate OPTS were mixed using a Labcyte Echo 550 acoustic liquid handler. Reactions were allowed to occur for 40 min at room temperature. The endpoint fluorescence was recorded using a microplate reader (PheraSTAR FSX), and the compound IC_50_ values were calculated from curves using a 4PL fit.

### Chemistry

#### General Information

General Methods have been described in detail.^39^ Procedures for the purchase or resynthesis of the original fragment hits from the DSPL (**1**–**58**) are presented in the Supporting Information.

The purity of synthesized compounds was evaluated by NMR spectroscopy and/or liquid chromatography–mass spectroscopy (LC–MS) analysis. All compounds had purity ≥95% unless otherwise stated. Purchased compounds were used as supplied.

#### Synthesis of New Notum Inhibitors

##### (1-(4-Chloro-3-(trifluoromethyl)phenyl)-1*H*-1,2,3-triazol-4-yl)methanol (**7b**)

Step 1: sodium nitrite (420 mg, 6.14 mmol) was added portionwise to a solution of 4-chloro-3-(trifluoromethyl)aniline (1000 mg, 5.11 mmol) in TFA (5 mL) at 0 °C over 30 min. The reaction mixture was warmed to room temperature and stirred for 1.5 h. Water (0.1 mL) was added, and the mixture cooled to 0 °C. Sodium azide (365 mg, 5.62 mmol) was added portionwise over 30 min, and the mixture was then allowed to warm slowly to room temperature over 1 h. The mixture was basified to pH 8–9 by the dropwise addition of sat. aqueous NaHCO_3_ and then extracted with CH_2_Cl_2_. The combined organic extracts were dried (MgSO_4_) and evaporated under reduced pressure at ≤25 °C [Caution] to give 4-azido-1-chloro-2 (trifluoromethyl)benzene, which was used without further purification.

Step 2: propargyl alcohol (0.29 mL, 5.11 mmol), sodium l-ascorbate (405 mg, 2.05 mmol), and copper(II) sulfate pentahydrate (255 mg, 1.02 mmol) were added to a solution of the foregoing 4-azido-1-chloro-2-(trifluoromethyl)benzene (ca. 5.1 mmol) in water (10 mL) and *t-*BuOH (10 mL), and the mixture was heated at 50 °C for 2 h. The cooled mixture was diluted with water and extracted with EtOAc. The organics were washed with water (×2) and brine (×2), dried (MgSO_4_), and concentrated under reduced pressure. The residue was purified by column chromatography (0–5% MeOH in CH_2_Cl_2_) to afford **7b** (1180 mg, 4.25 mmol, 83% yield) as a white solid. LCMS (Acidic): RT 1.69 min, *m*/*z* 278.1, 280.1 [M + H]^+^; ^1^H NMR (700 MHz, DMSO*-d*_6_) δ 8.89 (s, 1H), 8.35 (d, *J* = 2.5 Hz, 1H), 8.26 (dd, *J* = 8.7, 2.6 Hz, 1H), 7.95 (d, *J* = 8.7 Hz, 1H), 5.37 (t, *J* = 5.6 Hz, 1H), 4.62 (d, *J* = 5.5 Hz, 2H); ^13^C NMR (176 MHz, DMSO*-d*_6_) δ 149.59, 135.66, 133.27, 130.09, 127.89 (q, *J* = 31.6 Hz), 125.05, 122.26 (q, *J* = 273.4 Hz), 121.42, 119.14 (q, *J* = 5.4 Hz), 54.89.

##### *N*-(4-Methyl-1*H*-benzo[*d*]imidazol-2-yl)cyanamide (**15a**)

A 10 mL thick-walled reaction vial was charged with diphenyl *N*-cyanocarbonimidate (167 mg, 0.70 mmol), 2,3-diaminotoluene (86 mg, 0.70 mmol), and 2-propanol (5 mL). The vial was sealed with a Teflon-lined crimp cap, placed in a preheated aluminum heating block at 50 °C and stirred for 3 h. The reaction was cooled to r.t. and purified by column chromatography (0–10% MeOH in CH_2_Cl_2_) to afford **15a** (40 mg, 0.23 mmol, 33% yield). LCMS (Basic): RT 1.36 min, *m*/*z* (ESI+) 173.0 [M + H]^+^; ^1^H NMR (600 MHz, DMSO*-d*_6_) δ 12.29 (app br d, *J* = 15.4 Hz, 2H), 7.03–6.98 (m, 2H), 6.93 (br d, *J* = 7.3 Hz, 1H), 2.36 (s, 3H); ^13^C NMR (151 MHz, DMSO*-d*_6_) δ 155.03, 129.99, 129.25, 123.52, 122.37, 120.50, 118.12, 107.80, 16.31.

##### 3-(4-Fluoro-3-(trifluoromethyl)phenyl)-1*H*-pyrazole (**16a**)

The compound **16a** was prepared using the two-step method used to prepare **16**, starting from 4′-chloro-3′-(trifluoromethyl)acetophenone (200 mg, 0.90 mmol). Purification by column chromatography (0–50% EtOAc in cyclohexane) gave **16a** (200 mg, 0.81 mmol, 94% yield) as an off-white solid. LC–MS (Basic): RT 1.84 min, *m*/*z* 245.1, 247.1 [M-H]; ^1^H NMR (600 MHz, CDCl_3_) δ 10.36 (br s, 1H), 8.14 (d, *J* = 1.9 Hz, 1H), 7.91 (dd, *J* = 8.4, 1.9 Hz, 1H), 7.66 (d, *J* = 2.4 Hz, 1H), 7.55 (d, *J* = 8.4 Hz, 1H), 6.67 (d, *J* = 2.4 Hz, 1H); ^13^C NMR (151 MHz, CDCl_3_) δ 149.42, 132.21, 131.88, 131.44, 130.83 (m), 129.84, 128.83 (q, *J* = 31.5 Hz), 124.91 (q, *J* = 5.5 Hz), 122.97 (q, *J* = 274 Hz), 100.07.

##### 1-(Benzo[*d*][1,3]dioxol-5-yl)-*N*-(4-chloro-3-(trifluoromethyl)benzyl)methanamine (**28a**)

A solution of 4-chloro-3-(trifluoromethyl)benzylamine (0.07 mL, 0.48 mmol) and piperonal (86 mg, 0.57 mmol) in MeOH (2 mL) was stirred at r.t for 30 min. After this time, sodium borohydride (22 mg, 0.57 mmol) was added, and the reaction mixture was stirred at r.t for a further 4 h. The reaction mixture was concentrated in vacuo, and the residue was diluted with water and extracted with EtOAc. The organic layer was washed with brine, dried (MgSO_4_), and concentrated in vacuo. The residue was purified by column chromatography (0–60% EtOAc in cyclohexane). The residue was further purified by the SCX-2 cartridge (MeOH - 1 M NH_3_ in MeOH) to afford **28** (111.6 mg, 0.32 mmol, 68% yield) as a colorless oil. Purity 87% by LCMS (Basic): RT 1.96 min, *m*/*z* 344.1 [M + H]^+^; ^1^H NMR (600 MHz, CDCl_3_) δ 7.68 (br s, 1H), 7.48–7.44 (m, 2H), 6.85 (br s, 1H), 6.78–6.75 (m, 2H), 5.95 (s, 2H), 3.80 (s, 2H), 3.71 (s, 2H); ^13^C NMR (151 MHz, CDCl_3_) δ 146.92, 146.79, 139.66, 133.84, 132.56, 131.47 (2C), 127.31, 127.28, 121.37 (2C), 108.72, 108.25, 101.09, 53.11, 51.91.

## Supporting Information Available

Schematic illustration of the overall procedure for the X-ray based fragment screen, lead identification and development. Notum inhibition SAR for additional pyrrazoles **16**, benzylamines **28** and benzimidazoles **15**. Procedures for the purchase or resynthesis of fragment hits **1**–**58**. ^1^H NMR spectra for resynthesized fragment hits **1–58**. Spectroscopic and analytical data for fragment development compounds **7b**, **15a**, **16a**, and **28a**. UHPLC analytical data for advanced lead **7d** (PDF)
Molecular formula strings and Notum IC_50_ values for **1**–**58** (CSV)

## Accession Codes

Coordinates for X-ray structures of Notum crystallized with **1** (7B7W), **2** (7B7X), **3** (7B7Y), **4** (7B84), **5** (7B86), **6** (7B87), **7** (7B89), **7a** (6ZVL), **7b** (7PKV), **7c** (7QVZ), **7d** (7PK3), **8** (6TR7), **9** (7B8A), **10** (7B8C), **11** (7B8D), **12** (7B8F), **13** (7B8G), **14** (7B8J), **15** (7B8K), **16** (7B8L), **17** (7B8M), **18** (7B8N), **19** (7B8O), **20** (7B8U), **21** (7B8X), **22** (7B8Y), **23** (7B8Z), **24** (7B98), **25** (7B99), **26** (6ZUV), **27** (7B9D), **28** (7B9I), **29** (7B9N), **30** (7B9U), **31** (7BA1), **32** (7BAC), **33** (7BAP), **34** (7BC8), **35** (7BC9), **36** (7BCC), **37** (7BCD), **38** (7 BCE), **39** (7BCF), **40** (6R8P), **41** (7BCH), **42** (7BCI), **43** (7BCK), **44** (7BCL), **45** (7BD2), **46** (7BD3), **47** (7BD4), **48** (7BD5), **49** (7BD6), **50** (7BD8), **51** (7BD9), **52** (7BDA), **53** (7BDB), **54** (7BDC), **55** (7BDD), **56** (7BDF), **57** (7BDG), **58** (7BDH) and **59** (7B01) have been deposited in the Protein Data Bank. All coordinates have been released except **7b**, **7c** and **7d** which will be released upon article publication.

## Abbreviations

ABPP: activity-based protein profiling
AD: Alzheimer’s disease
ADME: absorption distribution metabolism elimination
DMSO: dimethylsulfoxide
DSPL: Diamond-SGC Poised Library
ER: efflux ratio
FBDD: fragment-based drug design
HAC: heavy atom count
HTS: high-throughput screen
ITC: isothermal titration calorimetry
LE: ligand efficiency
LLE: lipophilic ligand efficiency
LRP5/6: low-density lipoprotein receptor-related protein 5/6
MW: molecular weight
NMR: nuclear magnetic resonance
OPTS: trisodium 8-octanoyloxypyrene-1,3,6-trisulfonate
PanDDA: Pan-Dataset Density Analysis
PDB: Protein Data Bank
PORCN: protein-serine O-palmitoleoyltransferase porcupine
ROR1/2: neurotrophic tyrosine kinase, receptor-related 1 /2
SAR: structure–activity relationship
SPR: surface plasmon resonance
TCF/LEF: T-cell factor/lymphoid enhancing factor
TPSA: topological polar surface area
TSA: thermal shift assays
Wnt: Wingless Integrated-1.
