# Supplementary material for: Structural Analysis and Development of Notum Fragment Screening Hits
Source: ACS Chem Neurosci. 2022 Jun 22;13(13):2060–77. doi: 10.1021/acschemneuro.2c00325 (PMC9264368; doi:10.1021/acschemneuro.2c00325)
Supplement: Supplementary file 1 — cn2c00325_si_001.pdf [file cn2c00325_si_001.pdf]

SUPPORTING INFORMATION

Structural Analysis and Development of Notum Fragment Screening Hits

Yuguang Zhao,[1] William Mahy,[2] Nicky J. Willis,[2] Hannah L. Woodward,[2] David Steadman,[2] Elliott D. Bayle,[2,3] Benjamin N. Atkinson,[2] James Sipthorp,[2,3] Luca Vecchia,[1] Reinis R. Ruza,[1] Karl Harlos[1], Fiona Jeganathan,[2] Stefan Constantinou,[2] Artur Costa,[2] Svend Kjær,[3] Magda Bictash,[2] Patricia C. Salinas,[4] Paul Whiting,[2] Jean-Paul Vincent,[3] Paul V. Fish,[2,3,]* E. Yvonne Jones.[1,] *

[1] Division of Structural Biology, Wellcome Centre for Human Genetics, University of Oxford, The Henry Wellcome Building for Genomic Medicine, Roosevelt Drive, Oxford, OX3 7BN, U.K.

[2] Alzheimer's Research UK UCL Drug Discovery Institute, University College London, The Cruciform Building, Gower Street, London, WC1E 6BT, U.K.

[3] The Francis Crick Institute, 1 Midland Road, Kings Cross, London NW1 1AT, U.K.

[4] Department of Cell and Developmental Biology, Laboratory for Molecular and Cellular Biology, University College London, London, WC1E 6BT, U.K.

* E-mails: yvonne@strubi.ox.ac.uk; p.fish@ucl.ac.uk

Table of contents:

Page S3 **Figure S1**. Schematic illustration of the overall procedure for the X-ray based fragment screen, lead identification and development.

Page S4 **Table S1.** Notum inhibition SAR for additional pyrrazoles **16**, benzylamines **28** and benzimidazoles **15**.

Page S6 Procedures for the purchase or resynthesis of fragment hits **1**-**58**.

Page S24 **Figure S2.** ^{1}H NMR spectra for resynthesized fragment hits **1-58.**

Page S48 **Figure S3.** Spectroscopic and analytical data for fragment development compounds **7b**, **15a**, **16a** and **28a**.

Page S56 **Figure S4.** UHPLC analytical data for advanced lead 7d.

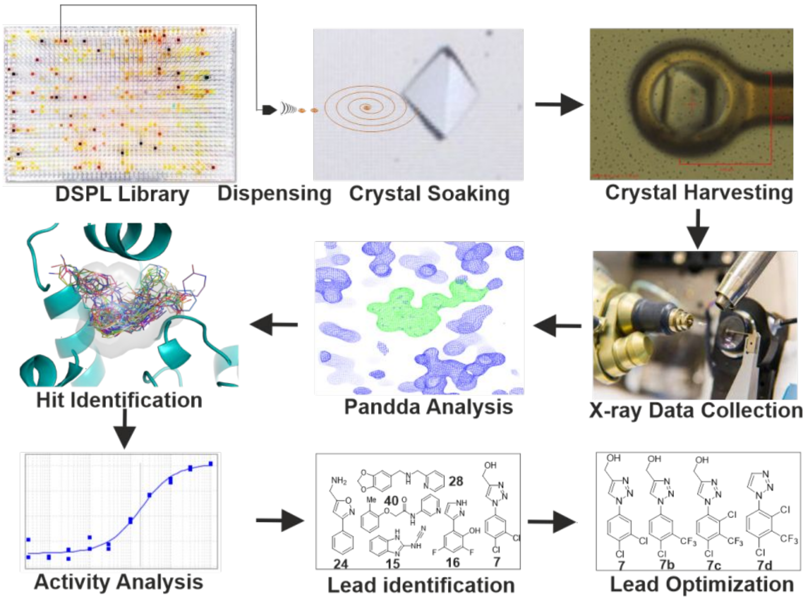

Figure S1. Schematic illustration of the overall procedure for the X-ray based fragment screen, lead identification and development.

Table S1. Notum inhibition SAR for additional pyrrazoles **16**, benzylamines **28** and benzimidazoles **15**.

Additional pyrrazoles **16b-f**, benzylamines **28b-j** and benzimidazoles **15b-m** were prepared by the general methods as described and Notum inhibition data presented here for a more complete analysis of the SARs. The most active member is in bold text.

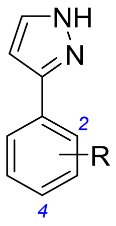

16

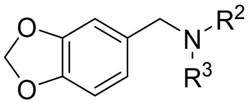

28

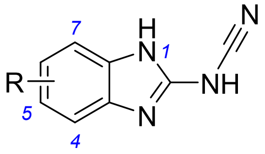

15

**Compound Number**	**R**		**Notum IC_{50} (μM)[a]**
Pyrrazoles			
**16** (Hit)	2-OH, 3-F, 5-F		3.3 ± 0.4
**17** (Hit)	2-OH, 4-OMe		34 ± 5.7
**16a**	**3-CF_3, 4-Cl**		**1.4 ± 0.1**
**16b**	H		170 ± 52
**16c**	3-F, 5-F		23 ± 6.0
**16d**	3-F, 4-F		16 ± 2.9
**16e**	3-Cl		19 ± 6.4
**16f**	3-Cl, 4-Cl		5.4 ± 1.0
Benzylamines	R^2	R^3	
**28** (Hit)	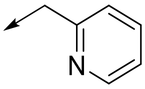	H	54 ± 6.2
**32** (Hit)	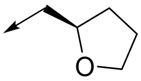	H	ca. 1000
**28a**	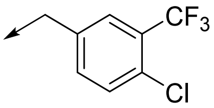	H	**16 ± 1.5**
**28b**	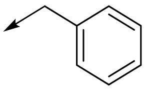	H	250 ± 53
**28c**	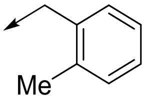	H	120 ± 28
**28d**	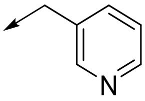	H	>1000

**28e**	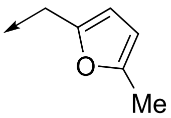	H	460 ± 53
**28f**	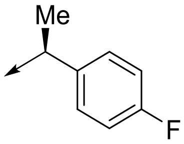	H	99 ± 13
**28g**	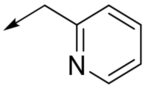	Me	710 ± 42
**28h**	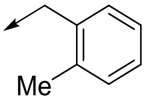	Me	560 (N=1)
**28i**	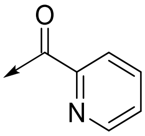	H	840 ± 50
**28j**	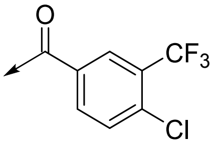	H	51 ± 0.6
Benzimidazoles			
**15** (Hit)	H		17 ± 0.9
**15a**	**4-Me**		**0.94 ± 0.06**
**15b**	1-Me		5.0 ± 0.77
**15c**	4-Cl		2.2 ± 0.31
**15d**	4-Br		2.3 ± 0.54
**15e**	4-CF_3		12 ± 1.4
**15f**	4-OMe		14 ± 1.3
**15g**	5-Me		100 ± 4.4
**15h**	5-Cl		29 ± 016
**15i**	5-Br		15 ± 3.7
**15j**	5-CF_3		36 ± 5.7
**15k**	1-Me, 4-Me		9.3 ± 3.8
**15l**	1-Me, 7-Me		2.2 ± 0.48
**15m**	4-Me, 5-Me		18 ± 5.1

[a] Values are mean ± s.d. of N = 2-4 experiments quoted to 2 s.f. unless stated otherwise. Differences of <2-fold should not be considered as significant.

Procedures for the purchase or resynthesis of fragment hits 1-58.

General Information. Unless preparative details are provided, all reagents were purchased from commercial suppliers and used without further purification. Microwave assisted reactions were performed in a Biotage Initiator+. Thin-layer chromatography (TLC) was carried out on aluminum-backed silica plates. The plates were visualized under UV (254 nm) light, followed by staining with phosphomolybdic acid dip or potassium permanganate and gentle heating. Organic solvent layers were routinely dried with anhydrous Na_2SO_4 or $MgSO_4$ and concentrated using a Büchi rotary evaporator. Compound purification by column chromatography was performed using a Biotage Isolera using prepacked Biotage SNAP KP-Sil silica cartridges or Biotage SNAP Ultra C18 reverse phase cartridges. ^{1}H and ^{13}C NMR spectra were recorded in deuterated (≥99.5%) solvents on either a Bruker Avance 300 (300 MHz), Bruker Avance 400 (400 MHz), Bruker Avance 600 (600 MHz), or Bruker Avance 700 (700 MHz). Chemical shifts (δ) are reported as parts per million (ppm), coupling constants (*J*) are reported in Hz, and signal multiplicities are reported as singlet (s), doublet (d), triplet (t), quartet (q), pentet (p), quintet (qu), sextet (sext), doublet of doublets (dd), doublet of triplets (dt), triplet of doublets (td), triplet of triplets (tt), multiplet (m), or broad singlet (br s). Liquid chromatography−mass spectrometry (LCMS) analysis was performed on a Waters Acquity H-Class UPLC system with either an acidic (HSS C18 Column, H_2O/MeCN, 0.1% TFA) or a basic (BEH C18 Column, H_2O/MeCN, 10 mM NH_4OH) mobile phase. The observed mass and isotope pattern matched the corresponding theoretical values as calculated from the expected elemental formula.

1-Ethyl-3-(5-methylisoxazol-3-yl)urea (1)

Ethylidene(oxo)ammonium (0.13 mL, 2.04 mmol) was added dropwise to a solution of 3-amino-5-methylisoxazole (200 mg, 2.04 mmol), under a nitrogen atmosphere at 0 ^{o}C, over 5 min. The reaction was then allowed to warm to room temperature and stirred for 16 h. After this time the reaction mixture was then cooled to 0 ^{o}C (external) quenched with sat. aq. Na_2CO_3 (5 mL), the organics were then separated, dried over *anhydrous* Na_2SO_4 and concentrated under reduced pressure. The crude material was recrystallised from Et_2O to give **1** (65 mg, 0.38 mmol, 19 % yield) as a white solid. LCMS (Acidic): RT 1.26 min, m/z (ESI+) 170.1 $[M+H]^+$; ^{1}H NMR (600 MHz, $CDCl_3$) δ 7.36 (brs, 1H), 7.21 (brs, 1H), 5.77 (s, 1H), 3.36 (qd, *J* = 7.3 Hz, *J* = 5.6 Hz, 2H), 1.22 (t, *J* = 7.3 Hz, 3H); ^{13}C NMR (151 MHz, $CDCl_3$) δ 169.4, 158.9, 154.3, 94.7, 35.5, 15.3, 12.5

***N*-(Benzo[*c*][1,2,5]oxadiazol-4-yl)acetamide (2)**

To a stirred solution of 2,1,3-benzoxadiazol-4-amine (100 mg, 0.74 mmol) in dichloromethane (5 mL) was added triethylamine (0.1 mL, 0.74 mmol) and the mixture stirred at 0 ^{o}C for 5 minutes. To this

bright orange solution, acetyl chloride (0.05 mL, 0.74 mmol) was then added and the reaction allowed to warm to room temperature and stirred for 3 hours. After this time further acetyl chloride (0.05 mL) was added and the reaction was left to stir overnight at room temperature under nitrogen. Product was isolated by flash silica chromatography (0-10% EtOAc in cyclohexane) to give **2** (105 mg, 0.60 mmol, 80% yield) as a yellow solid. LCMS (Acidic): RT 1.31 min, m/z (ESI+) 178.1 $[M+H]^+$; ^{1}H NMR (600 MHz, DMSO) δ 10.70 (s, 1H), 8.13 (dd, *J* = 7.2, 3.3 Hz, 1H), 7.67 (d, *J* = 9.0 Hz, 1H), 7.56 (dd, *J* = 9.0, 7.2 Hz, 1H), 2.21 (s, 3H).

***N*-(4-Methylthiazol-2-yl)propanamide (3)**

To a solution of 4-methylthiazol-2-amine (250 mg, 2.19 mmol) and triethylamine (0.46 mL, 3.28 mmol) in dichloromethane (10 mL) was added propanoyl chloride (0.21 mL, 2.41 mmol) dropwise. Upon complete addition the reaction was stirred at room temperature for 2 hours. After this time the reaction mixture was added to ice water and extracted with ethyl acetate. The organics were washed with water (x2) and brine (x2), dried over *anhydrous* $MgSO_4$, filtered and concentrated under reduced pressure. The residue was purified by column chromatography (0-100% ethyl acetate in cyclohexane) to afford **3** (191 mg, 1.12 mmol, 51% yield) as a white solid. LCMS (Acidic): RT 1.30 min, m/z (ESI+) 171.1 $[M+H]^+$; ^{1}H NMR (400 MHz, DMSO) δ 11.94 (s, 1H), 6.70 (d,*J*= 1.0 Hz, 1H), 2.45 – 2.34 (m, 2H), 2.28 – 2.19 (m, 3H), 1.07 (t,*J*= 7.5 Hz, 3H).

[4-(1*H*-1,3-Benzimidazol-1-yl-)phenyl]methanol (4)

Compound **4** was purchased from Key Organics (3T-0332).

2-[4-(1H-Pyrazol-3-yl)phenoxy]pyrimidine (5)

Compound **5** was purchased from Key Organics (8N-020).

Methyl 2-(4-aminophenoxy)benzoate (6)

Compound **6** was purchased from Santa Cruz Biotechnology (sc-269506).

(1-(3,4-Dichlorophenyl)-1*H*-1,2,3-triazol-4-yl)methanol (7)

Compound **7** was purchased from Key Organics (4F-359S).

***N*-[2-(5-Fluoro-1*H*-indol-3-yl)ethyl]acetamide (8)**

To a solution of 3-(2-aminoethyl)-5-fluoro-1H-indole (200 mg, 1.12 mmol) in dichloromethane (7 mL) was added triethylamine (0.19 mL, 1.35 mmol). The reaction mixture was cooled in an ice-water bath before the dropwise addition of acetyl chloride (0.08 mL, 1.12 mmol). The reaction was warmed to room temperature and stirred for 1 hour. After this time methanol was added and the reaction mixture was allowed to stir for a further 10 minutes. The crude reaction mixture was purified by column chromatography (0-100% ethyl acetate in cyclohexane) to afford **8** (95 mg, 0.43 mmol, 38% yield) as an off white solid. LCMS (Acidic): RT 1.40 min, m/z (ESI+) 221.2 $[M+H]^+$; ^{1}H NMR (600 MHz,

DMSO) δ 10.90 (s, 1H), 7.91 (t,*J*= 5.2 Hz, 1H), 7.32 (dd,*J*= 8.8, 4.6 Hz, 1H), 7.26 (dd,*J*= 10.1, 2.5 Hz, 1H), 7.22 (d,*J*= 2.3 Hz, 1H), 6.90 (td, *J*= 9.2, 2.5 Hz, 1H), 3.30 – 3.26 (m, 2H), 2.77 (t,*J*= 7.4 Hz, 2H), 1.80 (s, 3H).

1-Cyclohexyl-3-[2-(4-pyridyl)ethyl]urea (9)

4-(2-Aminoethyl)pyridine (0.49 mL, 4.09 mmol) was added dropwise to a solution of cyclohexyl isocyanate (0.52 mL, 4.09 mmol) in dichloromethane (10 mL) at 0 °C. The mixture was allowed to warm to room temperature and was stirred overnight. After this time the reaction mixture was quenched by addition of sat. aq. $NaHCO_3$ (10 mL). The organics were then separated, dried over *anhydrous* Na_2SO_4 and concentrated under reduced pressure. The crude material was recrystallised from Et_2O and then dried *in vacuo* to afford **9** (350 mg, 1.42 mmol, 35 % yield) as a white solid. LCMS (Acidic): RT 1.62 min, m/z (ESI+) 248.3 $[M+H]^+$; ^1H NMR (600 MHz, DMSO) δ 8.45 (dd, *J* = 4.4 Hz, *J* = 1.6 Hz, 2H), 7.21 (dd, *J* = 4.5 Hz, *J* = 1.5 Hz, 2H), 5.73 (d, *J* = 8.0 Hz, 1H), 5.69 (t, *J* = 5.7 Hz, 1H), 3.35 – 3.29 (m, 1H), 3.25 (apparent dd, *J* = 12.9 Hz, *J* = 6.9 Hz, 2H), 2.69 (t, *J* = 7.0 Hz, 2H), 1.74 – 1.67 (m, 2H), 1.65 – 1.57 (m, 2H), 1.54 – 1.46 (m, 1H), 1.28 – 1.19 (m, 2H), 1.14 – 1.01 (m, 3H); ^{13}C NMR (151 MHz, DMSO) δ 157.2, 149.4, 148.7, 124.2, 64.9, 47.7, 35.3, 33.2, 25.3, 24.5

***N*-Isobutyl-2-methyl-3,8*a*-dihydroimidazo[1,2-*a*]pyridine-3-carboxamide (10)**

To a stirred solution of 2-methylimidazo[1,2-*a*]pyridine-3-carboxylic acid (125 mg, 0.71 mmol) in dichloromethane (25 mL) was added *N,N*-diisopropylethylamine (0.15mL, 0.85 mmol) and *N*-(3-dimethylaminopropyl)-*N'*-ethylcarbodiimide hydrochloride (163 mg, 0.85 mmol). This was stirred for 5 minutes at room temperature and the mixture went from an off-white suspension to a clear solution. Isobutylamine (0.08 mL, 0.85 mmol) was then added and the reaction left to stir overnight at room temperature. The product was purified by flash silica chromatography (50-100% ethyl acetate in cyclohexane) to give **10** (68 mg, 0.29 mmol, 41% yield) as a pink oil. LCMS (Acidic): RT 1.13 min, m/z (ESI+) 232.3 $[M+H]^+$; ^1H NMR (400 MHz, DMSO) δ 8.94 (dt, J = 7.0, 1.1 Hz, 1H), 7.86 (s, 1H), 7.55 (dt, J = 9.0, 1.1 Hz, 1H), 7.35 (ddd, J = 9.0, 7.0, 1.1 Hz, 1H), 6.99 (td, J = 7.0, 1.1 Hz, 1H), 3.15 (dd, J = 6.7, 6.0 Hz, 2H), 2.57 (s, 3H), 1.97 – 1.74 (m, 1H), 0.93 (d, J = 6.7 Hz, 6H).

4-Amino-N-(2-pyridin-2-yl)benzenesulfonamide (11)

Compound **11** was purchased from Sigma Aldrich.

4-(Benzimidazol-1-ylmethyl)benzonitrile (12) A solution of benzimidazole (300 mg, 2.54 mmol) in *N,N*-dimethylformamide (10 mL) was cooled in an ice-water bath for 5 minutes before the addition of sodium hydride 60% wt. on mineral oil (111 mg, 2.79 mmol). The reaction was stirred for a further 5 minutes before 4-(bromomethyl)benzonitrile (547 mg, 2.79 mmol) was added. The reaction was warmed to room temperature and stirred overnight. After this time the reaction mixture was added

to ice water and extracted with ethyl acetate. The organics were washed with water (x2) and brine (x2), dried over *anhydrous* $MgSO_4$, filtered and concentrated under reduced pressure. The residue was purified by column chromatography (0-100% ethyl acetate in cyclohexane) to afford **12** (470 mg, 2.01 mmol, 79% yield) as a white solid. LCMS (Acidic): RT 1.23 min, m/z (ESI+) 234.2 $[M+H]^+$; ^1H NMR (400 MHz, DMSO) δ 8.43 (s, 1H), 7.81 (d,*J*= 8.3 Hz, 2H), 7.71 – 7.60 (m, 1H), 7.54 – 7.38 (m, 3H), 7.27 – 7.12 (m, 2H), 5.63 (s, 2H).

2-(Benzyloxy)aniline (13)

Compound **13** was purchased from Sigma Aldrich (189049).

2-[(1,1'-Biphenyl)-4-yl]acetic acid (14)

Compound **14** was purchased from Santa Cruz Biotechnology (sc-256684).

1*H*-Benzimidazol-2-ylcyanamide (15)

A solution of diphenoxymethylenecyanamide (330 mg, 1.39 mmol) and *o*-phenylenediamine (150 mg, 1.39 mmol) in 2-propanol (5 mL) was heated to 50 °C for 2 hours. After this time the reaction mixture was cooled to room temperature and concentrated *in vacuo*. The crude material was suspended in dichloromethane and adsorbed on to silica. The residue was purified by column chromatography (0-15% methanol in dichloromethane) to afford **15** (164 mg, 1.04 mmol, 75% yield) as a white solid. LCMS (Acidic): RT 1.00 min, m/z (ESI+) 159.2 $[M+H]^+$; ^1H NMR (400 MHz, DMSO) δ 12.28 (s, 1H), 7.17 (dt,*J*= 6.7, 3.6 Hz, 1H), 7.12 (dt,*J*= 5.4, 3.5 Hz, 1H).

2,4-Difluoro-6-(1*H*-pyrazol-3-yl)phenol (16)

A solution of 3',5'-difluoro-2'-hydroxyacetophenone (500 mg, 2.9 mmol) and *N*,*N*-dimethylformamide dimethyl acetal (1.2 mL, 8.71 mmol) in *N*,*N*-dimethylformamide (10 mL) (anhydrous) was purged with N_2 and then heated to 90°C for 2 hours. The reaction mixture was cooled to r.t and concentrated *in vacuo*. The crude brown solid (*E*)-1-(3,5-difluoro-2-hydroxy-phenyl)-3-(dimethylamino)prop-2-en-1-one was dried under high vacuum for 2 hours before being used directly in the next reaction.

To a solution of (*E*)-1-(3,5-difluoro-2-hydroxy-phenyl)-3-(dimethylamino)prop-2-en-1-one (650 mg, 2.86 mmol) in methyl alcohol (20 mL) was added hydrazine monohydrate (1.4 mL, 28.6 mmol), and then heated to 68°C for 18 hrs. The reaction mixture was concentrated *in vacuo* and the residue purified by column chromatography (0-50 % EtOAc in cyclohexane). The product was crystallised from CH_2Cl_2-cyclohexane and dried under vacuum to afford **16** (233 mg, 1.19 mmol, 41.5% yield) as an off-white solid. LCMS (Basic): RT 1.32 min, m/z (ESI+) 197.1 $[M+H]^+$; ^1H NMR (600 MHz, $CDCl_3$) δ 10.85 (br s, 1H), 10.37 (br s, 1H), 7.70 (d, *J* = 2.6 Hz, 1H), 7.10 (ddd, *J* = 9.2, 3.0, 1.9 Hz, 1H), 6.84 (ddd, *J* = 10.7, 8.0, 3.0 Hz, 1H), 6.69 (d, J = 2.6 Hz, 1H); ^{13}C NMR (151 MHz, $CDCl_3$) δ 155.71 (d, *J* = 11.5 Hz), 154.13

(d, *J* = 11.5 Hz), 152.38 (d, *J* = 12.8 Hz), 150.75 (m), 140.90 (dd, *J* = 12.8, 3.5 Hz), 129.60, 118.36 (dd, *J* = 9.6, 4.4 Hz), 107.62 (dd, *J* = 24.0, 3.5 Hz), 104.26 (dd, *J* = 26.8, 22.4 Hz), 102.76.

5-Methoxy-2-(1*H*-pyrazol-3-yl)phenol (17)

Prepared by the 2-step method used to prepare **16**, starting from 2'-hydroxy-4'-methoxyacetophenone (500 mg, 3.0 mmol). Purification by column chromatography (0-20 % EtOAc in cyclohexane) gave **17** (390 mg, 2.1 mmol, 69% yield) as a white crystalline solid. LCMS (Acidic): RT 1.48 min, m/z 191.2 $[M+H]^+$; ^{1}H NMR (600 MHz, DMSO-d_6) d 11.15 (s, 1H), 7.88 (br. s., 1H), 7.63 (d, *J* = 8.21 Hz, 1H), 6.77 (br. s., 1H), 6.40 - 6.56 (m, 2H), 3.74 (s, 3H); ^{13}C NMR (151 MHz, DMSO-d_6) d 159.9, 156.6, 150.3, 129.8, 127.6, 110.4, 105.8, 101.3, 101.2, 55.1.

5-Amino-2-((pyridine-3-ylmethyl)amino)benzoic acid (18)

Compound **18** was purchased from Santa Cruz Biotech (sc-318110).

[(2*S*)-2-Methyl-1-piperidyl]-morpholino-methanone (19)

To a stirred solution of *N,N*-diisopropylethylamine (0.13 mL, 0.74 mmol) in dichloromethane (5 mL) was added (S)-( )-2-methylpiperidine (0.24 mL, 2.02 mmol). The mixture was stirred at 0 °C for 5 minutes. To the resulting orange solution, 4-morpholinecarbonyl chloride (0.09 mL, 0.74 mmol) was then added and the reaction allowed to warm to room temperature and stirred overnight. After this time the reaction mixture was diluted with sat aq. $NaHCO_3$ (10 mL) and dichloromethane (10 mL). The organics were then separated, dried over *anhydrous* Na_2SO_4 and concentrated under reduced pressure. The residue was purified by reverse phase column chromatography (0-100% acetonitrile in water, 10 mM NH_4OH modifier) to afford **19** (120 mg, 0.57 mmol, 28% yield) as an off-white solid. LCMS (Acidic): RT 1.47 min, m/z (ESI+) 213.3 $[M+H]^+$; ^{1}H NMR (600 MHz, DMSO) δ 3.92 – 3.86 (m, 1H), 3.55 (t, *J* = 4.8 Hz, 4H), 3.30 – 3.27 (m, 1H), 3.11 – 3.01 (m, 4H), 2.96 – 2.88 (m, 1H), 1.62 – 1.39 (m, 5H), 1.38 – 1.29 (m, 1H), 1.11 (d, *J* = 6.9 Hz, 3H); ^{13}C NMR (151 MHz, DMSO) δ 163.41, 65.93, 48.18, 47.19, 41.49, 29.91, 25.31, 18.85, 15.45.

4-(1-Piperidyl)-1,2,5-oxadiazol-3-amine (20)

To a 100 mL round-bottomed flask fitted with a condenser was added 3,4-diaminofurazan (200 mg, 2 mmol) and cesium carbonate (1965 mg, 6 mmol). The system was flushed with N_2 and 1,5-dibromopentane (0.27 mL, 2 mmol) was added. The heterogeneous reaction mixture was heated at reflux for 20 hours. After this time the reaction mixture was cooled to room temperature and filtered. The filtrate was concentrated *in vacuo*, and purified by reverse phase column chromatography (0-100% acetonitrile in water, 10 mM NH_4OH modifier) to afford **20** (68 mg, 0.40 mmol, 20% yield) as an off white solid. LCMS (Acidic): RT 1.44 min, m/z (ESI+) 169.1 $[M+H]^+$; ^{1}H NMR

(600 MHz, $CDCl_3$) d 3.93 (br. s., 2H), 3.12 - 3.32 (m, 4H), 1.61 - 1.73 (m, 6H); ^{13}C NMR (151 MHz, $CDCl_3$) d 154.2, 150.4, 50.0, 25.4, 24.0

_N_-Methyl-_N_-[(5-methyl-2-furyl)methyl]-1-phenyl-methanamine (21)

A solution of 5-methylfurfural (0.14 mL, 1.43 mmol) and *N*-benzylmethylamine (0.16 mL, 1.24 mmol) in methanol (2 mL) was stirred at room temperature for 20 mins. After this time sodium borohydride (52 mg, 1.36 mmol) was added. The reaction mixture was stirred at room temperature for 18 hours under N_2. The reaction mixture was concentrated *in vacuo*. The residue was diluted with water and extracted with ethyl acetate. The organic layer was washed with brine, dried over *anhydrous* $MgSO_4$ and concentrated *in vacuo.* The residue was purified by column chromatography (0-20 % ethyl acetate in cyclohexane) to afford **21** (100 mg, 0.46 mmol, 38% yield) as a colourless oil. LCMS (Basic): RT 1.89 min, m/z (ESI+) 216.3 $[M+H]^+$; ^{1}H NMR (600 MHz, $CDCl_3$) δ 7.35-7.31 (m, 4H), 7.26 (m, 1H), 6.08 (d, *J* = 2.9 Hz, 1H), 5.90 (m, 1H), 3.54 (s, 2H), 3.53 (m, 2H), 2.30 (s, 3H), 2.24 (s, 3H); ^{13}C NMR (151 MHz, $CDCl_3$) δ 151.85, 150.56, 138.78, 129.32 (2C), 128.34 (2C), 127.11, 109.58, 106.00.

5-(3-Ethoxyphenyl)-1,3-4-thiadiazol-2-amine (22)

Phosphorus(V) oxychloride (770 mg) was added to a stirring mixture of 3-ethoxybenzoic acid (830 mg, 5.0 mmol) and thiosemicarbazide (460 mg, 5.0 mmol) at RT, and then heated at 75 °C for 0.5 h. The mixture was cooled to 0 °C, water (5 mL) was cautiously added dropwise, and then heated at reflux for 4 h. After cooling to RT, the mixture was basified to pH 8 by the dropwise addition of aqueous 50% NaOH solution under stirring. The resulting precipitate was collected by filtration, recrystallized from EtOH, and dried *in vacuo* to give **22** (75 mg, 0.34 mmol, 7 % yield) as an off-white solid. LCMS (Basic): RT 1.42 min, *m/z* (ESI$^+$) 222.2 $[C_{10}H_{11}N_3OS+H]^+$; ^{1}H NMR (600 MHz, DMSO) δ 7.44 (s, 2H), 7.38 – 7.34 (m, 1H), 7.29 – 7.25 (m, 2H), 7.01 – 6.97 (m, 1H), 4.08 (q, $^3J_{HH}$ = 7.0 Hz, 2H), 1.34 (t, $^3J_{HH}$ = 7.0 Hz, 3H); ^{13}C NMR (151 MHz, DMSO) δ 168.6, 158.9, 156.2, 132.3, 130.4, 118.9, 115.9, 111.3, 63.2, 14.6.

2-(4-(3-Chlorophenyl)piperazin-1-yl)acetonitrile (23)

STEP 1: A suspension of iodocopper (160 mg, 0.84 mmol) and 1-(2-hydroxy-1-naphthyl)naphthalen-2-ol (rac-BINAP) (180 mg, 0.63 mmol) in *N,N*-dimethylformamide (2 mL) was stirred at room temperature for 10 minutes. After this time 1-chloro-3-iodo-benzene (0.52 mL, 4.19 mmol), *tert*-butyl piperazine-1-carboxylate (1172 mg, 6.29 mmol) and potassium phosphate tribasic (1780 mg, 8.39 mmol) was added. The reaction was heated to 100 °C overnight. After this time the reaction was cooled to room temperature, diluted with ethyl acetate and filtered through a sintered glass funnel. The filtrate was washed with brine (x2), water (x1) and again brine (x1). The organics were separated, dried over *anhydrous* $MgSO_4$, filtered and adsorbed on to silica. The residue was purified by column chromatography (0-20% ethyl acetate in cyclohexane) to afford *tert*-butyl 4-(3-

chlorophenyl)piperazine-1-carboxylate (763 mg, 2.57 mmol, 61% yield) as a pale yellow solid. LCMS (Acidic): RT 1.97 min, m/z (ESI+) 297.1 $[M+H]^+$; ^{1}H NMR (600 MHz, DMSO) δ 7.22 (t, J = 8.1 Hz, 1H), 6.96 (t, J = 2.2 Hz, 1H), 6.93 – 6.88 (m, 1H), 6.81 (ddd, J = 7.8, 1.9, 0.6 Hz, 1H), 3.47 – 3.39 (m, 4H), 3.17 – 3.10 (m, 4H), 1.42 (s, 9H).

STEP 2: To a solution of ***tert***-butyl 4-(3-chlorophenyl)piperazine-1-carboxylate (763 mg, 2.57 mmol) in dichloromethane (30 mL) was added trifluoroacetic acid (3.9 mL, 51.4 mmol). The reaction was stirred at room temperature for 3 hours. After this time the reaction mixture was concentrated *in vacuo* and azeotroped with dichloromethane (x2). The crude material was quenched with sat. aq. $NaHCO_3$ solution, diluted with water and extracted with dichloromethane (x2). The organics were combined and washed with water (x1) and brine (x2). The organics were dried over *anhydrous* $MgSO_4$, filtered and concentrated. The resulting oil was diluted in ethyl acetate and 4N HCl in 1,4-dioxane was added dropwise resulting in precipitation. The precipitate was filter *via* buchner filtration and washed with Et_2O. The solid was dried under a continuous flow of air to afford 1-(3-chlorophenyl)piperazine dihydrochloride (320 mg, 1.19 mmol, 46% yield) as a white solid. LCMS (Acidic): RT 1.23 min, m/z (ESI+) 197.2 $[M+H]^+$, >95%; ^{1}H NMR (600 MHz, DMSO) δ 9.45 (s, 2H), 7.25 (t, J = 8.1 Hz, 1H), 7.03 (t, J = 2.2 Hz, 1H), 6.94 (dd, J = 8.2, 2.2 Hz, 1H), 6.88 – 6.84 (m, 1H), 3.47 – 3.39 (m, 4H), 3.20 – 3.11 (m, 4H).

STEP 3: A suspension of 1-(3-chlorophenyl)piperazine dihydrochloride (320 mg, 1.19 mmol) and potassium carbonate (574 mg, 4.15 mmol) in acetonitrile (4.75 mL) was charged with 2-chloroacetonitrile (0.08 mL, 1.31 mmol) and heated to 80 ^oC for 4 hours. After this time the reaction mixture was cooled to room temperature and diluted with ethyl acetate. The crude mixture was filtered through a sintered glass funnel and concentrated under reduced pressure. The residue was purified by column chromatography (0-50% ethyl acetate in cyclohexane) to afford **23** (156 mg, 0.66 mmol, 56% yield) as a white solid. LCMS (Acidic): RT 1.69 min, m/z (ESI+) 236.2 $[M+H]^+$; ^{1}H NMR (400 MHz, DMSO) δ 7.21 (t, J = 8.1 Hz, 1H), 6.96 (t, J = 2.1 Hz, 1H), 6.91 (dd, J = 8.4, 1.9 Hz, 1H), 6.85 – 6.74 (m, 1H), 3.79 (s, 2H), 3.27 – 3.15 (m, 4H), 2.58 (dd, J = 14.1, 9.1 Hz, 4H).

3-(Phenylisoxazol-5-yl)methanamine (24)

Compound **24** was purchased from Maybridge.

***N*-(4-Anilinophenyl)acetamide (25)**

1-Methyl-2-pyrrolidinone (3 mL) was added to nitrobenzene (0.2 mL, 2 mmol), *N*-(4-oxocyclohexyl)acetamide (621 mg, 4 mmol) and 2-dicyclohexylphosphino-2,4,6-triisopropylbiphenyl (95 mg, 0.2 mmol) under argon. The mixture was degassed with argon for 15 min before addition of palladium acetate (22 mg, 0.1 mmol). The mixture was then further degassed for a further 5 minutes before heating to 150 ^oC for 16 h. After this time the reaction was cooled to room temperature,

concentrated *in vacuo* and the residue was purified by column chromatography (1:1 ethyl acetate in cyclohexane) to afford **25** (98 mg, 0.43 mmol, 22 % yield) as a white solid. LCMS (Basic): RT 1.54 min, m/z (ESI+) 227.2 $[M+H]^+$; ^{1}H NMR (600 MHz, DMSO) δ 9.76 (s, 1H), 8.01 (s, 1H), 7.49 – 7.40 (m, 2H), 7.21 – 7.15 (m, 2H), 7.05 – 6.94 (m, 4H), 6.75 (apparent t, *J* = 7.3 Hz, 1H), 2.00 (s, 3H); ^{13}C NMR (151 MHz, DMSO) δ 167.7, 144.2, 138.6, 132.4, 129.2, 120.3, 118.9, 117.9, 115.7, 23.9

[1-(4-Chlorophenyl)-1*H*-1,2,3-triazol-4-yl]methanol (26)

Compound **26** was purchased from Key Organics (4F-329S).

(4-Acetamidophenoxy)acetic acid (27)

Compound **27** was purchased from Key Organics (SS-3896).

1-(Benzo[*d*][1,3]dioxol-5-yl)-*N*-(pyridine-2-ylmethyl)methanamine (28)

Compound **28** was purchased from Santa Cruz Biotech (sc-326192).

Benzyl *N*-hydroxycarbamate (29)

Compound **29** was purchased from Key Organics (MS-2251).

4-(2-Furylmethyl)-1,4-thiazinane 1,1-dioxide hydrochloride (30)

A solution of furan-2-carbaldehyde (0.26 mL, 3.12 mmol), sodium triacetoxyborohydride (992 mg, 4.68 mmol) and thiomorpholine 1,1-dioxide (633 mg, 4.68 mmol) in tetrahydrofuran (12 mL) was stirred at room temperature for 2 days. After this time the reaction mixture was basified using 2N NaOH aq. solution to pH 11. The reaction mixture was diluted with water and extracted with dichloromethane. The organics were separated, dried over *anhydrous* $MgSO_4$, filtered and concentrated under reduced pressure. The resulting oil was dissolved in ethyl acetate and acidified with 4N HCl in 1,4-dioxane. The precipitate was filtered *via* buchner filtration and dried under a continuous flow of air to afford **30.**HCl (229 mg, 0.91 mmol, 29% yield) as a white solid. LCMS (Acidic): RT 0.58 min, m/z (ESI+) 216.1 $[M+H]^+$; ^{1}H NMR (400 MHz, DMSO) δ 7.87 – 7.67 (m, 1H), 6.71 (d,*J*= 2.9 Hz, 1H), 6.56 (dd,*J*= 3.2, 1.9 Hz, 1H), 4.46 (s, 2H), 3.73 – 3.43 (m, 8H).

2-(4-Ethylphenoxy)-1-(1-piperidyl)ethenone (31)

N,N-Diisopropylethylamine (0.39 mL, 2.25 mmol) was added to a solution of *O*-(1H-benzotriazol-1-yl)-*N,N,N',N'*-tetramethyluronium hexafluorophosphate (356 mg, 0.94 mmol) and 4-ethylphenoxyacetic acid (169 mg, 0.94 mmol) in *N*,*N*-dimethylformamide (3 mL) at 0 °C. The mixture was then allowed to warm to room temperature before addition of piperidine (0.07 mL, 0.75 mmol). The reaction mixture was stirred at room temperature for 16 hours. After this time the reaction was diluted with ethyl acetate (25 mL) and washed sequentially with 1.0 M LiCl (10 mL) and sat. aq. $NaHCO_3$ (10 mL). The combined organics were dried over *anhydrous* $MgSO_4$, filtered and concentrated under reduced pressure. The residue was purified by column chromatography (0-100% ethyl acetate in cyclohexane)

to afford **31** (115 mg, 0.46 mmol, 62% yield) as a clear colourless oil. LCMS (Acidic): RT 1.74 min, m/z (ESI+) 248.2 $[M+H]^+$; ^{1}H NMR (600 MHz, $CDCl_3$) δ 7.11 (d, *J* = 8.6 Hz, 2H), 6.90 – 6.85 (m, 2H), 4.65 (s, 2H), 3.59 – 3.45 (m, 4H), 2.59 (q, *J* = 7.6 Hz, 2H), 1.69 – 1.49 (m, 6H), 1.20 (t, *J* = 7.6 Hz, 3H)

(*R*)-1-(Benzo[*d*][1,3]dioxol-5-yl)-*N*-((tetrahydrofuran-2-yl)methyl)methanamine (32)

Compound **32** was purchased from Santa Cruz Biotech (sc-326178).

***N*^{1}-(Pyridin-2-yl)benzene-1,2-diamine (33)**

o-Phenylenediamine (540 mg, 4.99 mmol) and 2-chloropyridine (0.52 mL, 5.49 mmol) were heated to 145 ^{o}C for 16 hours. After this time the reaction mixture was cooled to RT, diluted with water (3 mL) and acetone (3 mL). The solution was adjusted to pH 10, with 2M Na_2CO_3 and then diluted with ethyl acetate (50 mL). The organics were then separated, dried over *anhydrous* Na_2SO_4, filtered and concentrated under reduced pressure. The residue was purified by column chromatography (1:3 ethyl acetate in cyclohexane) to afford **33** (28 mg, 0.15 mmol, 3% yield) as white solid. LCMS (Acidic): RT 0.37 min, m/z (ESI+) 186.2 $[M+H]^+$; ^{1}H NMR (600 MHz, DMSO) δ 8.07 – 7.99 (m, 1H), 7.86 (s, 1H), 7.45 (ddd, *J* = 8.7, *J* = 7.1, *J* = 2.0 Hz, 1H), 7.23 (dd, *J* = 7.8, *J* 1.3 Hz, 1H), 6.86 (td, *J* = 7.8, *J* 1.5 Hz, 1H), 6.73 (dd, *J* = 7.9, *J* 1.4 Hz, 1H), 6.62 (ddd, *J* = 7.1 Hz, *J* = 5.0, *J* = 0.9 Hz, 1H), 6.57 – 6.52 (m, 2H), 4.81 (s, 2H); ^{13}C NMR (151 MHz, DMSO) δ 157.38, 147.64, 142.49, 137.20, 125.93, 124.82, 124.75, 116.35, 115.48, 113.29, 108.33

2-(Benzyloxy)benzohydrazide (34)

Compound **34** was purchased from Santa Cruz Biotech (sc-305895).

2-Methoxy-*N*-(4-phenylthiazol-2-yl)acetamide (35)

N,N-Diisopropylethylamine (0.39 mL, 2.25 mmol) was added to a solution of methoxyacetic acid (68 mg, 0.75 mmol) and O-(1H-benzotriazol-1-yl)-N,N,N',N'-tetramethyluronium hexafluorophosphate (355 mg, 0.94 mmol) in *N,N*-dimethylformamide (3 mL) at 0 ^{o}C. The mixture was then allowed to warm to room temperature before addition of 2-Amino-4-phenylthiazole (165 mg, 0.94 mmol). The reaction mixture was stirred at room temperature for 16 hours. After this time the reaction mixture was then diluted with ethyl acetate (25 mL) and washed sequentially with 1.0 M LiCl (10 mL) and sat. aq. $NaHCO_3$ (10 mL). The combined organics were dried over *anhydrous* $MgSO_4$, filtered and concentrated under reduced pressure. The residue was purified by reverse phase column chromatography (0-100% acetonitrile in water, 10 mM NH_4OH modifier) to afford **35** (67 mg, 0.27 mmol, 36% yield) as an off-white solid. LCMS (Basic): RT 1.63 min, m/z (ESI+) 249.2 $[M+H]^+$; ^{1}H NMR (600 MHz, DMSO) δ 12.17 (s, 1H), 7.90 (dd, *J* = 8.2 Hz, *J* = 1.1 Hz, 2H), 7.64 (s, 1H), 7.46 – 7.40 (m, 2H),

7.34 – 7.31 (m, 1H), 4.17 (s, 2H), 3.37 (s, 3H); ^{13}C NMR (151 MHz, DMSO) δ 168.62, 157.36, 148.86, 134.25, 128.78, 127.86, 125.70, 108.23, 70.48, 58.79.

4-(4-Pyrrol-1-ylphenyl)morpholine (36)

A mixture of 1-(4-iodophenyl)pyrrole (500 mg, 1.86 mmol), morpholine (0.17 mL, 1.86 mmol), iodocopper (70 mg, 0.37 mmol), and potassium carbonate (770 mg, 5.57 mmol) in *anhydrous* dimethylsulfoxide (4 mL) was purged with N_2 and heated to 110 °C for 16 hours. The solution was cooled to room temperature and diluted with ethyl acetate. The organics were washed with water and brine, dried over *anhydrous* $MgSO_4$, and adsorbed on to silica. The residue was purified by column chromatography (0-100% ethyl acetate in cyclohexane) to afford **36** (30 mg, 0.13 mmol, 7% yield) as a white solid. LCMS (Acidic): RT 1.71 min, m/z (ESI+) 229.2 $[M+H]^+$; ^{1}H NMR (600 MHz, DMSO) δ 7.47 – 7.33 (m, 2H), 7.22 (t, *J* = 2.2 Hz, 2H), 7.07 – 6.94 (m, 2H), 6.20 (t, *J* = 2.2 Hz, 2H), 3.80 – 3.68 (m, 4H), 3.18 – 3.03 (m, 4H).

2-(Morpholinomethyl)naphthalen-1-ol hydrochloride (37)

To a rapidly stirring solution of 1-hydroxy-2-naphthaldehyde (200 mg, 1.16 mmol), morpholine (0.13 mL, 1.39 mmol) in tetrahydrofuran (10 mL) was added sodium triacetoxyborohydride (295 mg, 1.39 mmol) portion wise. After complete addition the reaction was stirred at room temperature for 2 hours. After this time the reaction was diluted with water and basified using NaOH 1N solution. The organics were extracted with dichloromethane three times. The organics were combined, dried over *anhydrous* $MgSO_4$, and adsorbed on to silica. The residue was purified by column chromatography (0-20% methanol 2N NH_3 in dichloromethane). The free base was dissolved in a minimal volume of ethyl acetate and charged with 0.4 mL of 7N HCl in 1,4-dioxane. The resulting precipitate was removed *via* filtration and dried under a continuous flow of air to afford **37.**HCl (162 mg, 0.58 mmol, 50% yield) as a white solid. LCMS (Acidic): RT 1.21 min, m/z (ESI+) 244.2 $[M+H]^+$; ^{1}H NMR (600 MHz, DMSO) δ 10.87 (s, 1H), 10.18 (s, 1H), 8.34 (d,*J*= 8.2 Hz, 1H), 7.88 (d,*J*= 7.5 Hz, 1H), 7.60 (d,*J*= 8.4 Hz, 1H), 7.55 (dtd,*J*= 16.4, 6.8, 1.3 Hz, 2H), 7.48 (d,*J*= 8.4 Hz, 1H), 4.54 (s, 2H), 3.93 (d,*J*= 12.0 Hz, 2H), 3.77 (t,*J*= 11.9 Hz, 2H), 3.32 (d,*J*= 12.1 Hz, 2H), 3.19 (d,*J*= 8.4 Hz, 2H).

3-((4-Methylpiperidin-1-yl)methyl)-1*H*-indole (38)

To a solution of indole-3-carboxaldehyde (250 mg, 1.72 mmol) in methanol (10 mL) was added 4-methylpiperidine (0.24 mL, 2.07 mmol) and the reaction was heated to 60 °C for 2 hours under nitrogen. After 2 hours, the reaction was allowed to cool to room temperature and sodium borohydride (130 mg, 3.44 mmol) was added portion-wise and the reaction left to stir at room temperature for 1 hour. After this time, the reaction was concentrated and the residue was washed with water and extracted with ethyl acetate. Organics were washed with brine, dried over anhydrous

$MgSO_4$ and filtered. Product was then purified by flash silica chromatography (0-20% methanol in dichloromethane) to afford **38** (41 mg, 0.2 mmol, 10% yield) as an off-white solid. LCMS (Acidic): RT 1.25 min, m/z (ESI+) 229.3 $[M+H]^+$; ^1H NMR (600 MHz, MeOD) δ 7.59 (d, *J* = 7.9 Hz, 1H), 7.35 (d, *J* = 7.9 Hz, 1H), 7.21 (s, 1H), 7.13 – 7.06 (m, 1H), 7.06 – 6.98 (m, 1H), 4.93 (s, 3H), 3.76 (s, 2H), 2.98 (d, *J* = 11.8 Hz, 2H), 2.21 – 2.02 (m, 2H), 1.60 (d, *J* = 13.3 Hz, 2H), 1.38 – 1.27 (m, 1H), 1.22 (ddd, *J* = 15.5, 12.5, 3.7 Hz, 2H), 0.88 (d, *J* = 6.5 Hz, 3H); ^{13}C NMR (150 MHz, MeOD) δ 137.8, 129.6, 126.7, 122.6, 120.1, 119.7, 112.4, 110.1, 54.4, 53.8, 34.7, 31.5, 22.1

2-(4-(2,5-Dioxopyrrolidin-1-yl)phenoxy)acetic acid (39)

STEP 1: 4-Aminophenol (542 mg, 4.97 mmol) and succinic anhydride (497 mg, 4.97 mmol) were added to acetic acid (7 mL). The reaction was heated to 100 ^oC for 24 hours. After this time the reaction mixture was cooled to room temperature and diluted with water (10 mL). The resulting precipitate was collected, washed with water (5 mL) and cyclohexane (20 mL). The solid was dried at 45 ^oC under vacuum overnight to afford 1-(4-hydroxyphenyl)pyrrolidine-2,5-dione (667 mg, 3.49 mmol, 70% yield) as a white solid. The intermediate was taken on to the next step without further purification. LCMS (Acidic): RT 0.36 min, m/z (ESI+) 190.1 $[M-H]^-$; ^1H NMR (400 MHz, DMSO) δ ^1H NMR (600 MHz, DMSO) δ 9.67 (s, 1H), 7.05 – 6.99 (m, 2H), 6.86 – 6.79 (m, 2H), 2.74 (s, 4H); ^{13}C NMR (151 MHz, DMSO) δ 177.1, 157.1, 128.2, 123.7, 115.2, 28.3.

STEP 2: 1-(4-Hydroxyphenyl)pyrrolidine-2,5-dione (330 mg, 1.73 mmol) was dissolved in acetonitrile (8 mL) . *tert*-Butyl bromoacetate (0.38 mL, 2.59 mmol) and potassium carbonate (716 mg, 5.18 mmol) were added. The reaction mixture was heated to 70 ^oC for 18 hours. After this time the reaction mixture was cooled to room temperature, diluted with water (20 mL) and ethyl acetate (50 mL). The organics were separated and the aqueous was washed with ethyl acetate (2 x 50 mL). The combined organics were washed with brine (30 mL), separated, dried over *anhydrous* $MgSO_4$, filtered and the crude material was purified by flash silica chromatography (0-100% ethyl acetate in cyclohexane) to afford *tert*-butyl 2-(4-(2,5-dioxopyrrolidin-1-yl)phenoxy)acetate (458 mg, 1.50 mmol , 87% yield) as a white solid. LCMS (Basic): RT 1.66 min, m/z (ESI+) 304.2 $[M-H]^-$; ^1H NMR (600 MHz, $CDCl_3$) δ 7.24 – 7.17 (m, 2H), 7.01 – 6.94 (m, 2H), 4.51 (s, 2H), 2.87 (s, 4H), 1.49 (s, 9H); ^{13}C NMR (151 MHz, $CDCl_3$) δ 176.4, 167.7, 157.9, 127.8, 125.5, 115.4, 82.7, 66.0, 28.4, 28.1

STEP 3: *tert*-Butyl 2-(4-(2,5-dioxopyrrolidin-1-yl)phenoxy)acetate (150 mg, 0.50 mmol) was suspended in anhydrous toluene (7 mL) under N_2 and heated to 100 ^oC until all the solid dissolved. To this *p*-toluenesulfonic acid monohydrate (10 mg, 0.05 mmol) was added and the reaction was stirred at 100 ^oC for 1 hour. The reaction mixture was allowed to cool to room temperature, sonicated for 1 minute and then filtered. The solid was washed with toluene (5 mL), followed by water (5 mL) and

Et_2O (10 mL) to afford **39** (108 mg, 0.43 mmol, 88% yield) as a white solid. LCMS (Acidic): RT 0.66 min, m/z (ESI+) 250.1 $[M+H]^+$; ^1H NMR (600 MHz, DMSO) δ 13.04 (s, 1H), 7.20 – 7.12 (m, 2H), 7.04 – 6.93 (m, 2H), 4.72 (s, 2H), 2.76 (s, 4H); ^{13}C NMR (151 MHz, DMSO) δ 177.1, 170.1, 157.3, 128.30, 125.8, 114.6, 64.6, 28.4.

2-(2-Methylphenoxy)-N-(3-pyridyl)acetamide (40)

N,N-Diisopropylethylamine (0.39 mL, 2.25 mmol) was added to a solution of O-(1H-benzotriazol-1-yl)-N,N,N',N'-tetramethyluronium hexafluorophosphate (355 mg, 0.94 mmol) and (2-Methylphenoxy)acetic acid (125 mg, 0.75 mmol) in *N*,*N*-dimethylformamide (3 mL) at 0 °C. The mixture was then allowed to warm to room temperature before addition of 3-aminopyridine (71 mg, 0.75 mmol). The reaction mixture was stirred at room temperature for 16 hours. After this time the reaction was diluted with ethyl acetate (25 mL) and washed sequentially with 1.0 M LiCl (10 mL) and sat. aq. $NaHCO_3$ (10 mL). The combined organics were dried over *anhydrous* $MgSO_4$, filtered and concentrated under reduced pressure. The residue was purified by reverse phase column chromatography (0-100% acetonitrile in water, 10 mM NH_4OH modifier) to afford **40** (79 mg, 0.326 mmol, 59% yield) as an off-white solid. LCMS (Acidic): RT 1.59 min, m/z (ESI+) 243.3 $[M+H]^+$; ^1H NMR (600 MHz, DMSO) δ 10.26 (s, 1H), 8.78 (d, *J* = 2.4 Hz, 1H), 8.29 (dd, *J* = 4.7, *J* = 1.4 Hz, 1H), 8.06 (ddd, *J* = 8.3 Hz, *J* = 2.3 Hz, *J* = 1.6 Hz, 1H), 7.37 (dd, *J* = 8.3 Hz, *J* = 4.7 Hz, 1H), 7.20 – 7.11 (m, 2H), 6.88 (dd, *J* = 7.5 Hz, *J* = 5.8 Hz, 2H), 4.75 (s, 2H), 2.26 (s, 3H); ^{13}C NMR (151 MHz, DMSO) δ 167.4, 156.0, 144.6, 141.3, 135.1, 130.6, 126.9, 126.7, 126.2, 123.6, 121.0, 111.6, 67.3, 16.1

***N*-Benzyl-2-methoxy-acetamide (41)**

N,N-Diisopropylethylamine (0.39 mL, 2.25 mmol) was added to a solution of methoxyacetic acid (0.06 mL, 0.75 mmol) and O-(1H-benzotriazol-1-yl)-N,N,N',N'-tetramethyluronium hexafluorophosphate (356 mg, 0.94 mmol) in *N*,*N*-dimethylformamide (3 mL) at 0 °C. The mixture was then stirred for 15 min before addition of benzylamine (0.1 mL, 0.94 mmol) and the mixture was warmed to room temperature and stirred for 16 hours. After this time the reaction mixture was diluted with ethyl acetate (25 mL) and washed sequentially with 1.0 M LiCl (10 mL) and sat. aq. $NaHCO_3$ (10 mL). The combined organics were dried over anhydrous $MgSO_4$, filtered and concentrated under reduced pressure. The residue was purified by reverse phase column chromatography (0-100% acetonitrile in water, 10 mM NH_4OH) to afford **41** (79 mg, 0.44 mmol, 59% yield) as a white solid; LCMS (Basic): RT 1.35 min, m/z (ESI+) 180.2 $[M+H]^+$; ^1H NMR (600 MHz, DMSO) δ 8.34 – 8.3 (m, 1H), 7.34 – 7.28 (m, 2H), 7.26 – 7.21 (m, 3H), 4.29 (d, *J* = 6.3 Hz, 2H), 3.85 (s, 2H), 3.32 (s, 3H); ^{13}C NMR (151 MHz, DMSO) δ 169.0, 139.6, 128.3, 127.3, 126.7, 71.5, 58.7, 41.6.

***N*-(2-Hydroxy-4-methylphenyl)propanamide (42)**

To a vigorously stirred biphasic mixture of 2-amino-5-methylphenol (100 mg, 0.81 mmol) and saturated aq. solution $NaHCO_3$ (2 mL, 0.81 mmol) in dichloromethane (2 mL) was added propanoyl chloride (0.08 mL, 0.89 mmol). The reaction mixture stirred at room temperature for 2 hours. After this time the reaction was diluted with dichloromethane and washed with water (x1) and brine (x1). The organics were then separated, dried over *anhydrous* $MgSO_4$, filtered and adsorbed on to silica. The residue was purified by column chromatography (0-100% ethyl acetate in cyclohexane) to afford **42** (125 mg, 0.70 mmol, 86% yield) as an off-white solid. LCMS (Acidic): RT 1.40 min, m/z (ESI+) 180.1 $[M+H]^+$; ^{1}H NMR (400 MHz, DMSO) δ 9.62 (s, 1H), 9.19 (s, 1H), 7.48 (d,*J*= 8.1 Hz, 1H), 6.66 (d,*J*= 1.3 Hz, 1H), 6.56 (dd,*J*= 8.1, 1.3 Hz, 1H), 2.43 – 2.30 (m, 2H), 2.19 (s, 3H), 1.07 (t,*J*= 7.6 Hz, 3H).

(2*S*)-*N*-[(4-Methoxyphenyl)methyl]tetrahydrofuran-2-carboxamide (43)

N,N-Diisopropylethylamine (0.39 mL, 2.25 mmol) was added to a solution of (S)-(-)-tetrahydro-2-furoic acid (0.07 mL, 0.75 mmol) and O-(1H-benzotriazol-1-yl)-N,N,N',N'-tetramethyluronium hexafluorophosphate (356 mg, 0.94 mmol) in *N*,*N*-dimethylformamide (3 mL) at 0 °C. The mixture was then allowed to warm to room temperature before addition of 4-methoxybenzylamine (103 mg, 0.75 mmol). The reaction mixture was stirred at room temperature for 16 hours. After this time the reaction was diluted with ethyl acetate (25 mL) and washed sequentially with 1.0 M LiCl (10 mL) and sat. aq. $NaHCO_3$ (10 mL). The combined organics were dried over *anhydrous* $MgSO_4$, filtered and concentrated under reduced pressure. The residue was purified by reverse phase column chromatography (0-100% acetonitrile in water, 10 mM NH_4OH modifier) to afford **43** (160 mg, 0.68 mmol, 62% yield) as a clear colourless oil. LCMS (Acidic): RT 1.40 min, m/z (ESI+) 236.2 $[M+H]^+$; ^{1}H NMR (600 MHz, $CDCl_3$) δ 7.21 – 7.18 (m, 2H), 6.91 (s, 1H), 6.88 – 6.84 (m, 2H), 4.42 – 4.34 (m, 3H), 3.93 – 3.82 (m, 2H), 3.80 (s, 3H), 2.31 (ddd, *J* = 15.4, 12.8, 7.9 Hz, 1H), 2.13 – 2.05 (m, 1H), 1.95 – 1.81 (m, 2H); ^{13}C NMR (151 MHz, $CDCl_3$) δ 173.15, 159.13, 130.34, 129.20, 114.20, 78.63, 69.54, 55.44, 42.50, 30.40, 25.68.

Methyl 2-(4-cyanophenoxy)acetate (44)

To a vigorously stirred solution of 4-hydroxybenzonitrile (500 mg, 4.2 mmol) and potassium carbonate (870 mg, 6.3 mmol) in acetonitrile (11 mL) was added methyl 2-bromoacetate (0.44 mL, 4.62 mmol) dropwise. Following complete addition the reaction was heated under microwave conditions to 100 °C for 30 minutes. After this time the reaction mixture was added to ice water and extracted with ethyl acetate. The organics were washed with water (x2) and brine (x2), dried over *anhydrous* $MgSO_4$, filtered and concentrated under reduced pressure. The residue was purified by column chromatography (0-100% ethyl acetate in cyclohexane) to afford **44** (569 mg, 2.98 mmol, 71% yield)

as a white solid. LCMS (Acidic): RT 1.47 min, m/z (ESI+) 190.1 $[M-H]^-$; ^1H NMR (400 MHz, DMSO) δ 7.86 – 7.70 (m, 2H), 7.18 – 7.04 (m, 2H), 4.94 (s, 1H), 3.70 (s, 3H).

_N_-[2-(4-Hydroxyphenyl)ethyl]pyridine-2-carboxamide (45)

N,N-Diisopropylethylamine (0.39 mL, 2.25 mmol) was added to a solution of O-(1H-benzotriazol-1-yl)-N,N,N',N'-tetramethyluronium hexafluorophosphate (356 mg, 0.94 mmol) and picolinic acid (92 mg, 0.75 mmol) in *N*,*N*-dimethylformamide (3 mL) at 0 °C. The mixture was then allowed to warm to room temperature before addition of tyramine (129 mg, 0.94 mmol) and the reaction was stirred at room temperature for 16 hours. After this time the reaction mixture was then diluted with ethyl acetate (25 mL) and washed sequentially with 1.0 M LiCl (10 mL) and sat. aq. $NaHCO_3$ (10 mL). The combined organics were dried over *anhydrous* $MgSO_4$, filtered and concentrated under reduced pressure The residue was purified by column chromatography (0-10% methanol in dichloromethane) to afford **45** (71 mg, 0.29 mmol, 39% yield) as an off-white solid. LCMS (Acidic): RT 1.39 min, m/z (ESI+) 243.2 $[M+H]^+$; ^1H NMR (600 MHz, $CDCl_3$) δ 8.53 – 8.51 (m, 1H), 8.19 (d, *J* = 7.8 Hz, 1H), 8.12 (s, 1H), 7.84 (apparent td, *J* = 7.7 Hz, *J* = 1.7 Hz, 1H), 7.41 (ddd, *J* = 7.5, *J* = 4.8, *J* = 1.1 Hz, 1H), 7.12 (d, *J* = 8.4 Hz, 2H), 6.81 – 6.77 (m, 2H), 4.89 (s, 1H), 3.69 (apparent dd, *J* = 13.8 Hz, *J* = 6.8 Hz, 2H), 2.87 (t, *J* = 7.2 Hz, 2H).

4-Methoxy-6-phenyl-pyrimidin-2-amine (46)

A solution of 2-amino-6-phenylpyrimidin-4-ol (400 mg, 2.14 mmol) in *N*,*N*-dimethylformamide (6 mL) was cooled to 0 °C and for 0.5 hours before sodium hydride (94 mg, 2.35 mmol) was added. The mixture was stirred for 15 min, before iodomethane (0.13 mL, 2.14 mmol) was then slowly added and stirred for a further 1 hour. After this time the mixture was warmed to room temperature and quenched by pouring onto sat. aq. $NaHCO_3$ (10 mL). The reaction mixture was diluted with dichloromethane (10 mL) and the organics were then separated, dried over *anhydrous* Na_2SO_4, filtered and concentrated under reduced pressure. The residue was purified by reverse phase column chromatography (0-100% acetonitrile in water, 10 mM NH_4OH modifier) to afford **46** (36 mg, 0.18 mmol, 8% yield) as a white solid. LCMS (Acidic): RT 1.15 min, m/z (ESI+) 202.1 $[M+H]^+$; ^1H NMR (600 MHz, DMSO) δ 7.98 – 7.93 (m, 1H), 7.46 – 7.40 (m, 1H), 7.23 (s, 1H), 6.20 (s, 1H), 3.29 (s, 1H); ^{13}C NMR (151 MHz, DMSO) δ 162.48, 160.28, 156.15, 137.19, 129.93, 128.41, 126.55, 96.01, 27.72.

1-(4-Methoxyphenyl)-3-(pyridin-4-yl)urea (47)

To a stirred solution of 4-aminopyridine (100.0 mg, 1.06 mmol) in toluene (10 mL) was added 4-methoxyphenyl isocyanate (0.17 mL, 1.28 mmol) and the reaction heated at reflux under nitrogen for 3 hours. After this time, solvent was removed under reduced pressure to give a white solid. This was washed with diethyl ether and filtered. The crude material was purified by flash silica chromatography

(0-20% methanol in dichloromethane) to afford **47**(218 mg, 0.89 mmol, 84% yield) as a white solid. LCMS (Acidic): RT 1.17 min, m/z (ESI+) 244.2 $[M+H]^+$; ^1H NMR (400 MHz, MeOD) δ 8.31 (dd, *J* = 5.0, 1.5 Hz, 2H), 7.50 (dd, *J* = 5.0, 1.5 Hz, 2H), 7.33 (d, *J* = 9.1 Hz, 2H), 6.89 (d, *J* = 9.1 Hz, 2H), 3.78 (s, 3H).

2-(4-Ethoxyphenyl)acetic acid (48)

Compound **48** was purchased from Alfa Aesar.

1-Methyl-5-(phenylamino)-1*H*-pyrazol-3-ol (49)

Compound **49** was purchased from Fluorochem (478474).

2,5-Dimethylphenyl isonicotinate (50)

Isonicotinoyl chloride hydrochloride (364 mg, 2.05 mmol) was added to a solution of 2,5-dimethylphenol (100 mg, 0.82 mmol) in pyridine (2 mL) and stirred at room temperature for three hours. After this time, water (10 mL) was added and the mixture was stirred for thirty minutes. The organics were extracted with ethyl acetate, washed with brine, dried over *anhydrous* $MgSO_4$, filtered and concentrated. The crude material was purified by flash silica chromatography (0-20% ethyl acetate in cyclohexane) to afford **50** (186 mg, 2.05 mmol, 100% yield) as a white solid. LCMS (Acidic): RT 1.70 min, m/z (ESI+) 228.2 $[M+H]^+$; ^1H NMR (600 MHz, $CDCl_3$) δ 8.96 – 8.69 (s, 2H), 8.14 – 7.84 (s, 2H), 7.16 (d, *J* = 7.7 Hz, 1H), 7.01 (d, *J* = 7.7 Hz, 1H), 6.96 (s, 1H), 2.33 (s, 3H), 2.17 (s, 3H).

(4-Chloro-2-methyl-pyrazol-3-yl)-(1-piperidyl)methanone (51)

A solution of 4-chloro-2-methyl-1*H*-pyrazole-5-carboxylic acid (50 mg, 0.31 mmol) and *N*-[(dimethylamino)-1*H*-1,2,3-triazolo-[4,5-*b*]pyridin-1-ylmethylene]-*N*-methylmethanaminium hexafluorophosphate *N*-oxide (HATU) (118 mg, 0.31 mmol) in *N,N*-dimethylformamide (3 mL) and cooled in an ice-water bath for 5 minutes. After this time triethylamine (0.04 mL, 0.31 mmol) was added and the reaction was stirred for a further 10 minutes in an ice-water bath before piperidine (0.03 mL, 0.31 mmol) was added. The reaction mixture was allowed to warm to room temperature and stir for 1 hour at room temperature. After this time the reaction mixture was adsorbed on to silica and the residue was purified by column chromatography (0-80% ethyl acetate in cyclohexane) to afford **51** (56 mg, 0.25 mmol, 79% yield) as a white solid. LCMS (Acidic): RT 1.51 min, m/z (ESI+) 228.1 $[M+H]^+$; ^1H NMR (400 MHz, DMSO) δ 7.61 (s, 1H), 3.76 (s, 3H), 3.61 (s, 2H), 3.27 (t,*J*= 5.5 Hz, 2H), 1.78 – 1.39 (m, 6H).

2-(Benzyloxy)acetic acid (52)

Compound **52** was purchased from Acros.

5-Ethyl-3-methyl-*N*-(5-methylisoxazol-3-yl)isoxazole-4-carboxamide (53)

N,N-Diisopropylethylamine (0.39 mL, 2.25 mmol) was added to a solution of O-(1H-benzotriazol-1-yl)-N,N,N',N'-tetramethyluronium hexafluorophosphate (356 mg, 0.94 mmol) and 3-ethyl-5-methylisoxazole-4-carboxylic acid (116 mg, 0.75 mmol) in *N*,*N*-dimethylformamide (3 mL) at 0 °C. The mixture was then allowed to warm to room temperature before addition of 3-amino-5-methylisoxazole (92 mg, 0.94 mmol). The reaction mixture was stirred at room temperature for 16 hours. After this time the reaction was diluted with ethyl acetate (25 mL) and washed sequentially with 1.0 M LiCl (10 mL) and sat. aq. $NaHCO_3$ (10 mL). The combined organics were dried over *anhydrous* $MgSO_4$, filtered and concentrated under reduced pressure. The residue was purified by column chromatography (0-10% methanol in dichloromethane) to afford **53** (115 mg, 0.49 mmol, 65% yield) as a pale yellow solid. LCMS (Acidic): RT 1.50 min, m/z (ESI+) 236.3 $[M+H]^+$; ^1H NMR (600 MHz, DMSO) δ 11.09 (s, 1H), 6.68 (d, J = 0.7 Hz, 1H), 2.75 (q, J = 7.5 Hz, 2H), 2.53 (s, 3H), 2.40 (d, J = 0.7 Hz, 3H), 1.16 (t, J = 7.5 Hz, 3H).

1-Methyl-*N*-(*o*-tolylmethyl)tetrazol-5-amine (54)

STEP 1: To a solution of o-tolylmethanamine (0.5 mL, 4 mmol) in dichloromethane (20 mL) cooled in an ice-water bath, was added a solution of triethylamine (0.84 mL, 6 mmol) and o-tolylmethanamine (0.5 mL, 4 mmol) in dichloromethane (20 mL) drop wise. After complete addition the reaction was warmed to room temperature and stirred for 2 hours. After this time the reaction mixture was added to water and extracted with dichloromethane (x3). The organics were combined, washed with brine (x1), dried with over *anhydrous* $MgSO_4$, filtered and adsorbed on to silica. The residue was purified by column chromatography (0-50% ethyl acetate in cyclohexane) to afford 2-chloro-*N*-(*o*-tolylmethyl)acetamide (575 mg, 2.91 mmol, 73% yield) as a white solid. LCMS (Acidic): RT 1.55 min, m/z (ESI+) 196.1 $[M+H]^+$; ^1H NMR (600 MHz, DMSO) δ 8.60 (s, 1H), 7.24 – 7.12 (m, 4H), 4.28 (d,*J*= 5.7 Hz, 2H), 4.12 (s, 2H), 2.27 (s, 3H).

STEP 2: To a solution of 1-methyl-2,5-dihydro-1H-1,2,3,4-tetrazole-5-thione (352 mg, 3.04 mmol) in methanol (6 mL) was added potassium hydroxide (200 mg, 3.04 mmol). The reaction was stirred at room temperature for 30 minutes before 2-chloro-N-(o-tolylmethyl)acetamide (400 mg, 2.02 mmol) was added. The reaction was warmed to 30 °C and stirred overnight. After this time the reaction mixture was diluted with water and acidified with 1N HCl aq. solution. The aqueous layer was extracted with ethyl acetate three times and the organics were combined, dried over *anhydrous* $MgSO_4$, filtered and adsorbed on to silica. The residue was purified by column chromatography (0-30% ethyl acetate in cyclohexane) to afford 2-(1-methyltetrazol-5-yl)sulfanyl-*N*-(*o*-tolylmethyl)acetamide (356 mg, 1.28 mmol, 63% yield) as a white solid. LCMS (Acidic): RT 1.51 min, m/z (ESI+) 278.1 $[M+H]^+$; ^1H NMR (600 MHz, CDCl3) δ 7.23 (s, 1H), 7.21 – 7.12 (m, 4H), 4.43 (d, J = 5.6 Hz, 2H), 3.96 (s, 2H), 3.94 (s, 3H), 2.28 (s, 3H).

STEP 3: To a solution of sodium hydroxide (29 mg, 0.72 mmol) in ethanol (8 mL) was added 2-(1-methyltetrazol-5-yl)sulfanyl-N-(o-tolylmethyl)acetamide (200 mg, 0.72 mmol). The reaction mixture was heated to 80 °C overnight. After this time the reaction was cooled to room temperature and diluted with water and neutralised with 1N HCl solution. The aqueous layer was extracted with dichloromethane three times. The organics were combined and dried over *anhydrous* $MgSO_4$, filtered and adsorbed on to silica. The residue was purified by column chromatography (0-10% methanol in dichloromethane). Further purification *via* reverse phase column chromatography (5-100% water in acetonitrile, 0.1% formic acid modifier) afforded **54** (30 mg, 0.15 mmol, 21% yield) as a white solid. LCMS (Acidic): RT 1.42 min, m/z (ESI+) 204.2 $[M+H]^+$; ^{1}H NMR (600 MHz, $CDCl_3$) δ 7.33 (d, J = 7.5 Hz, 1H), 7.29 – 7.24 (m, 1H), 7.22 (dd, J = 13.2, 6.8 Hz, 2H), 4.63 (d, J = 5.4 Hz, 2H), 4.12 (s, 1H), 3.75 (s, 3H), 2.38 (s, 3H).

(*R*)-2-([1,1'-Biphenyl]-4-yloxy)propanoic acid (55)

Compound **55** was purchased from Santa Cruz Biotech (sc-334586).

4-(2-Phenoxyacetyl)piperazin-2-one (56)

N,N-Diisopropylethylamine (0.39 mL, 2.25 mmol) was added to a solution of phenoxyacetic acid (114 mg, 0.75 mmol) and *O*-(1*H*-benzotriazol-1-yl)-*N,N,N',N'*-tetramethyluronium hexafluorophosphate (356 mg, 0.94 mmol) at 0 °C and stirred for 15 min, before addition of piperazine-2-one (75 mg, 0.75 mmol). The mixture was warmed to room temperature, stirred for 16 hours, then cautiously concentrated to an oil at 30 °C. The residue was purified by reverse phase column chromatography (0-100% acetonitrile in water, 0.1% formic acid modifier) to afford **56** (19 mg, 0.08 mmol, 11% yield) as a clear colourless oil. LCMS (Acidic): RT 1.20 min, m/z (ESI+) 235.1 $[M+H]^+$; ^{1}H NMR (600 MHz, MeOD) δ 7.29 – 7.24 (m, 2H), 6.98 – 6.93 (m, 3H), 4.82 – 4.79 (m, 2H), 4.22 (br. s, 1H), 4.14 (br. s, 1H), 3.79 – 3.74 (m, 2H), 3.40 – 3.36 (m, 1H), 3.31 – 3.28 (m, 1H).

***N*-(1-Ethylbenzimidazol-2-yl)acetamide (57)**

To a solution of 1-ethyl-1H-benzoimidazol-2-ylamine (100 mg, 0.62 mmol) and triethylamine (0.1 mL, 0.68 mmol) in dichloromethane (5 mL) was added acetyl chloride (0.05 mL, 0.68 mmol) dropwise with vigorous stirring. The reaction mixture was stirred at room temperature for 1 hour. After this time the reaction mixture was adsorbed directly on to silica and the residue was purified by column chromatography (0-100% ethyl acetate in cyclohexane) to afford **57** (98 mg, 0.48 mmol, 78% yield) as a pale brown solid. LCMS (Acidic): RT 0.53 min, m/z (ESI+) 204.2 $[M+H]^+$; ^{1}H NMR (400 MHz, DMSO) δ 10.45 (s, 1H), 7.51 (s, 2H), 7.20 (dqd, *J*= 14.8, 7.4, 1.3 Hz, 2H), 4.11 (q,*J*= 7.1 Hz, 2H), 2.30 – 1.95 (m, 3H), 1.40 – 1.14 (m, 3H).

***N*-(2,1,3-benzothiadiazol-5-yl)acetamide (58)**

To a stirred solution of 2,1,3-benzothiadiazol-5-amine (224 mg, 1.48 mmol) in dichloromethane (5 mL) was added *N,N*-Diisopropylethylamine (0.52 mL, 2.96 mmol) at 0 °C for 5 minutes. To the resulting orange solution was added acetyl chloride (0.11 mL, 1.48 mmol), and the reaction was warmed to room temperature and stirred overnight. After this time the mixture was diluted with dichloromethane (10 mL) and washed with 0.1 M HCl (3 x 10 mL). The residue was purified by column chromatography (0-10% methanol in dichloromethane) to afford **58** (32 mg, 0.17 mmol, 11% yield) as a pale yellow solid. LCMS (Acidic): RT 1.32 min, m/z (ESI+) 194.1 $[M+H]^+$; ^{1}H NMR (400 MHz, DMSO) δ 10.41 (s, 1H), 8.54 (d, *J* = 2.0 Hz, 1H), 8.02 (dd, *J* = 9.4, *J* 0.6 Hz, 1H), 7.68 (dd, *J* = 9.4 Hz, *J* = 2.0 Hz, 1H), 2.14 (s, 3H).

Figure S2. **^{1}H NMR spectra for resynthesized fragment hits 1-58.**

1-Ethyl-3-(5-methylisoxazol-3-yl)urea (1)

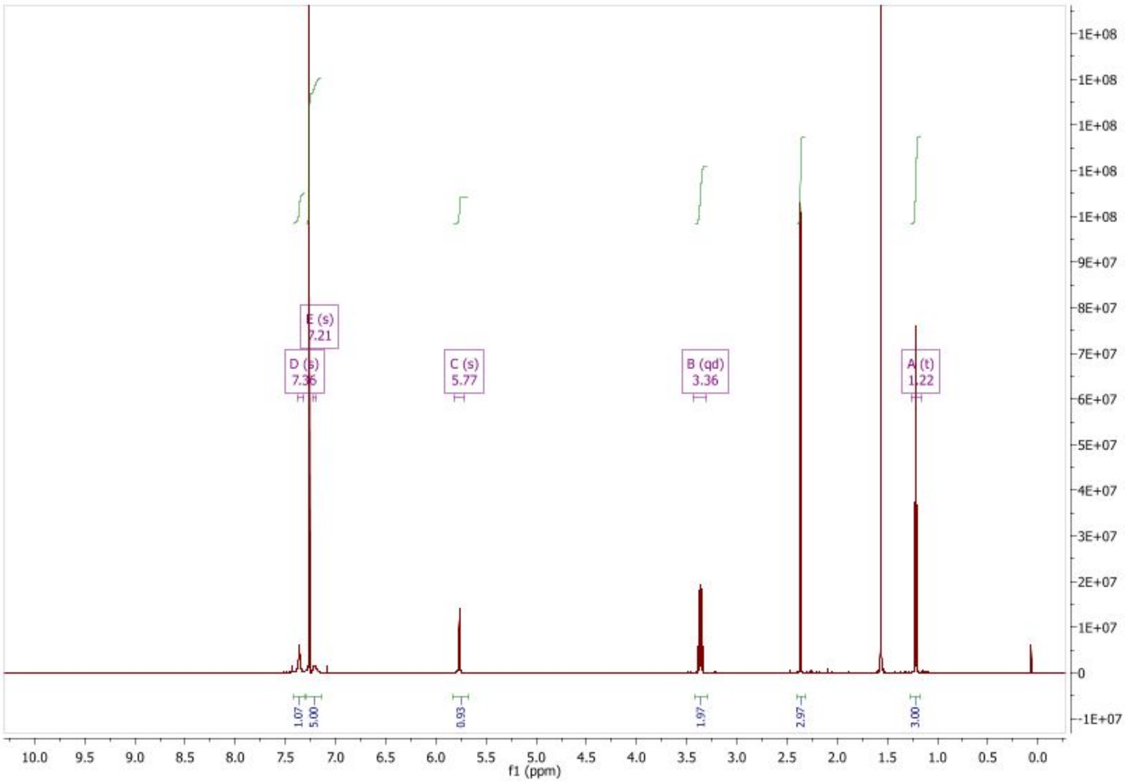

***N*-(Benzo[*c*][1,2,5]oxadiazol-4-yl)acetamide (2)**

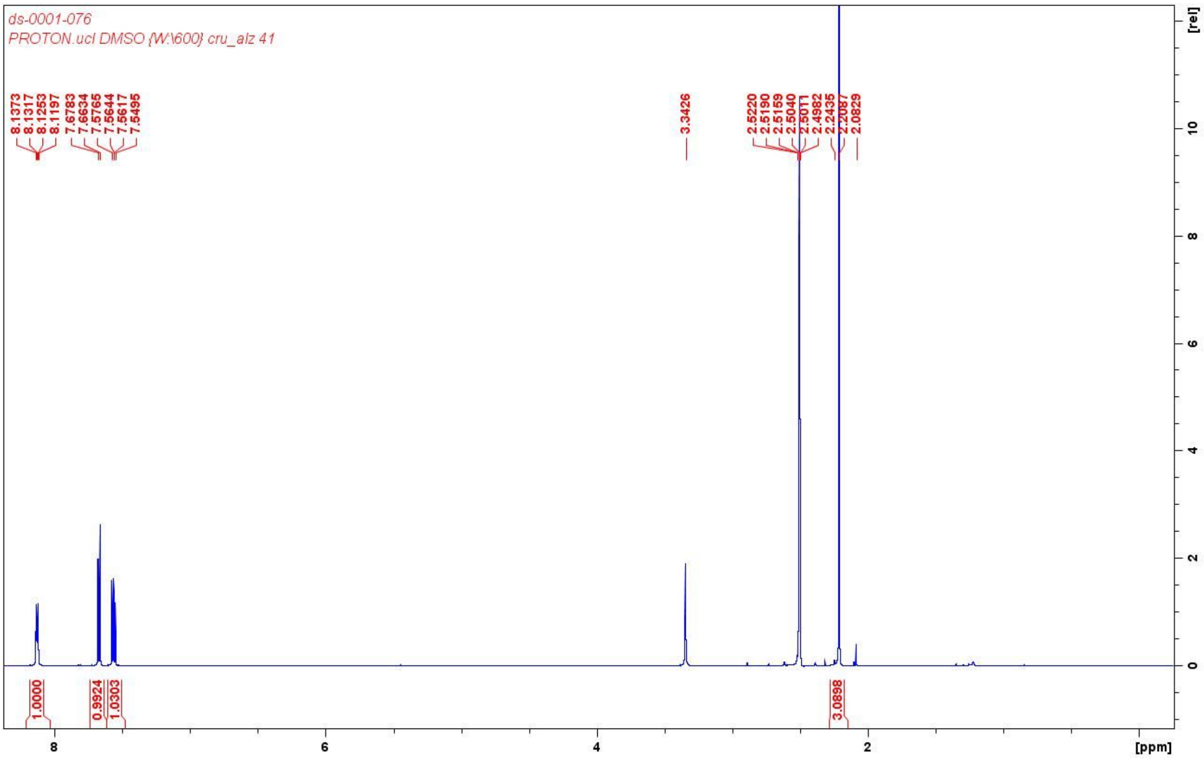

***N*-(4-Methylthiazol-2-yl)propanamide (3)**

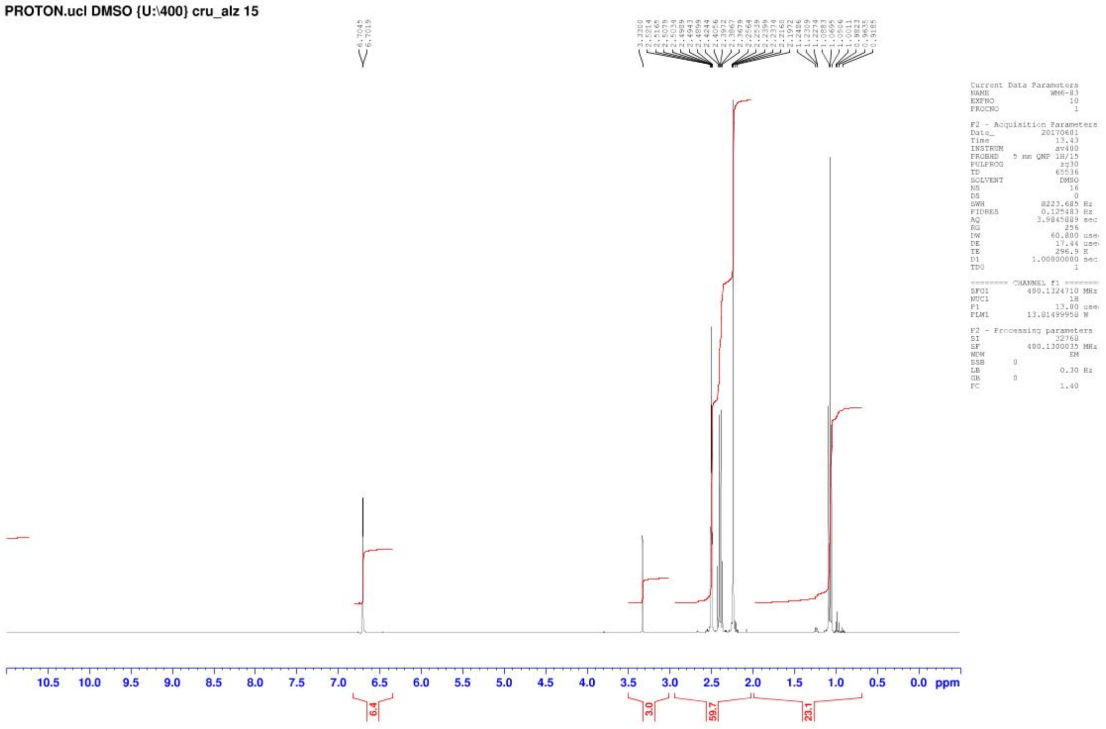

[4-(1*H*-1,3-Benzimidazol-1-yl-)phenyl]methanol (4)

Compound **4** was purchased from Key Organics (3T-0332).

2-[4-(1H-Pyrazol-3-yl)phenoxy]pyrimidine (5)

Compound **5** was purchased from Key Organics (8N-020).

Methyl 2-(4-aminophenoxy)benzoate (6)

Compound **6** was purchased from Santa Cruz Biotechnology (sc-269506).

(1-(3,4-Dichlorophenyl)-1*H*-1,2,3-triazol-4-yl)methanol (7)

Compound **7** was purchased from Key Organics (4F-359S).

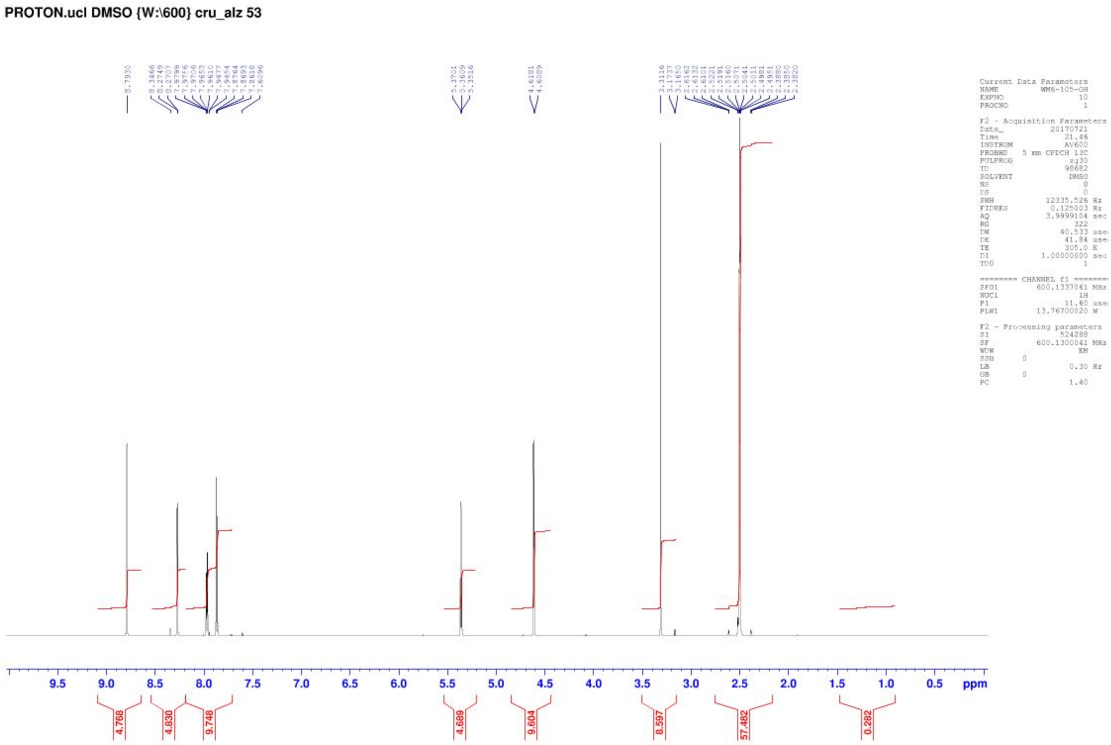

***N*-[2-(5-Fluoro-1*H*-indol-3-yl)ethyl]acetamide (8)**

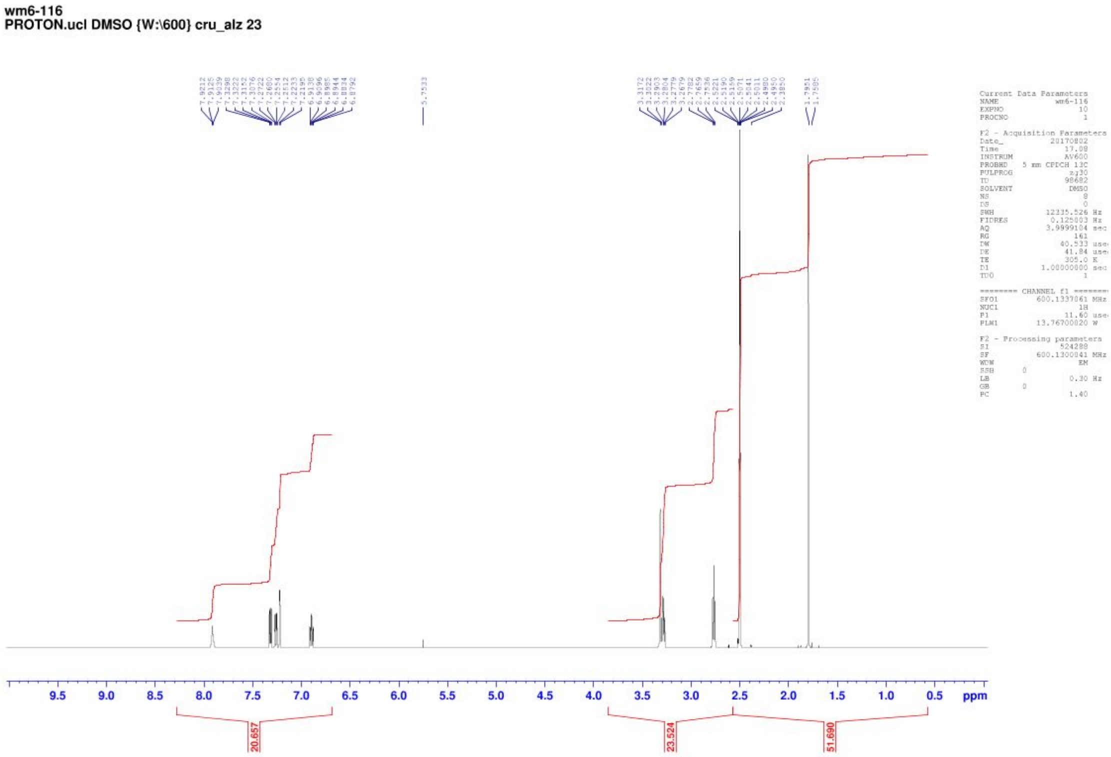

1-Cyclohexyl-3-[2-(4-pyridyl)ethyl]urea (9)

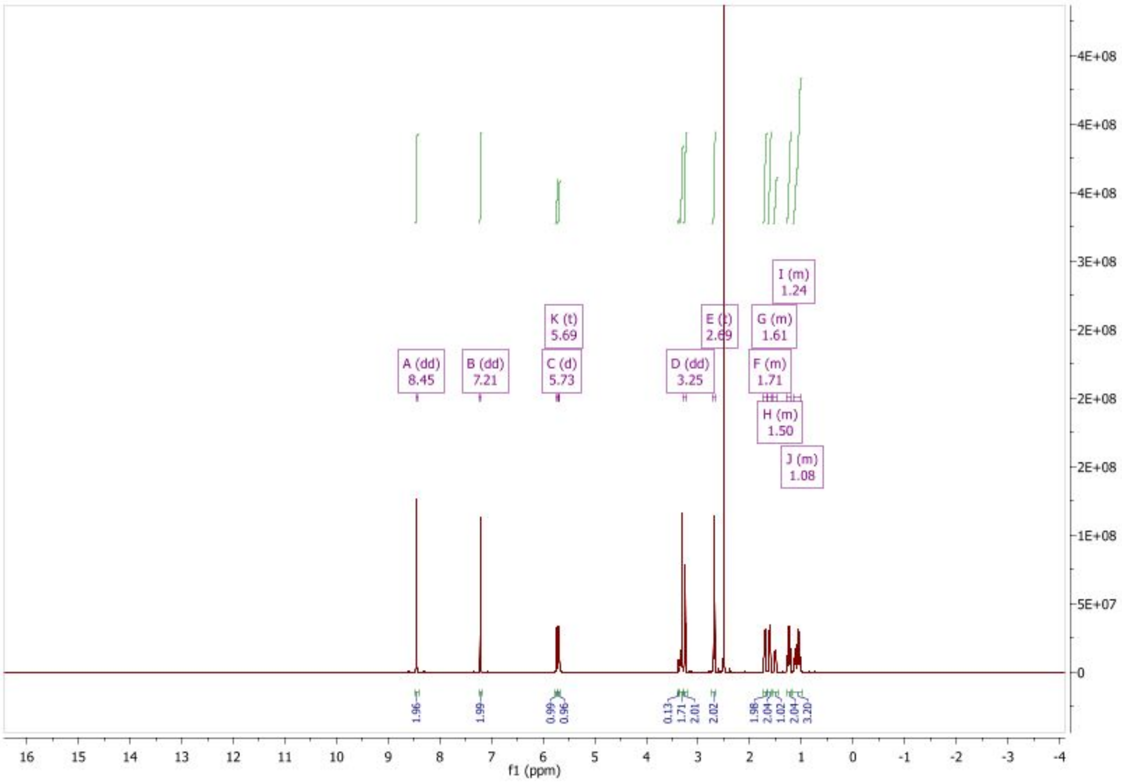

***N*-Isobutyl-2-methyl-3,8*a*-dihydroimidazo[1,2-*a*]pyridine-3-carboxamide (10)**

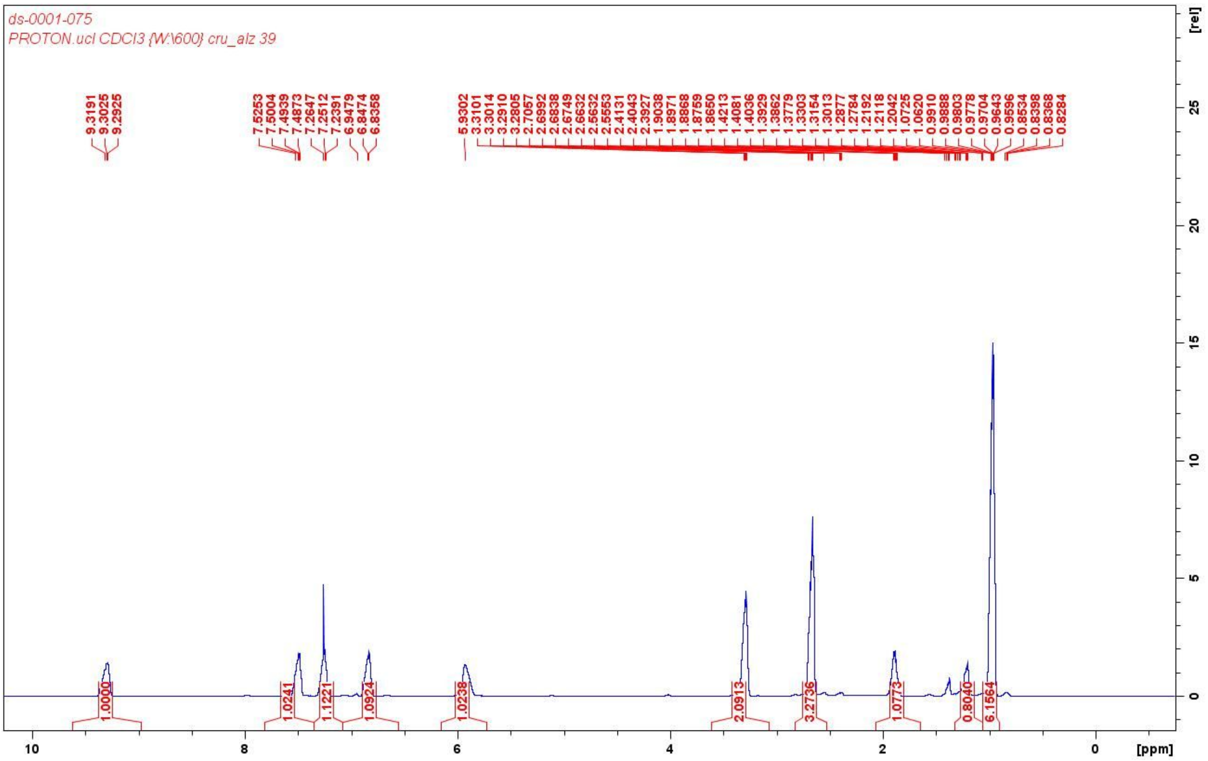

4-Amino-N-(2-pyridin-2-yl)benzenesulfonamide (11)

Compound **11** was purchased from Sigma Aldrich.

4-(Benzimidazol-1-ylmethyl)benzonitrile (12)

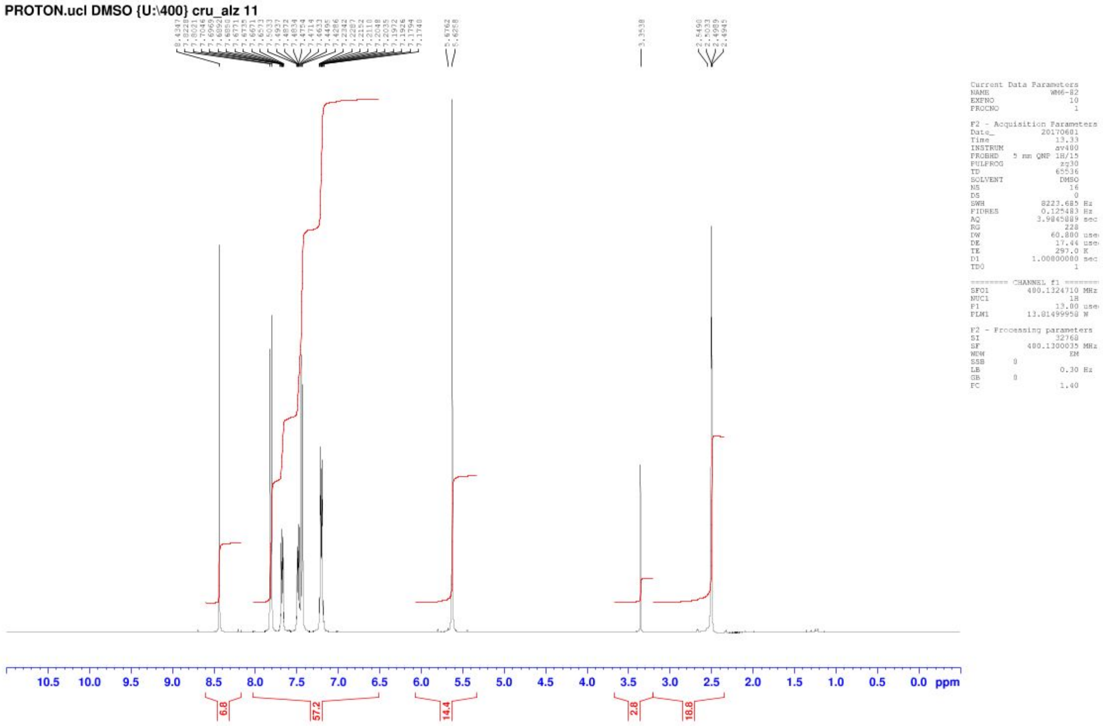

2-(Benzyloxy)aniline (13)

Compound **13** was purchased from Sigma Aldrich (189049).

2-[(1,1’-Biphenyl)-4-yl]acetic acid (14)

Compound **14** was purchased from Santa Cruz Biotechnology (sc-256684).

1*H*-Benzimidazol-2-ylcyanamide (15)

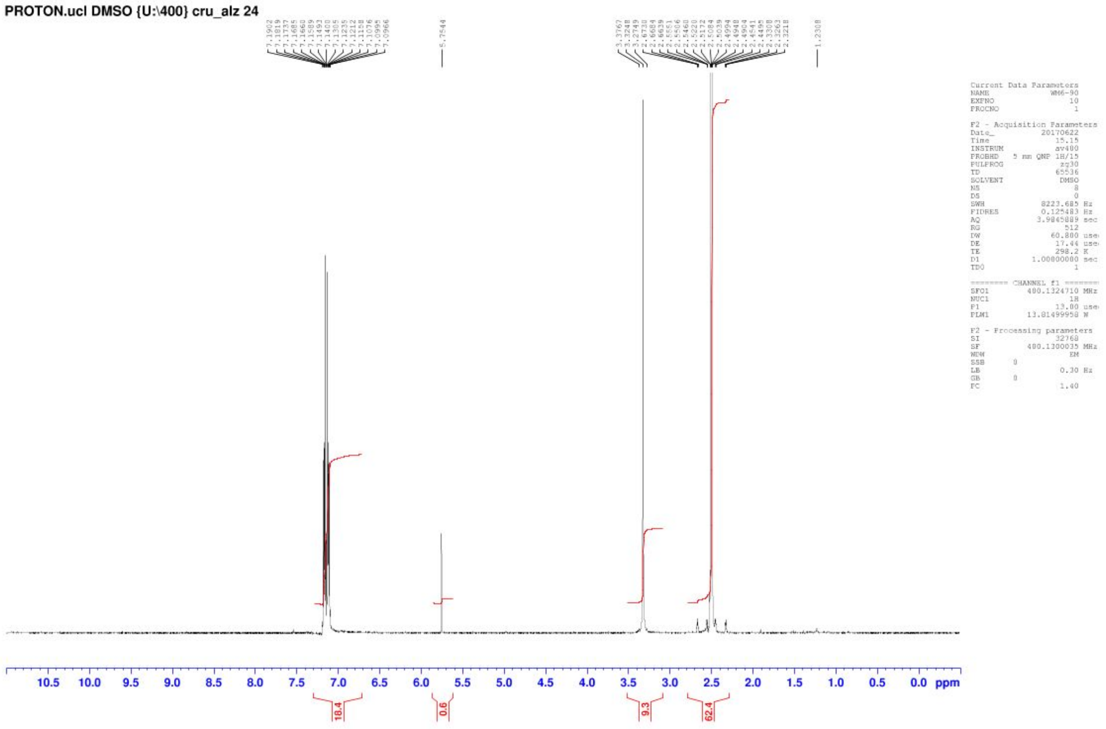

2,4-Difluoro-6-(1*H*-pyrazol-3-yl)phenol (16)

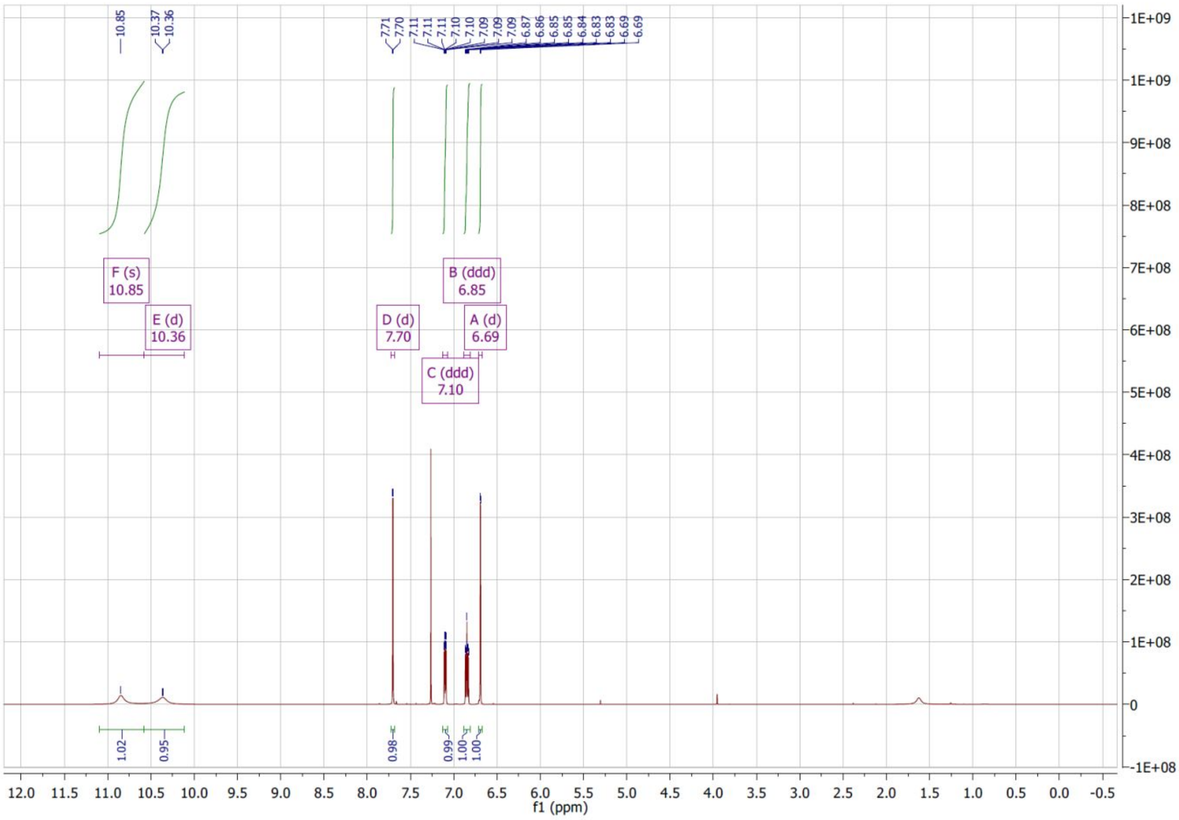

5-Methoxy-2-(1*H*-pyrazol-3-yl)phenol (17)

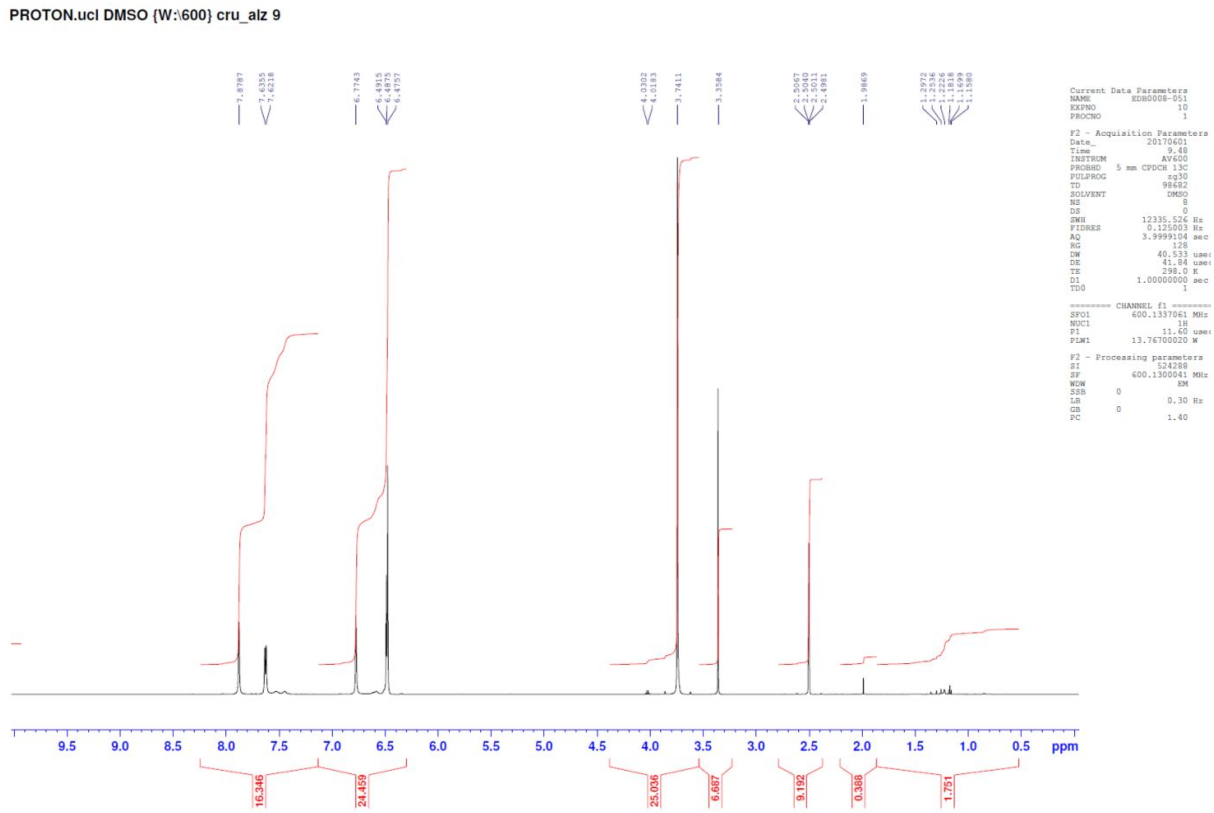

5-Amino-2-((pyridine-3-ylmethyl)amino)benzoic acid (18)

Compound **18** was purchased from Santa Cruz Biotech (sc-318110).

[(2*S*)-2-Methyl-1-piperidyl]-morpholino-methanone (19)

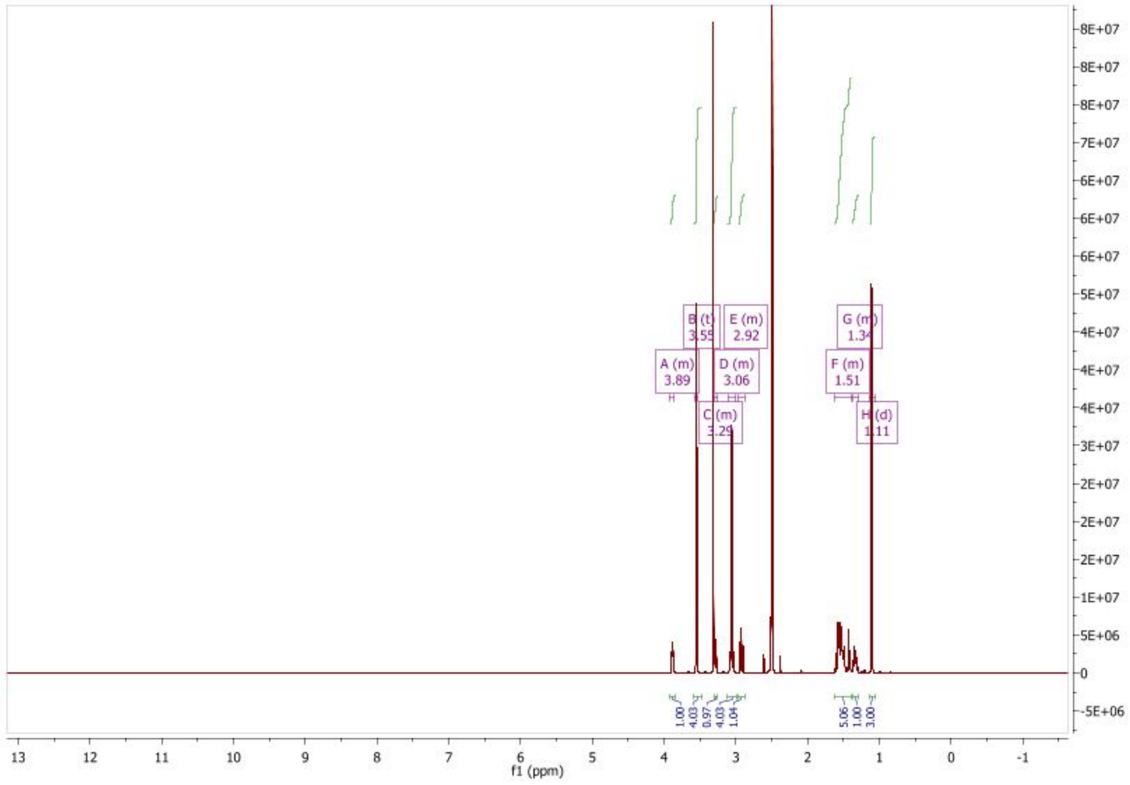

4-(1-Piperidyl)-1,2,5-oxadiazol-3-amine (20)

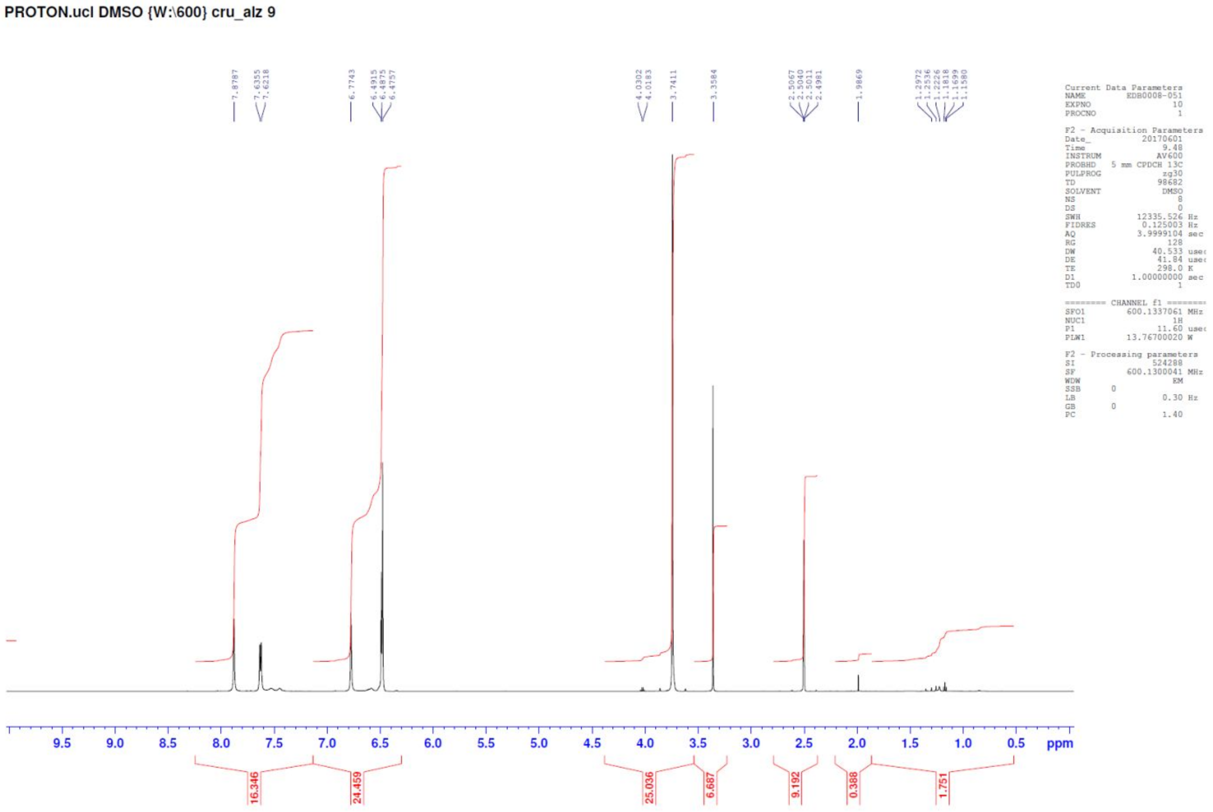

N-Methyl-N-[(5-methyl-2-furyl)methyl]-1-phenyl-methanamine (21)

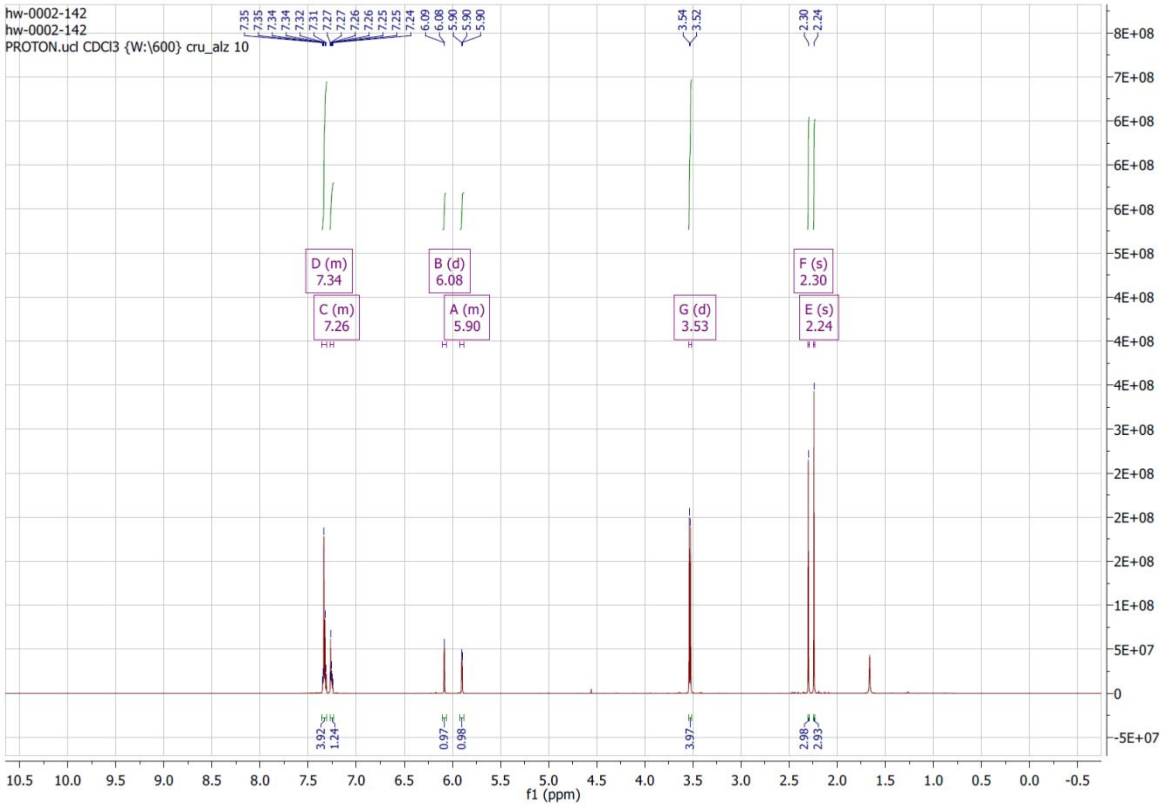

5-(3-Ethoxyphenyl)-1,3-4-thiadiazol-2-amine (22)

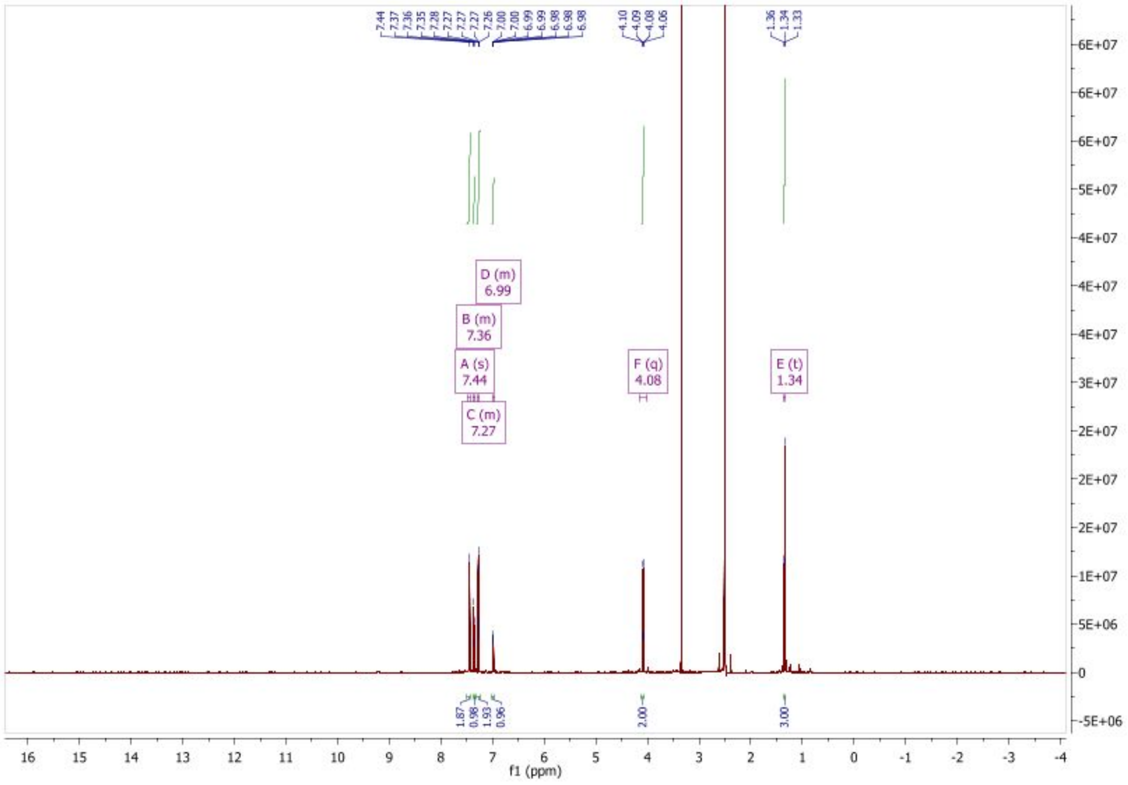

2-(4-(3-Chlorophenyl)piperazin-1-yl)acetonitrile (23)

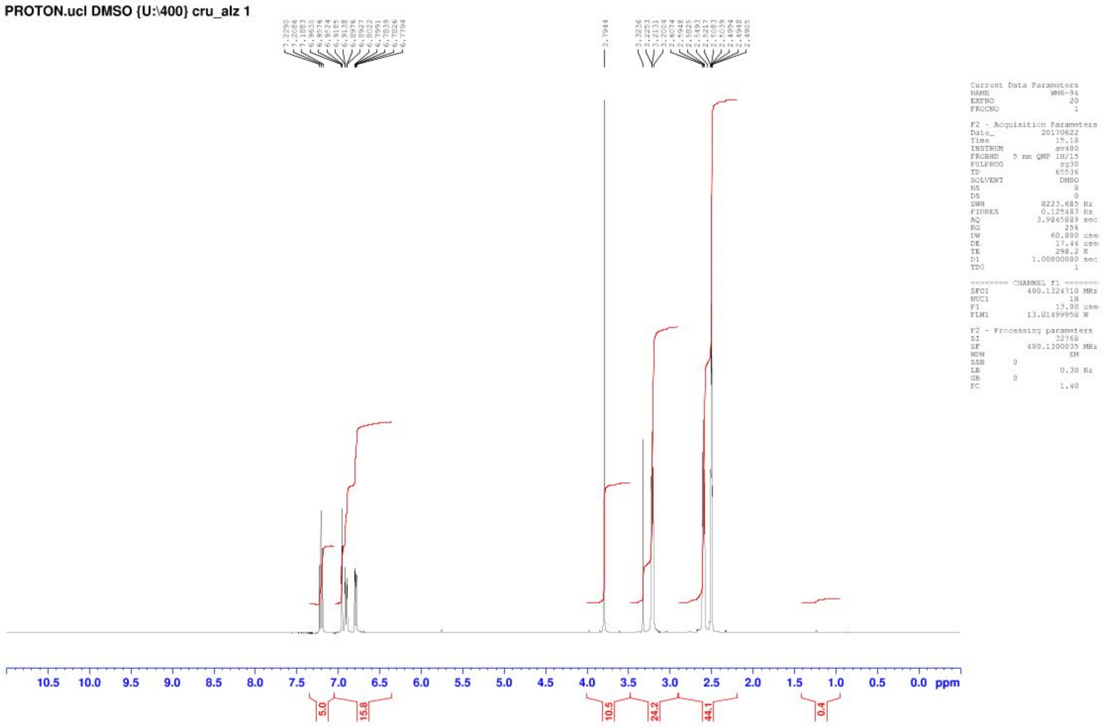

3-(Phenylisoxazol-5-yl)methanamine (24)

Compound **24** was purchased from Maybridge.

N-(4-Anilinophenyl)acetamide (25)

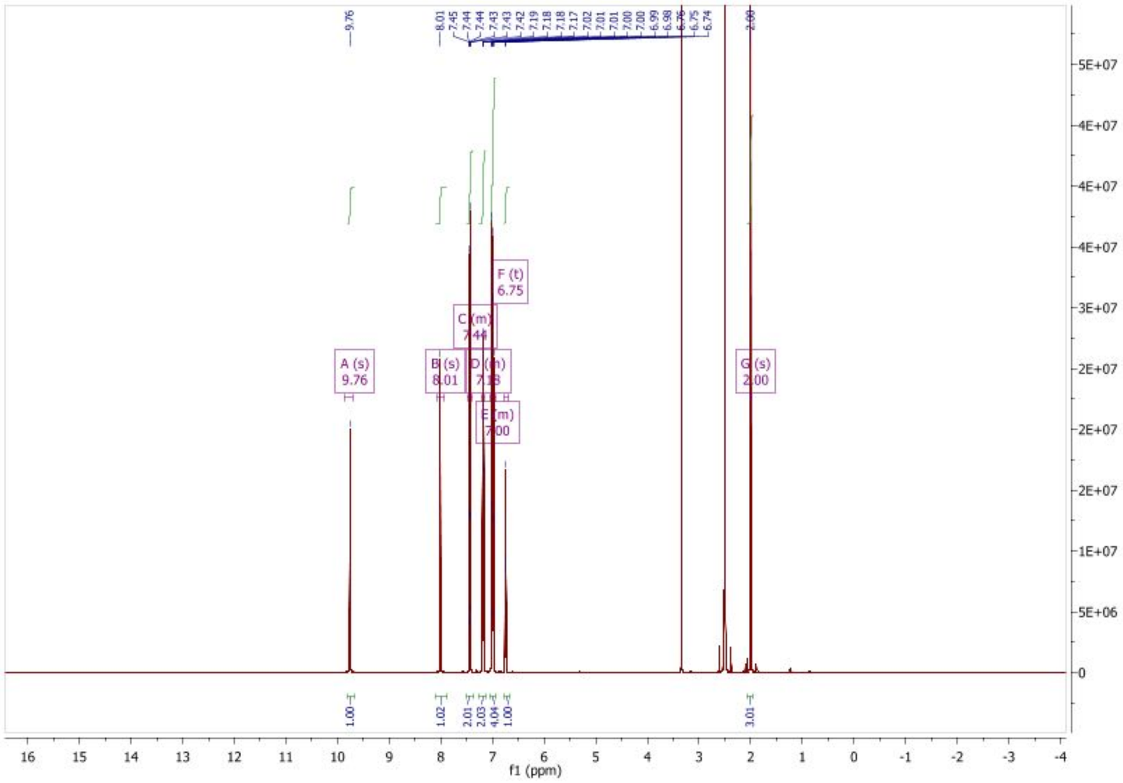

[1-(4-Chlorophenyl)-1H-1,2,3-triazol-4-yl]methanol (26)

Compound **26** was purchased from Key Organics (4F-329S).

(4-Acetamidophenoxy)acetic acid (27)

Compound **27** was purchased from Key Organics (SS-3896).

1-(Benzo[d][1,3]dioxol-5-yl)-N-(pyridine-2-ylmethyl)methanamine (28)

Compound **28** was purchased from Santa Cruz Biotech (sc-326192).

Benzyl N-hydroxycarbamate (29)

Compound **29** was purchased from Key Organics (MS-2251).

4-(2-Furylmethyl)-1,4-thiazinane 1,1-dioxide hydrochloride (30)

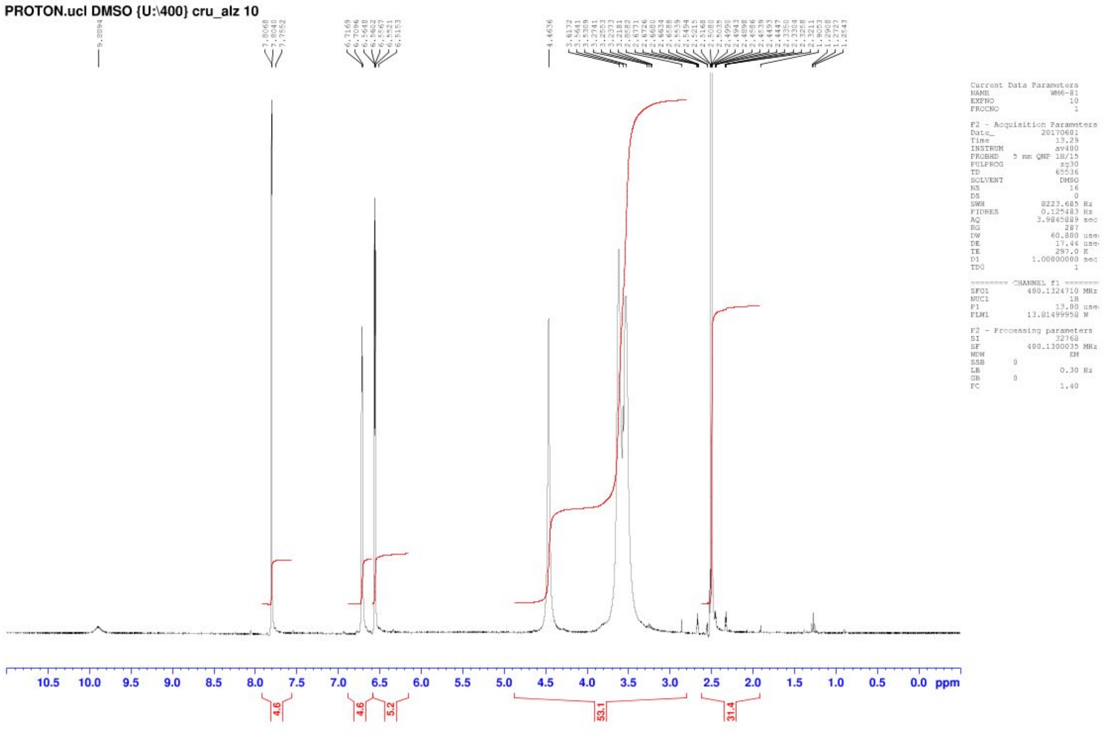

2-(4-Ethylphenoxy)-1-(1-piperidyl)ethenone (31)

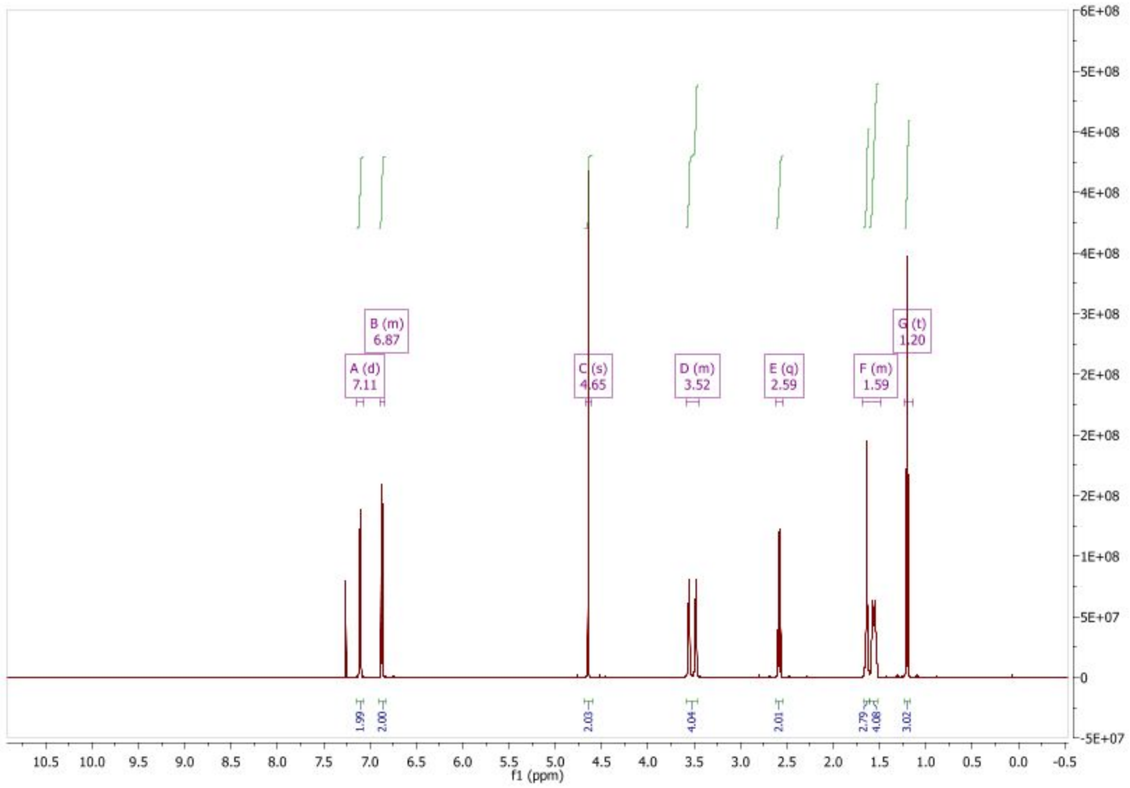

(*R*)-1-(Benzo[*d*][1,3]dioxol-5-yl)-*N*-((tetrahydrofuran-2-yl)methyl)methanamine (32)

Compound **32** was purchased from Santa Cruz Biotech (sc-326178).

***N*[1]-(Pyridin-2-yl)benzene-1,2-diamine (33)**

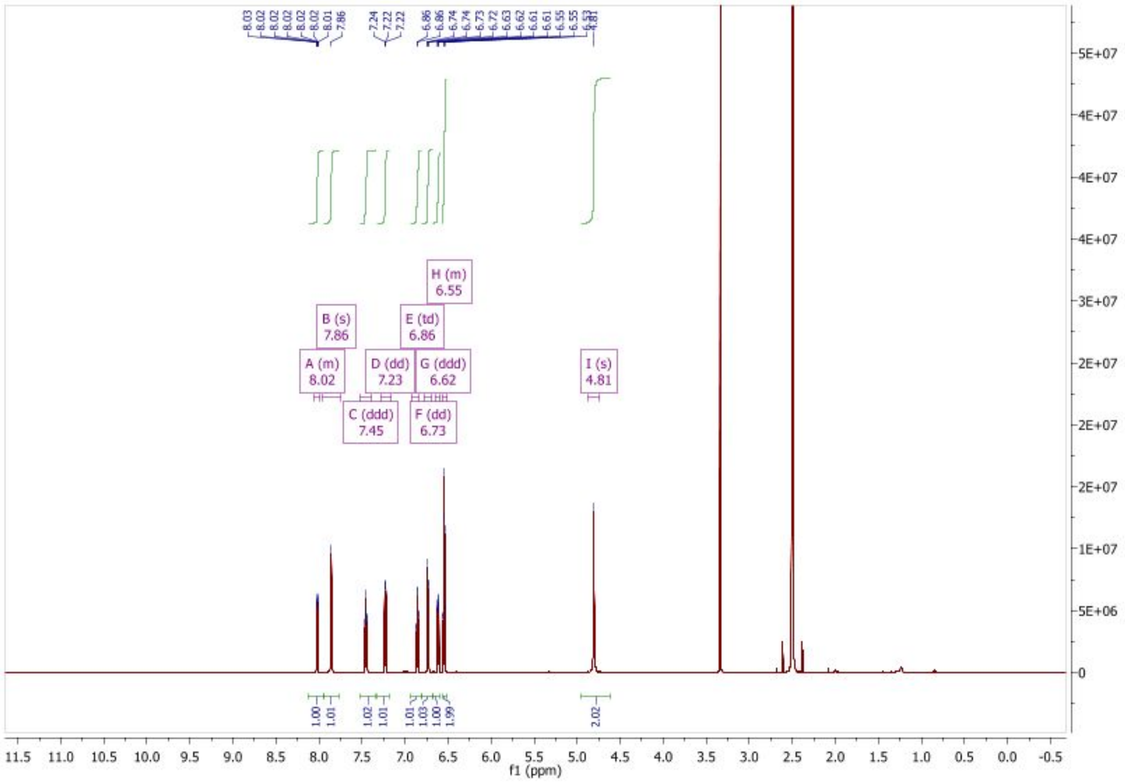

2-(Benzyloxy)benzohydrazide (34)

Compound **34** was purchased from Santa Cruz Biotech (sc-305895).

2-Methoxy-*N*-(4-phenylthiazol-2-yl)acetamide (35)

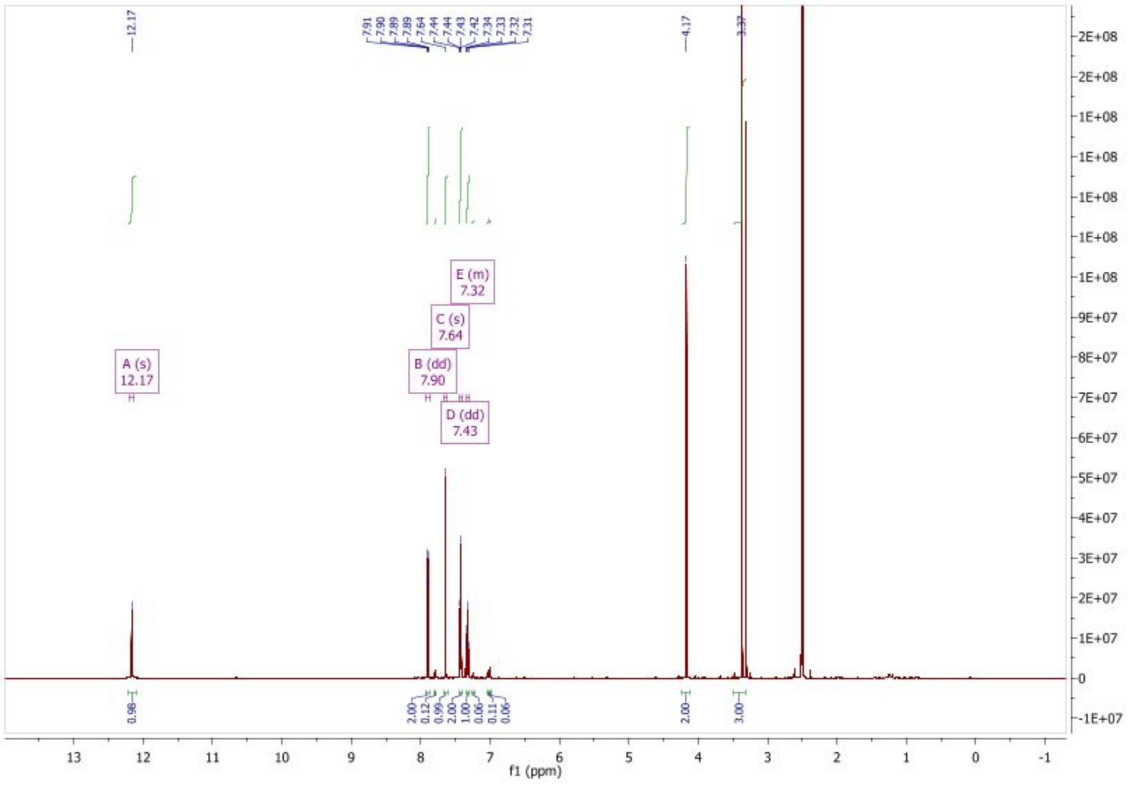

4-(4-Pyrrol-1-ylphenyl)morpholine (36)

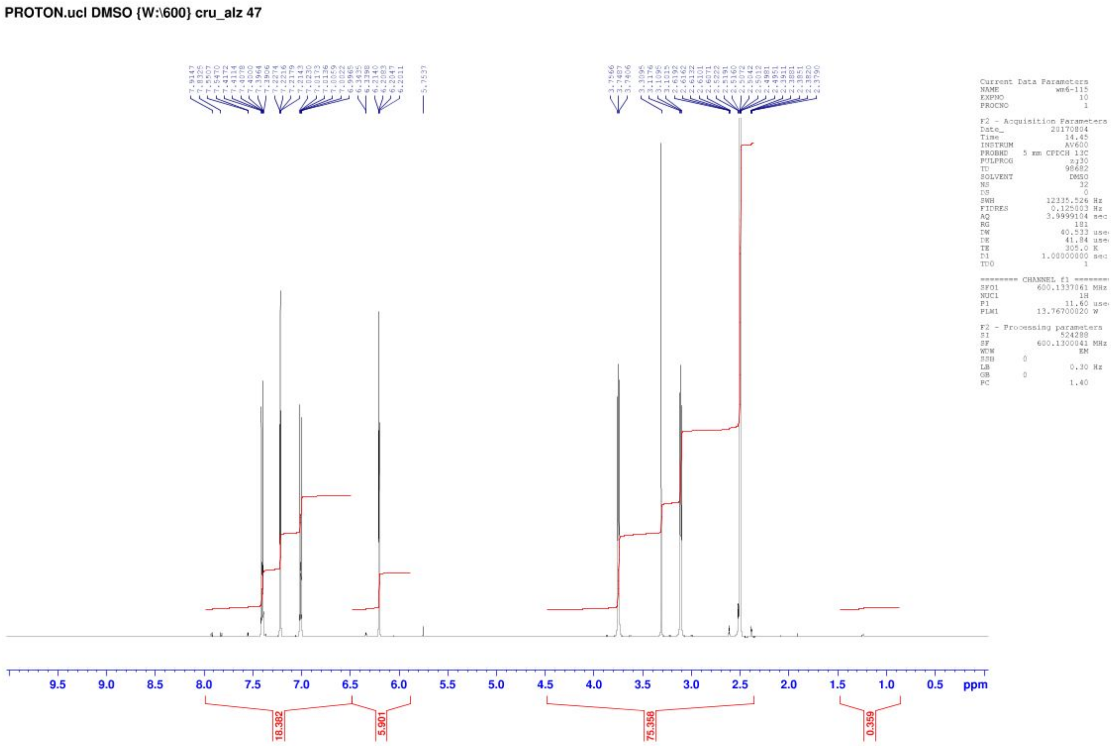

2-(Morpholinomethyl)naphthalen-1-ol hydrochloride (37)

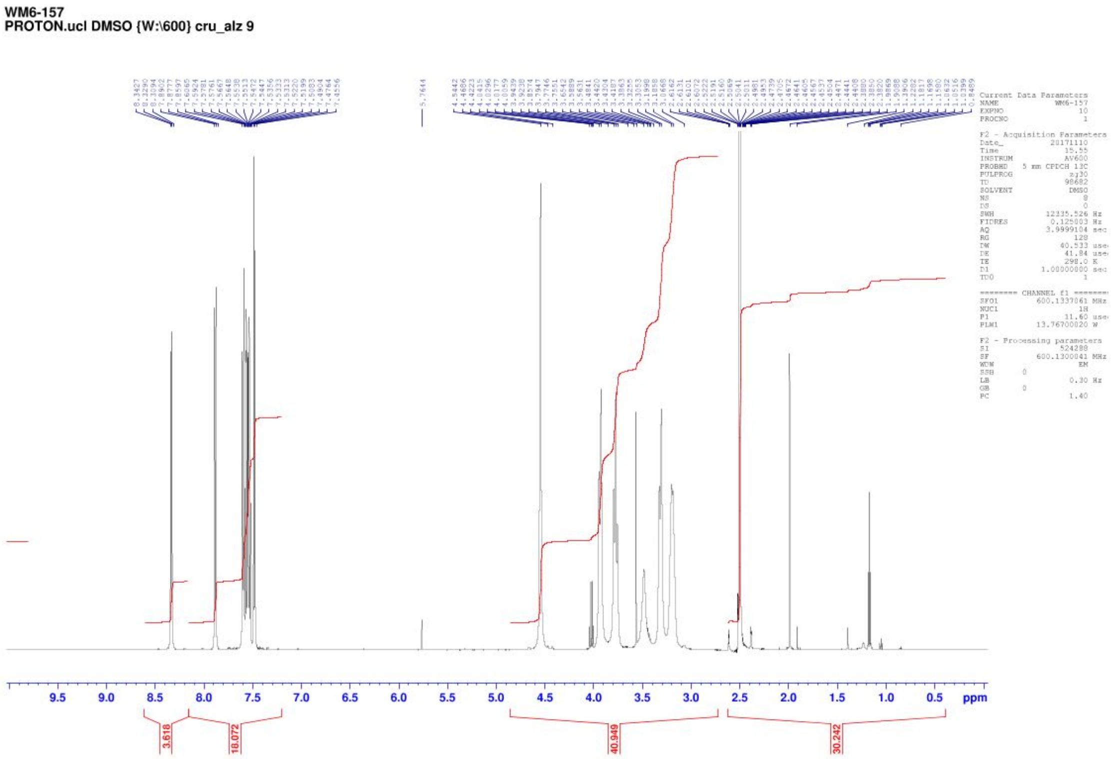

3-((4-Methylpiperidin-1-yl)methyl)-1*H*-indole (38)

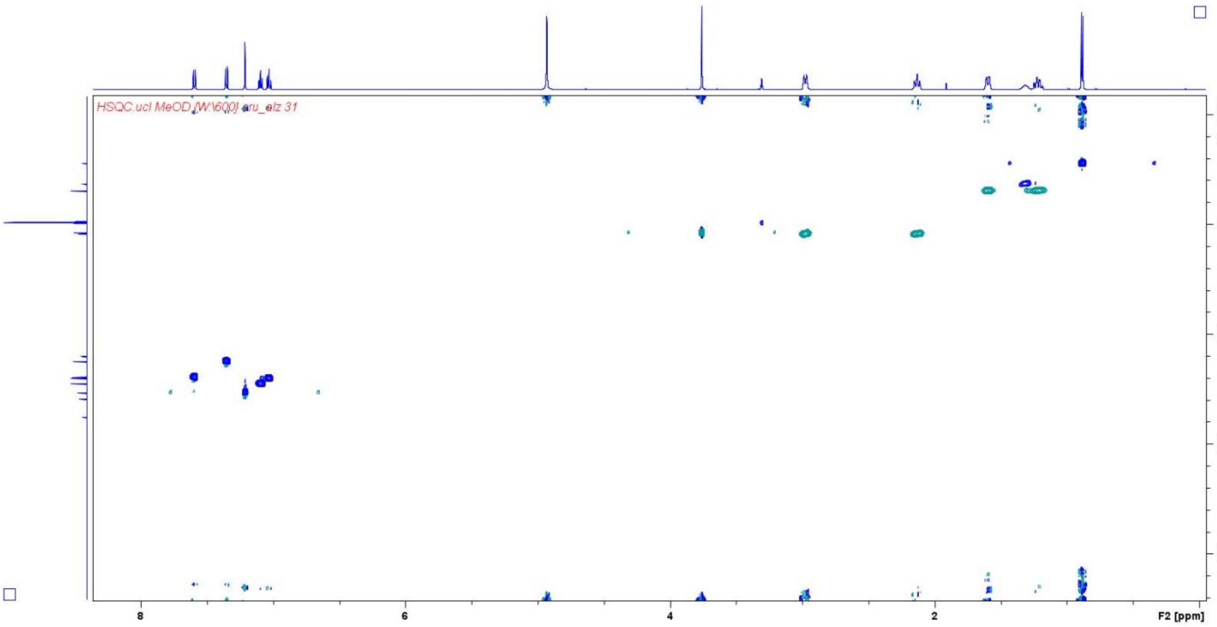

2-(4-(2,5-Dioxopyrrolidin-1-yl)phenoxy)acetic acid (39)

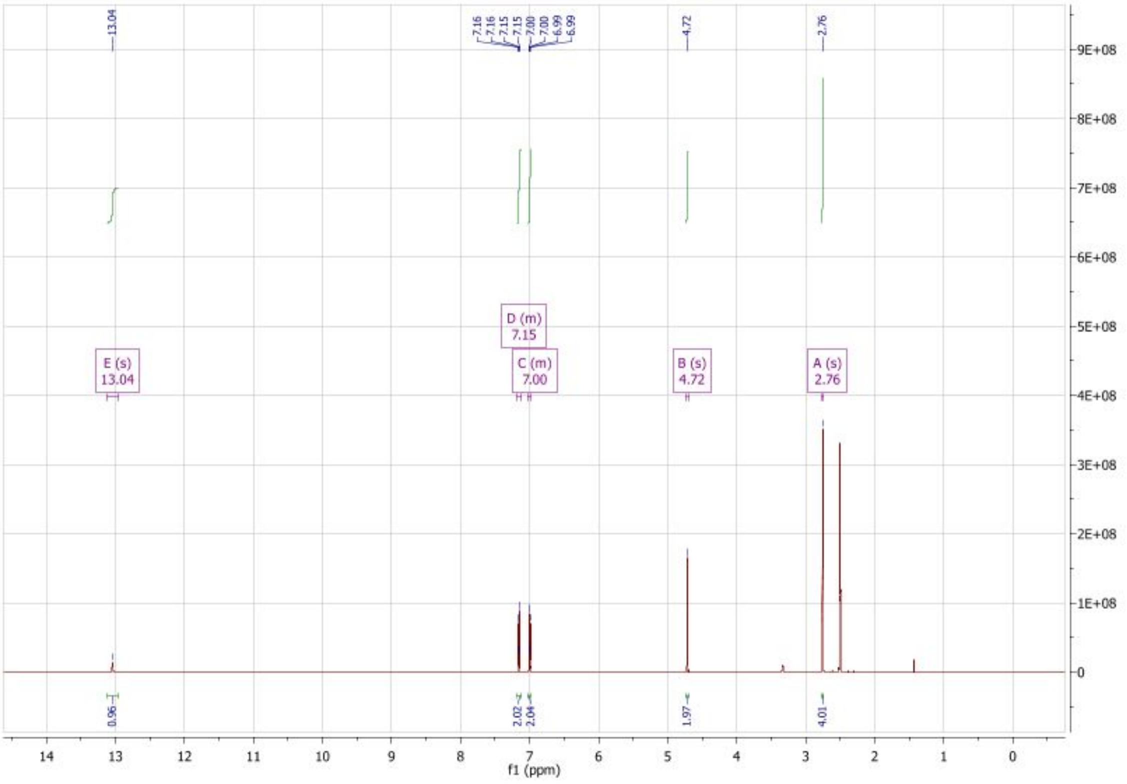

2-(2-Methylphenoxy)-N-(3-pyridyl)acetamide (40)

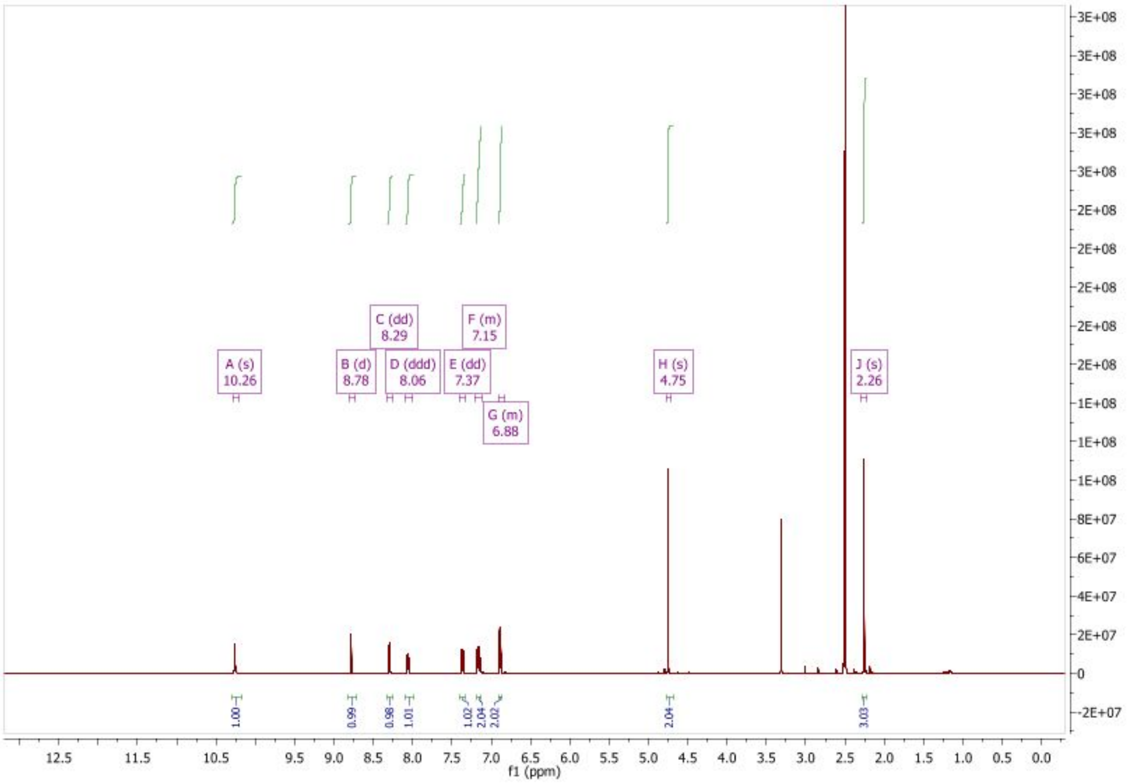

***N*-Benzyl-2-methoxy-acetamide (41)**

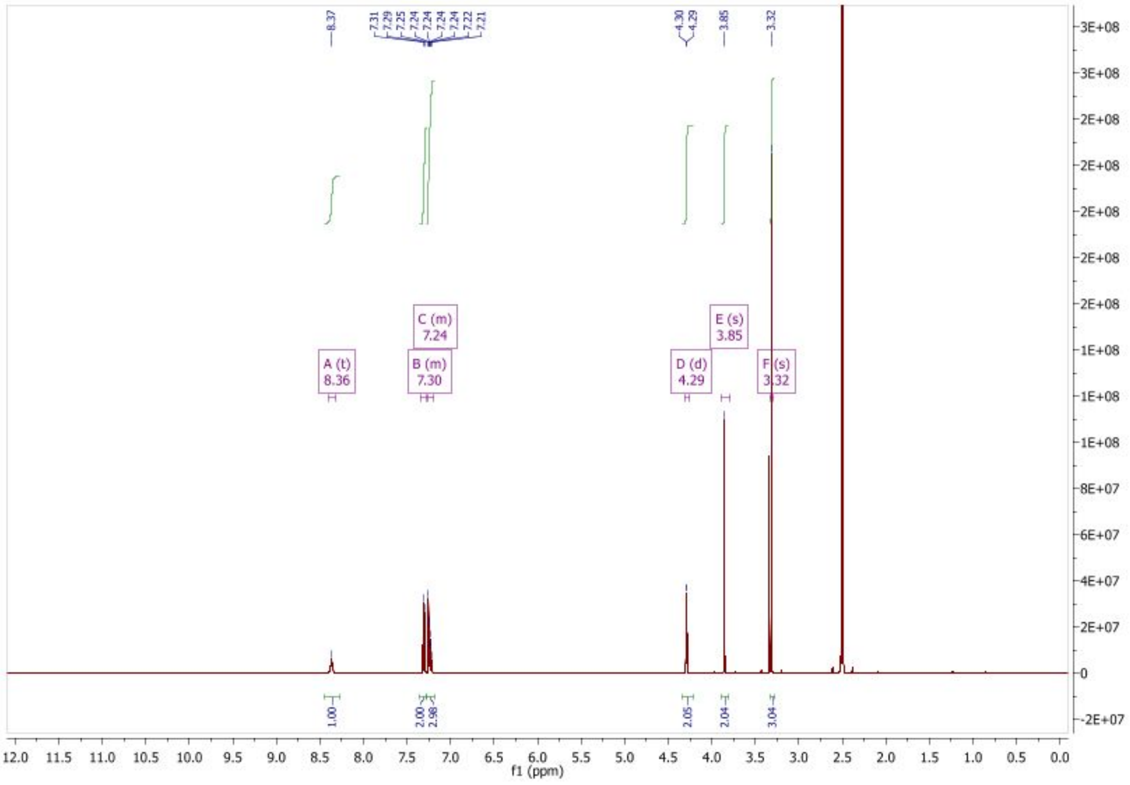

***N*-(2-Hydroxy-4-methylphenyl)propanamide (42)**

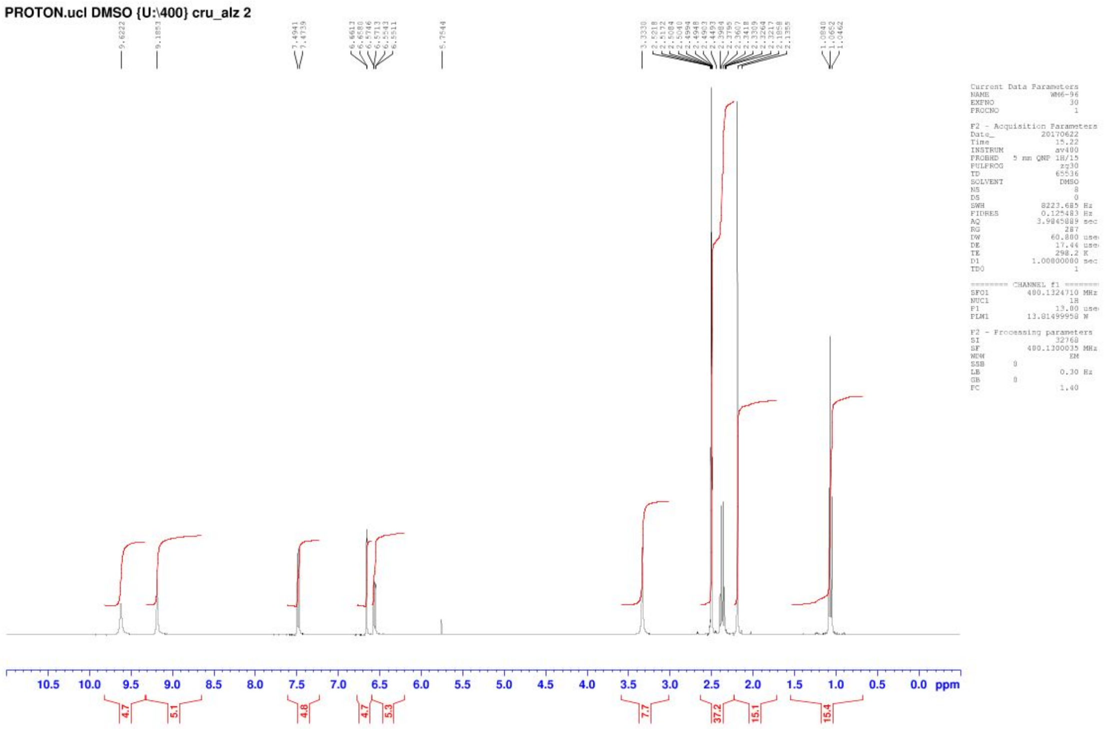

(2*S*)-*N*-[(4-Methoxyphenyl)methyl]tetrahydrofuran-2-carboxamide (43)

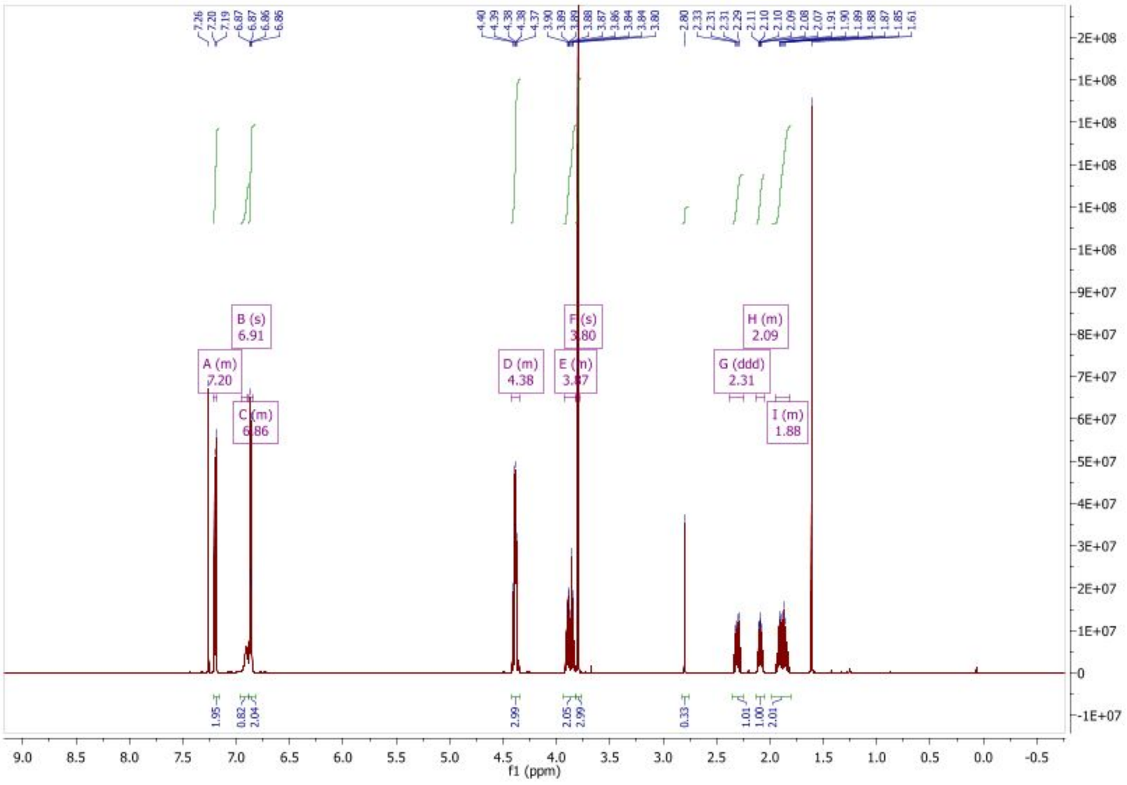

Methyl 2-(4-cyanophenoxy)acetate (44)

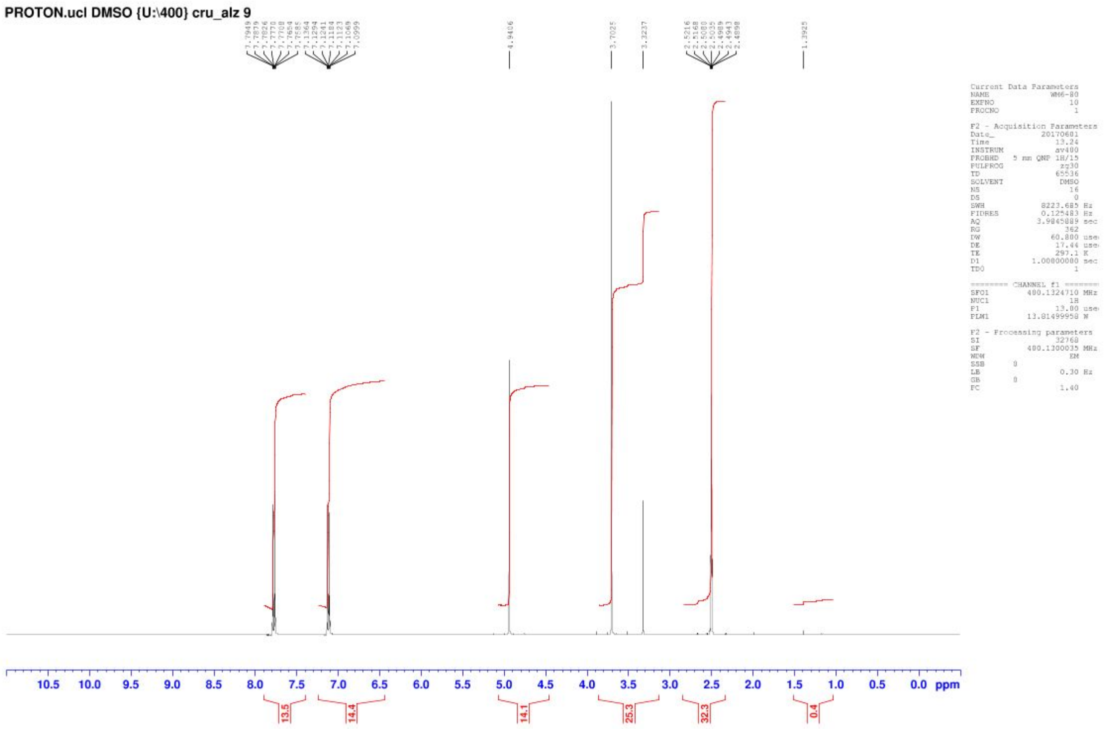

_N_-[2-(4-Hydroxyphenyl)ethyl]pyridine-2-carboxamide (45)

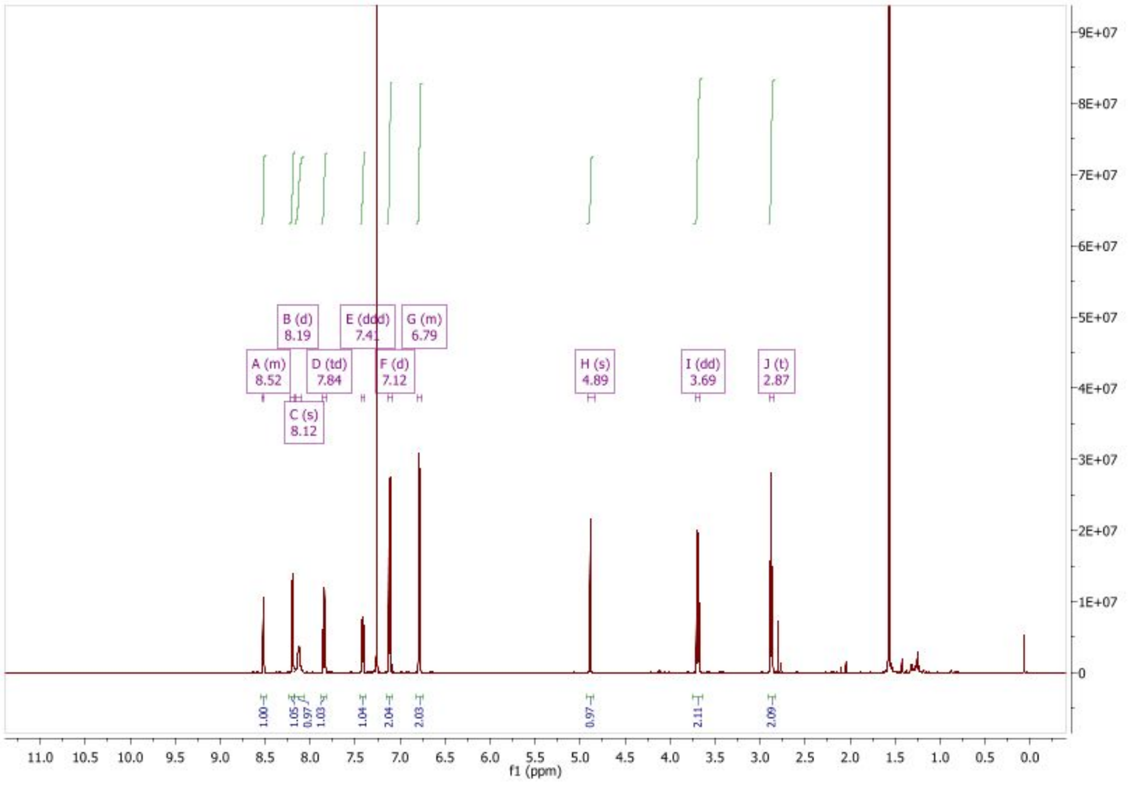

4-Methoxy-6-phenyl-pyrimidin-2-amine (46)

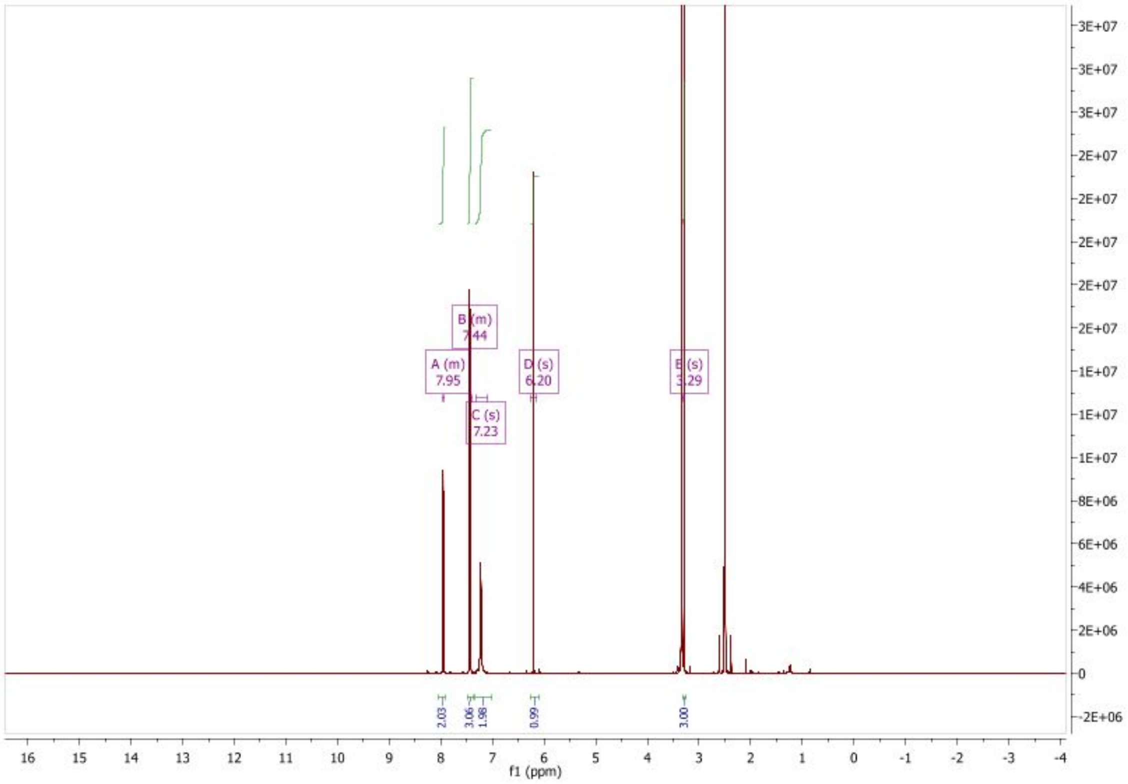

1-(4-Methoxyphenyl)-3-(pyridin-4-yl)urea (47)

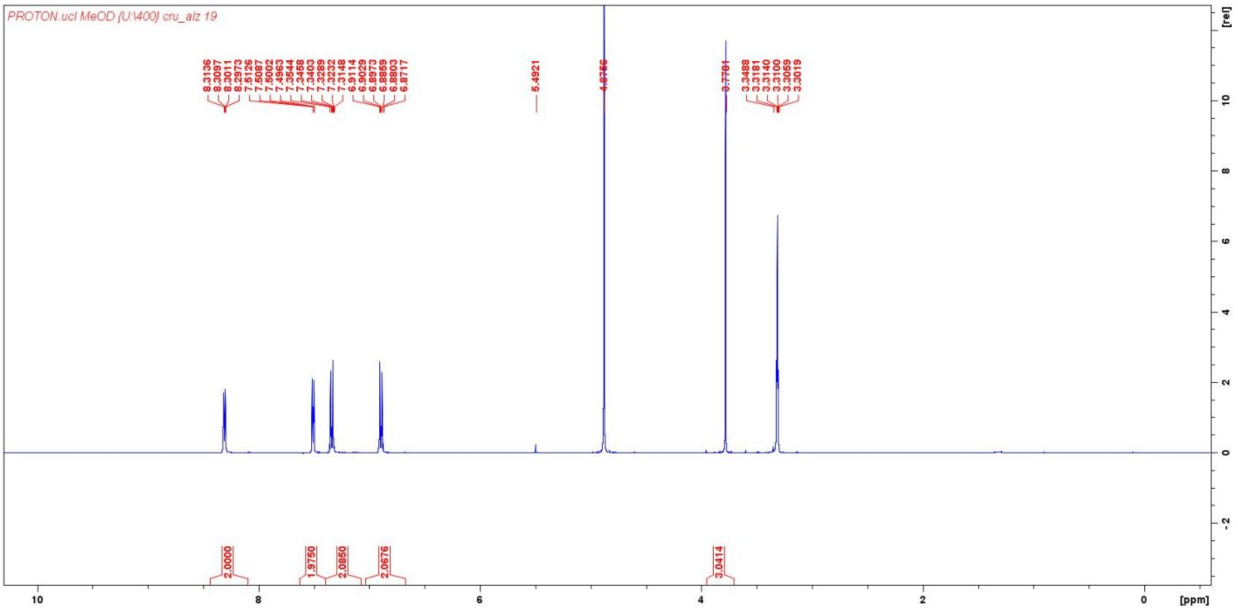

2-(4-Ethoxyphenyl)acetic acid (48)

Compound **48** was purchased from Alfa Aesar.

1-Methyl-5-(phenylamino)-1*H*-pyrazol-3-ol (49)

Compound **49** was purchased from Fluorochem (478474).

2,5-Dimethylphenyl isonicotinate (50)

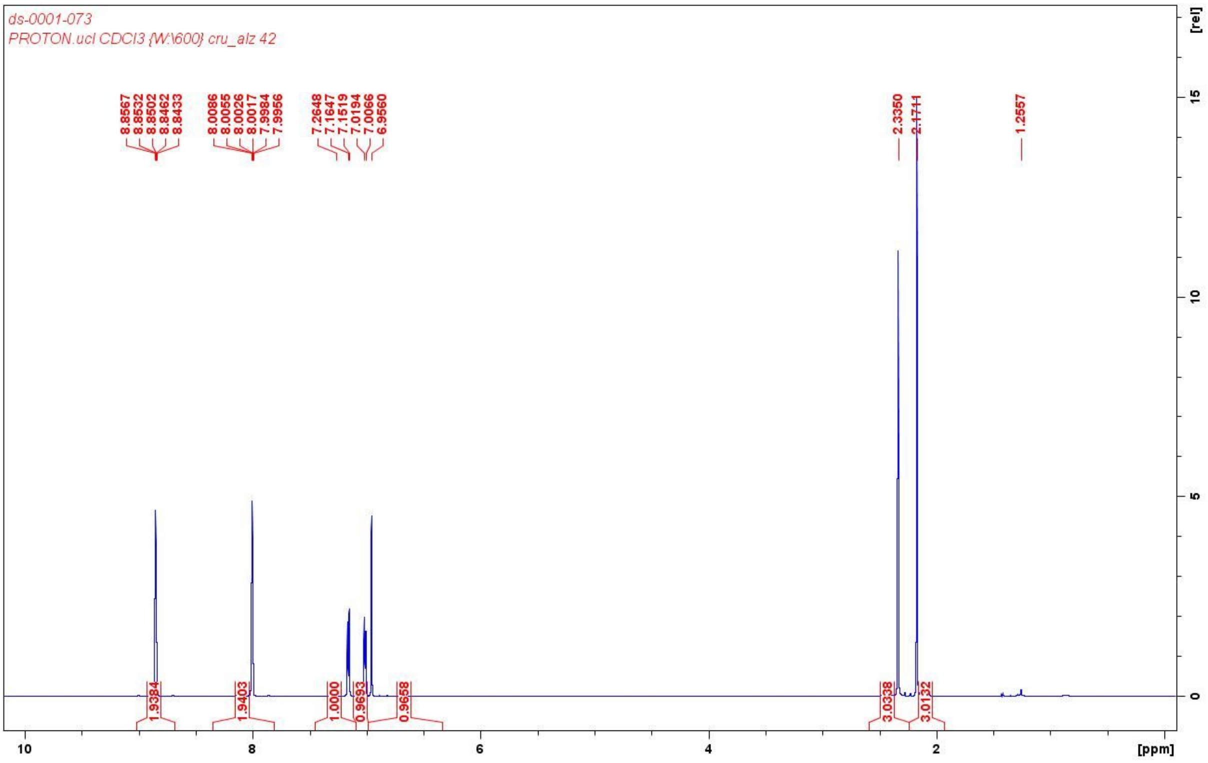

(4-Chloro-2-methyl-pyrazol-3-yl)-(1-piperidyl)methanone (51)

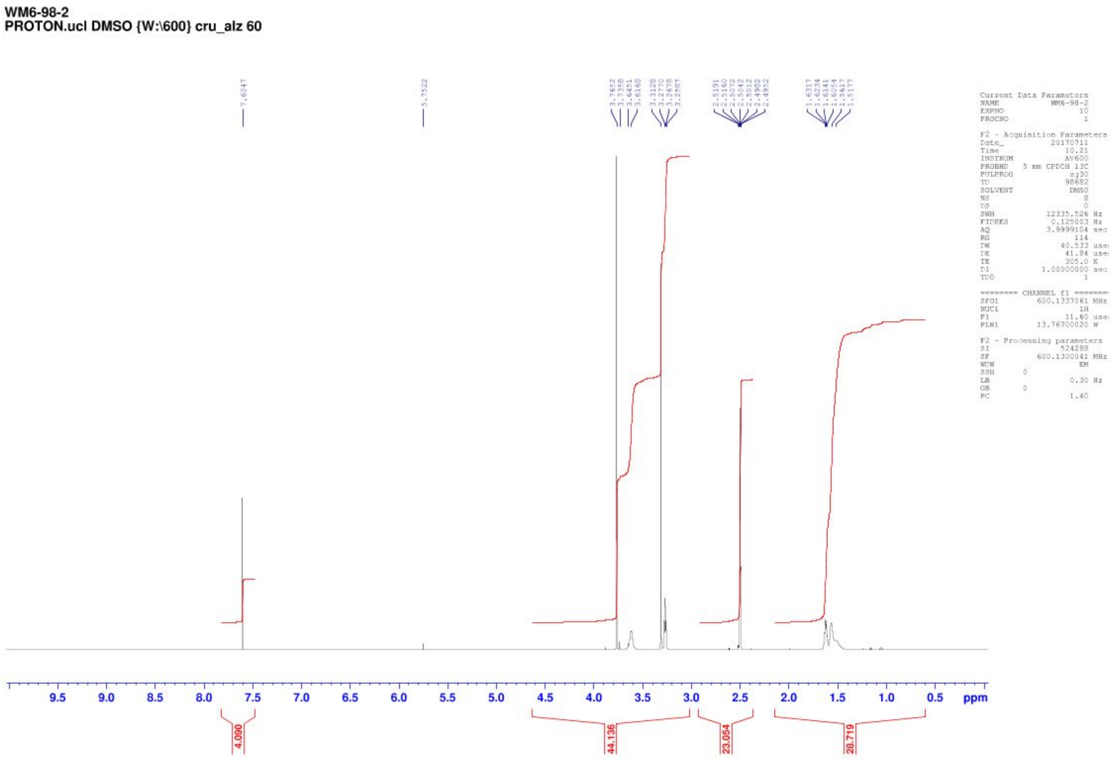

2-(Benzyloxy)acetic acid (52)

Compound **52** was purchased from Acros.

5-Ethyl-3-methyl-*N*-(5-methylisoxazol-3-yl)isoxazole-4-carboxamide (53)

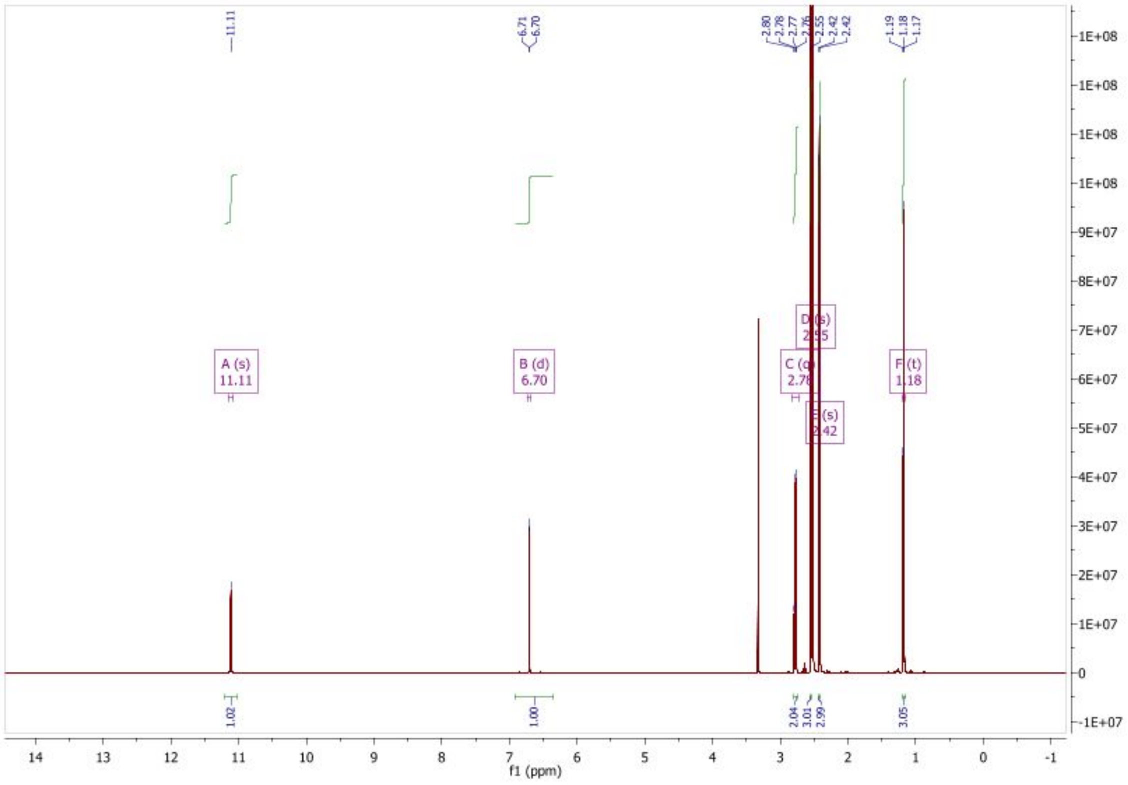

1-Methyl-*N*-(*o*-tolylmethyl)tetrazol-5-amine (54)

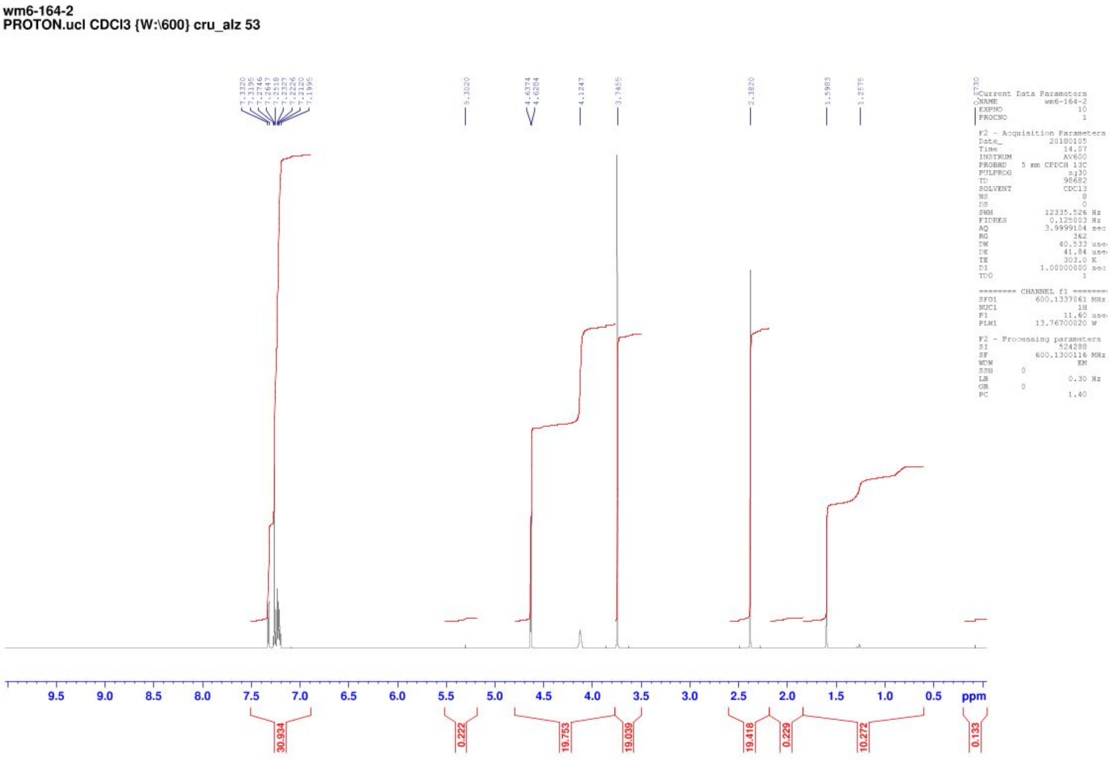

(*R*)-2-([1,1'-Biphenyl]-4-yloxy)propanoic acid (55)

Compound **55** was purchased from Santa Cruz Biotech (sc-334586).

4-(2-Phenoxyacetyl)piperazin-2-one (56)

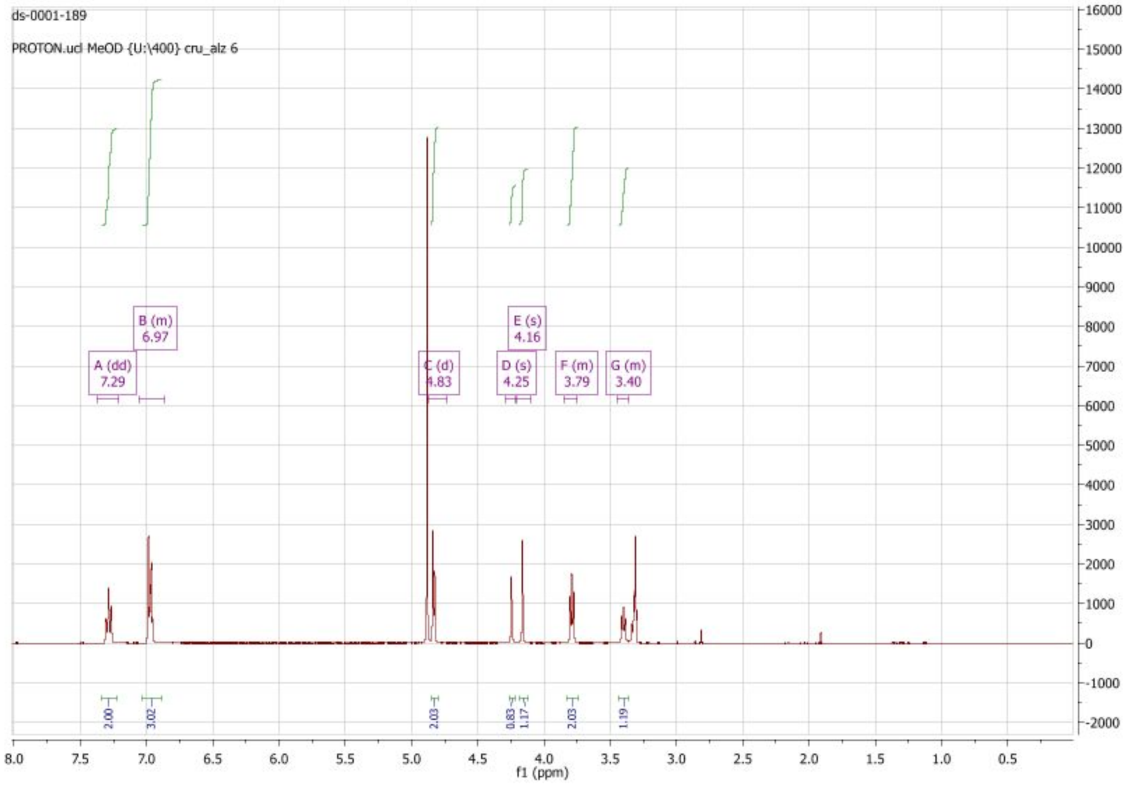

N-(1-Ethylbenzimidazol-2-yl)acetamide (57)

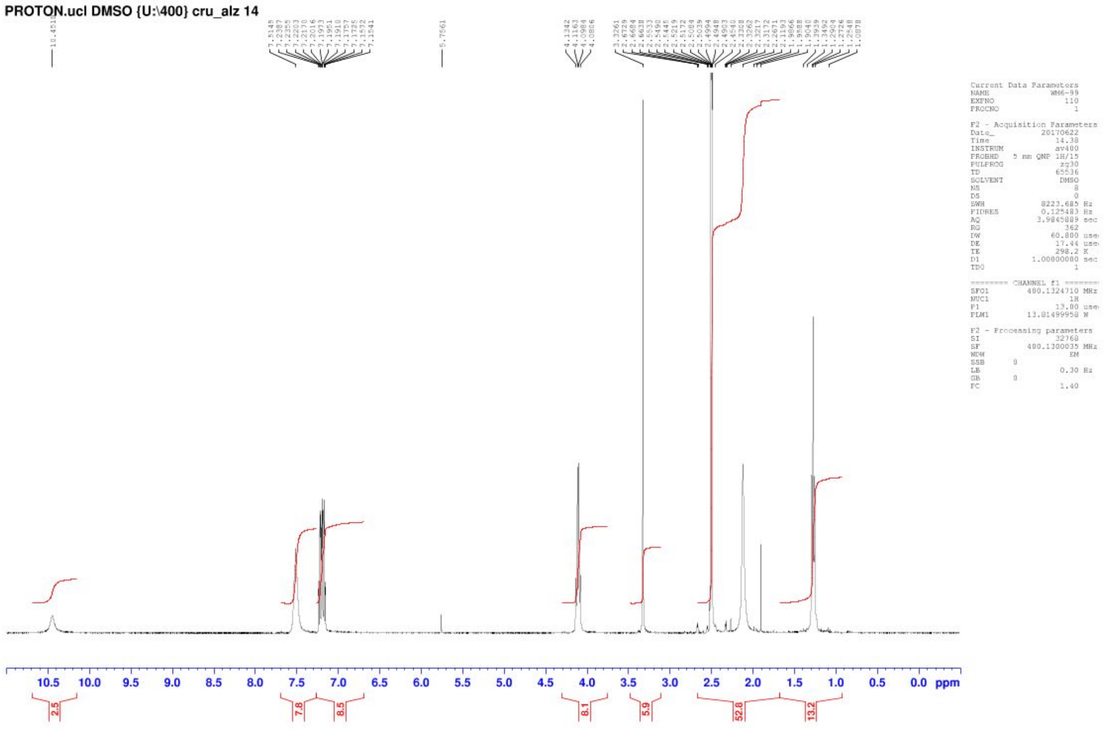

N-(2,1,3-benzothiadiazol-5-yl)acetamide (58)

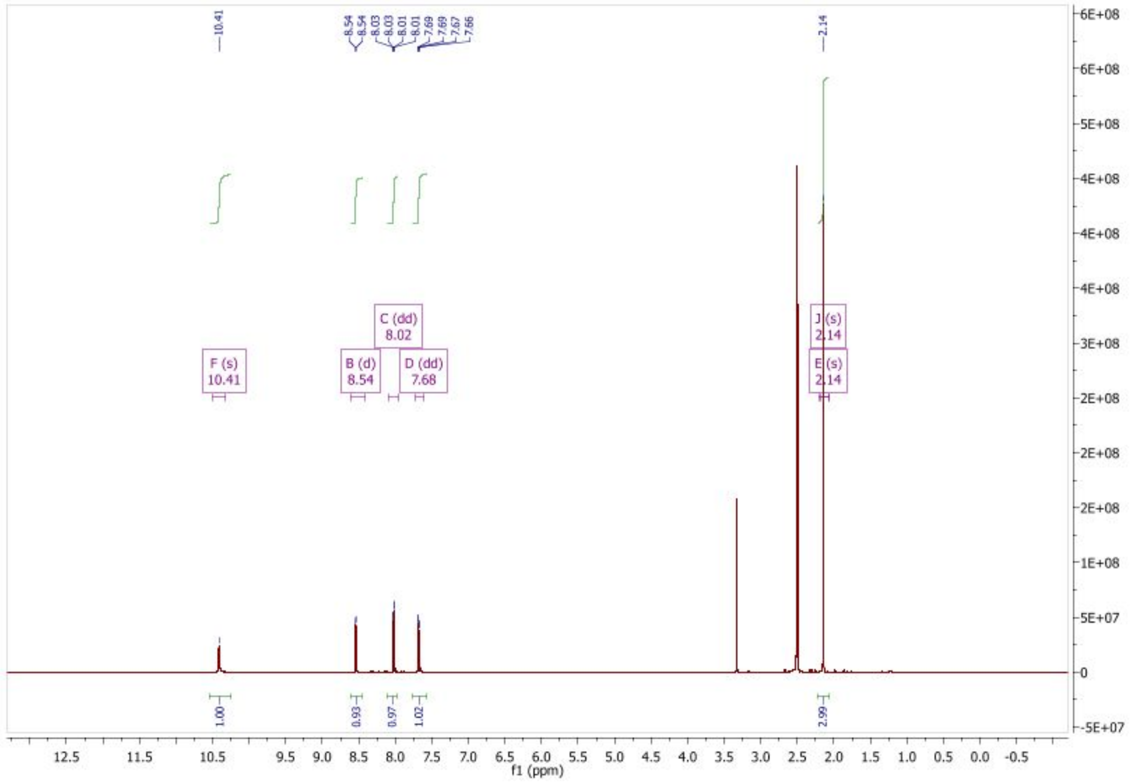

Figure S3. Spectroscopic and Analytical data for Fragment Development Compounds 7b, 15a, 16a and 28a.

A: ^{1}H NMR for 7b.

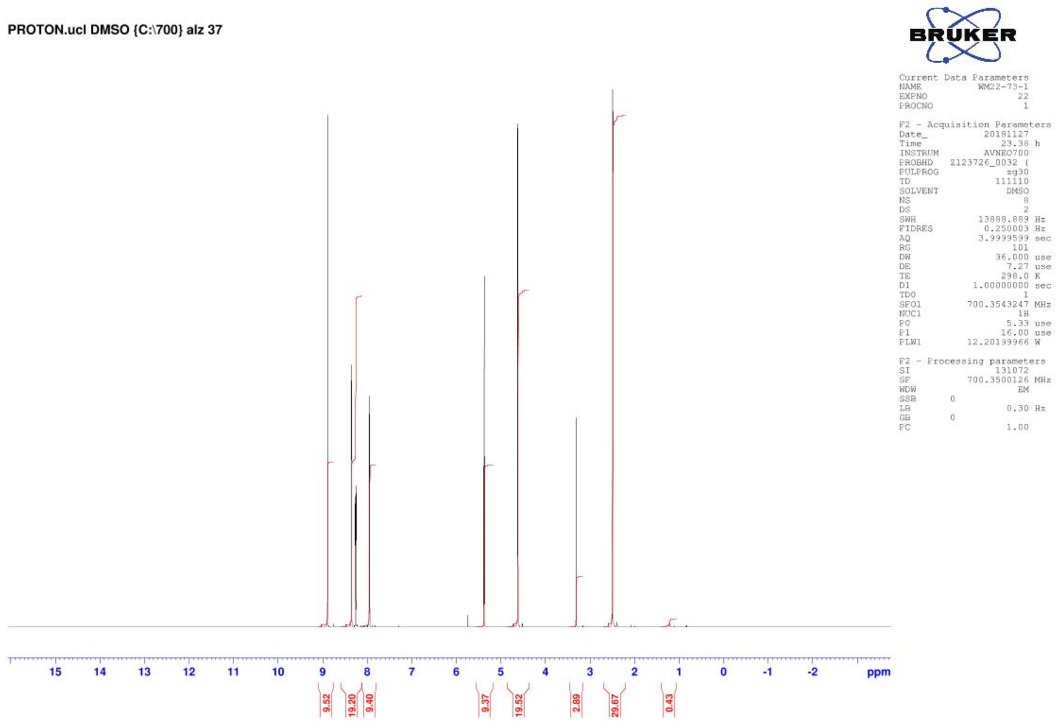

B: ^{13}C NMR for 7b.

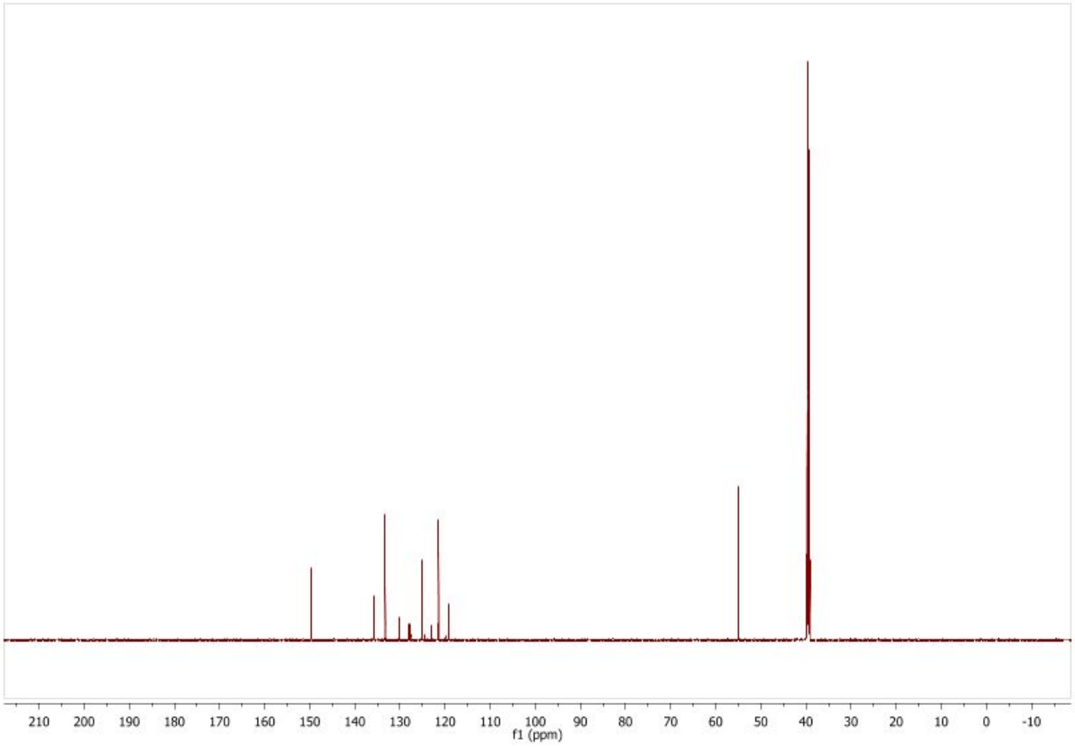

C: LCMS for 7b.

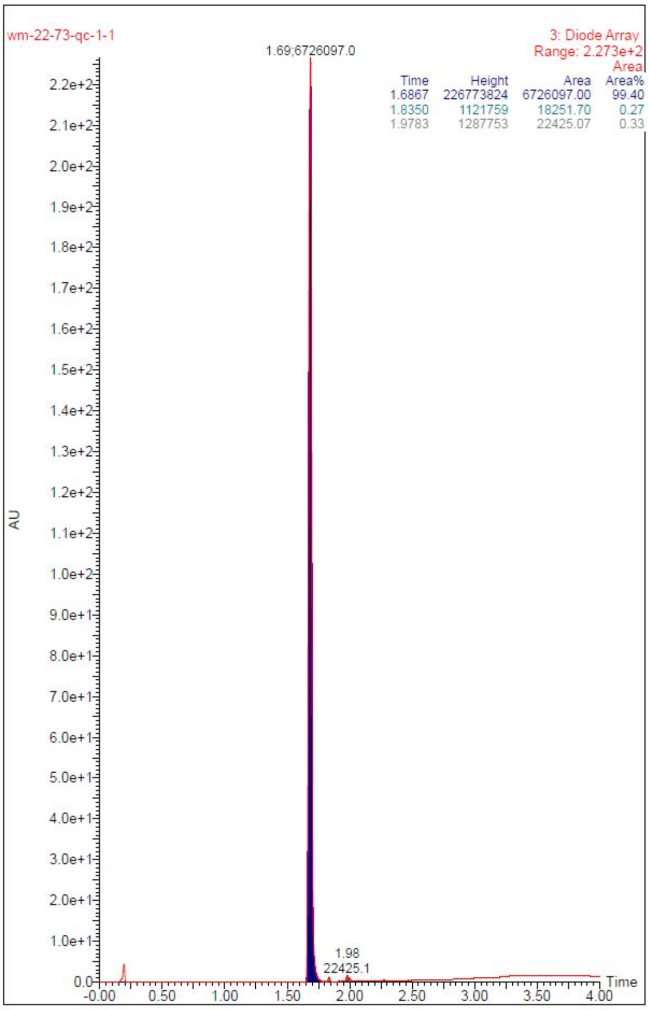

Peak ID	Compound	Time	Mass Found
4		1.55	Not Found

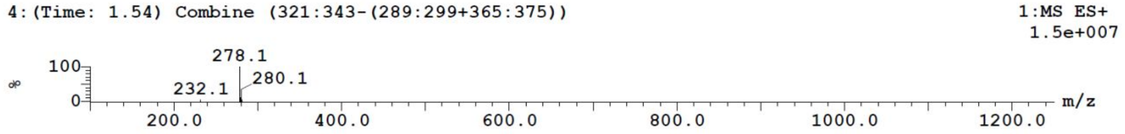

D: ^{1}H NMR for 15a.

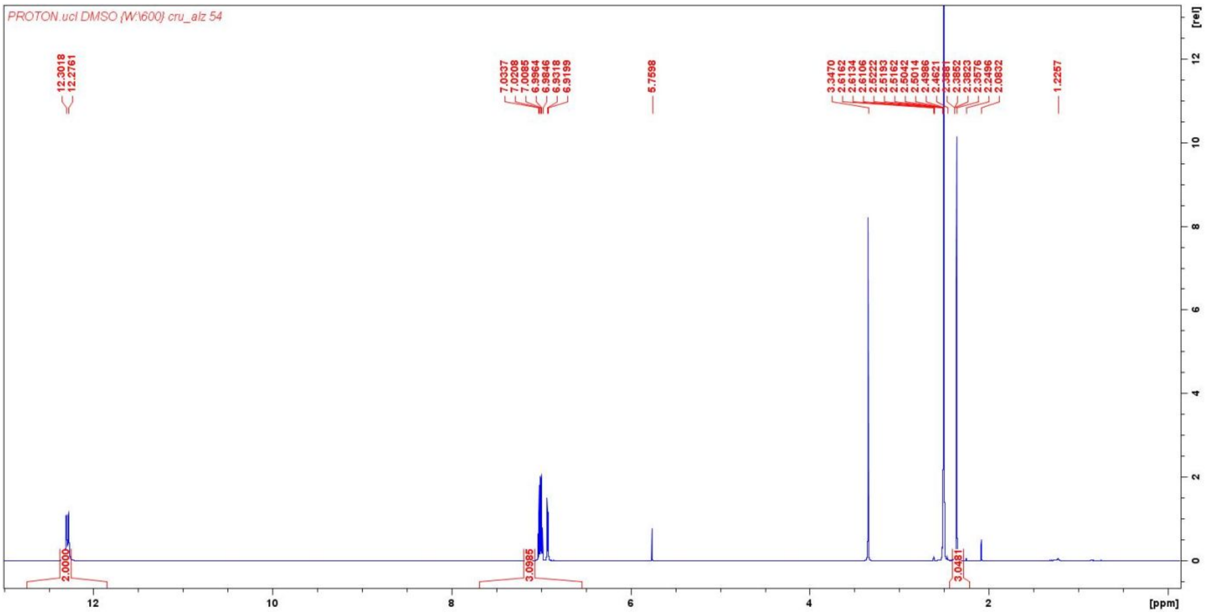

E: ^{13}C NMR for 15a.

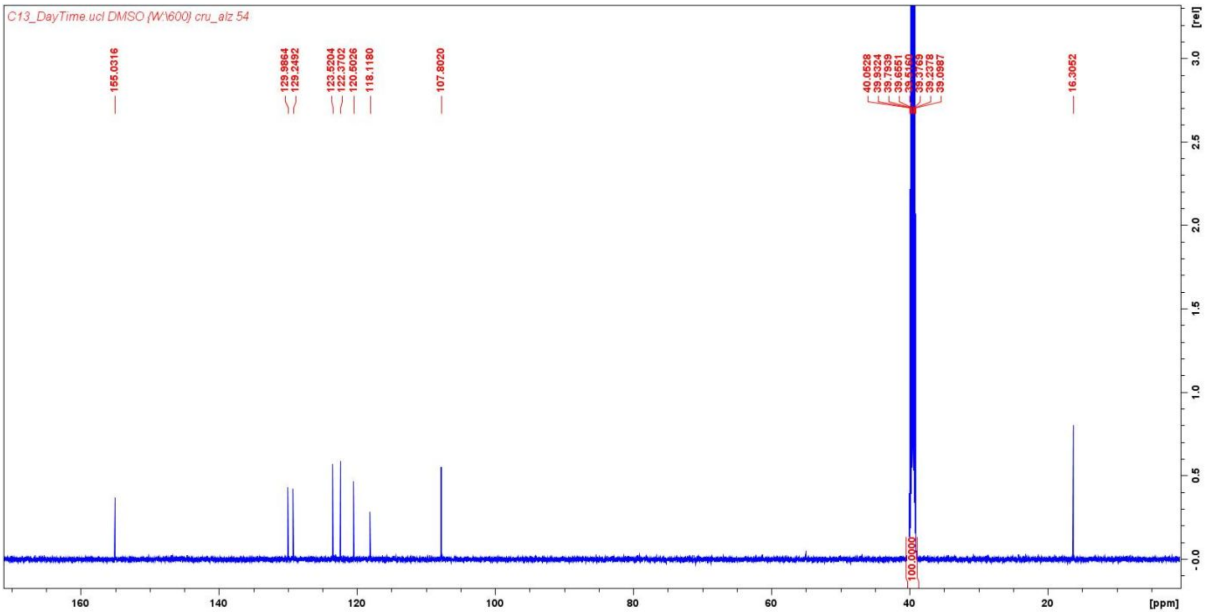

F: LCMS for 15a.

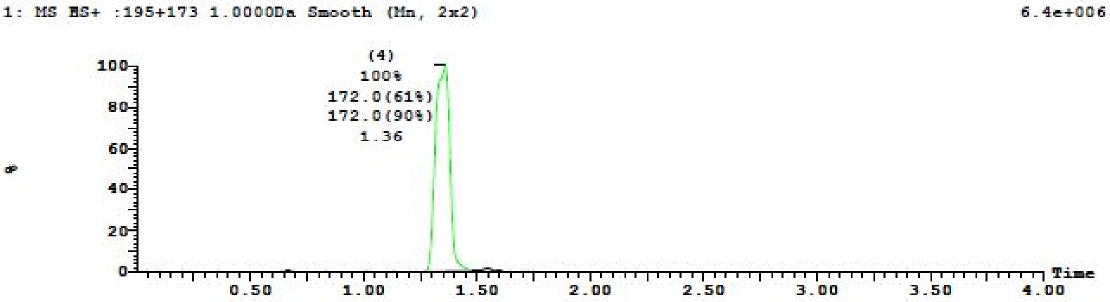

Peak Number	Compound	Time	AreaAbs	Area %Total	Width	Height	Mass Found
4	Found	1.36	5e+005	100.00	0	6e+006	)000, 172.0000

Peak ID	Compound	Time	Mass Found
4	Found	1.36	195,173

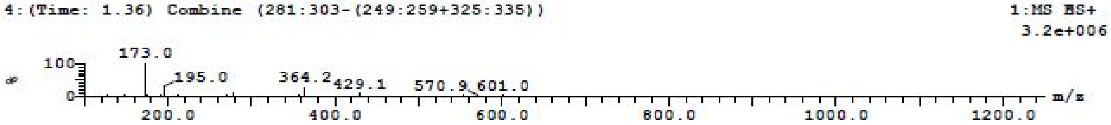

G: ^{1}H NMR for 16a.

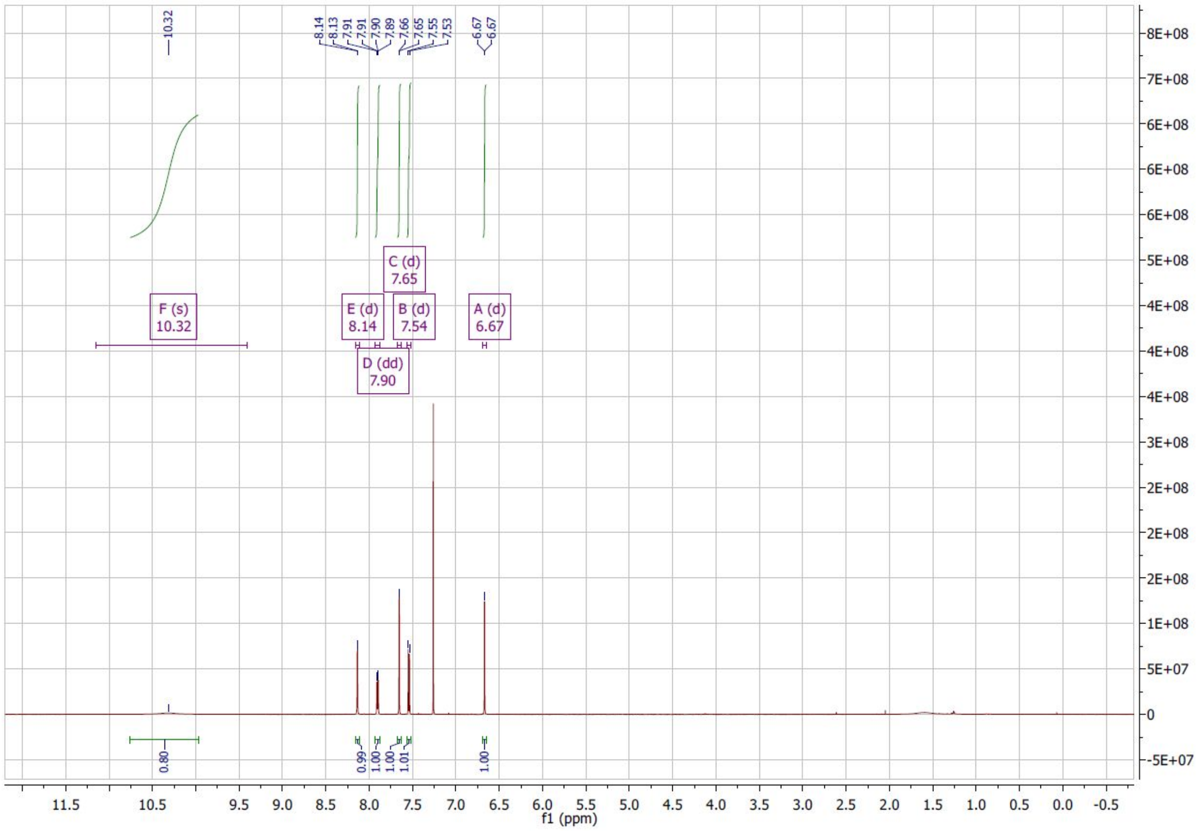

H: ^{13}C NMR for 16a.

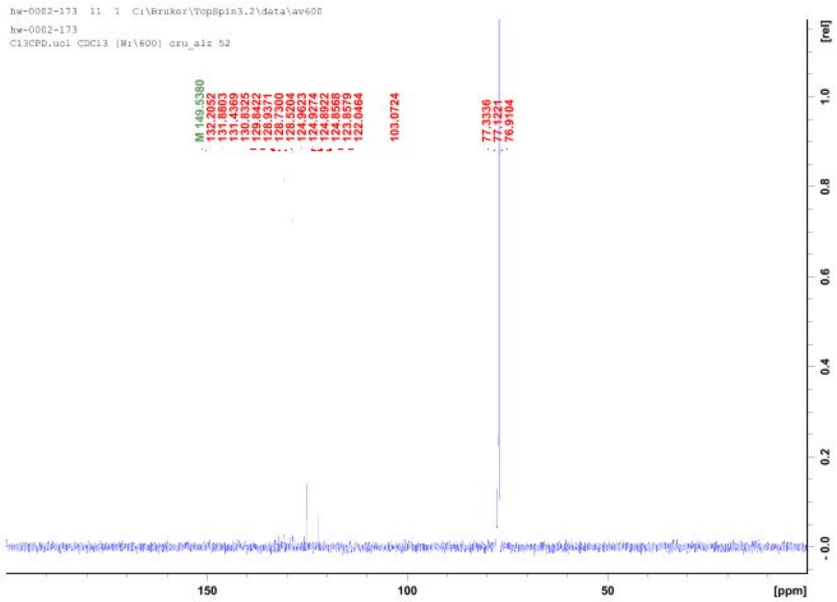

I: LCMS for 16a.

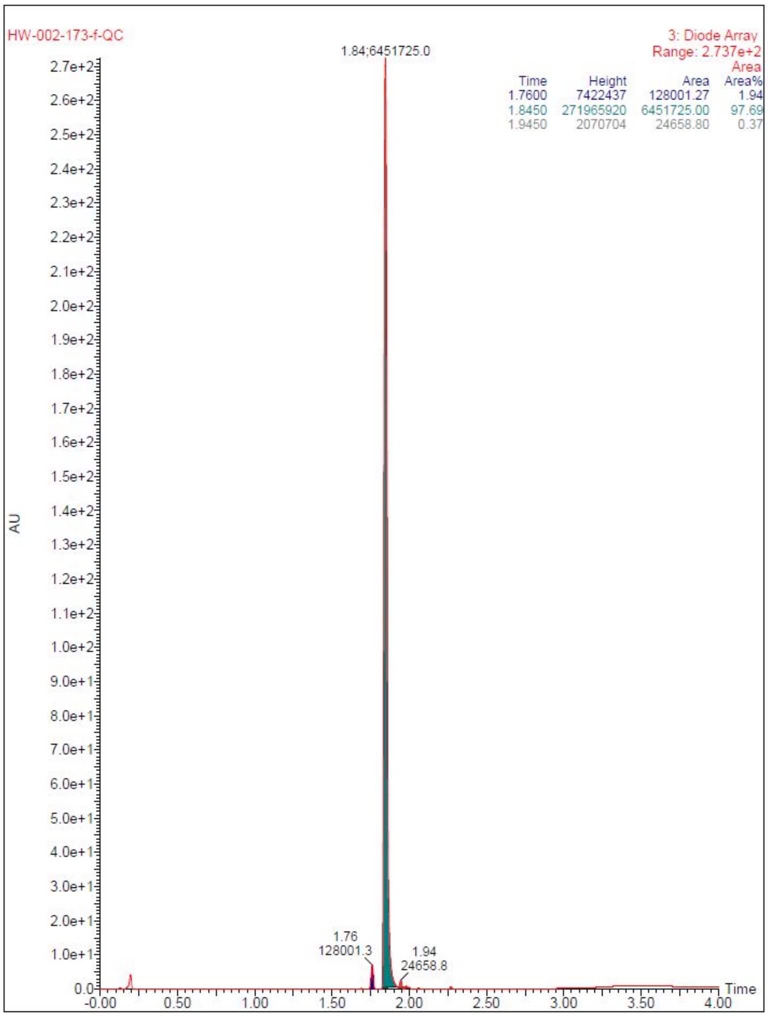

Peak ID	Compound	Time	Mass Found
4		1.79	Not Found

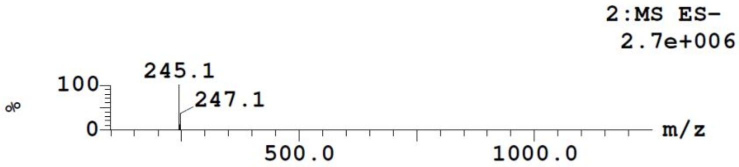

J: ^{1}H NMR for 28a.

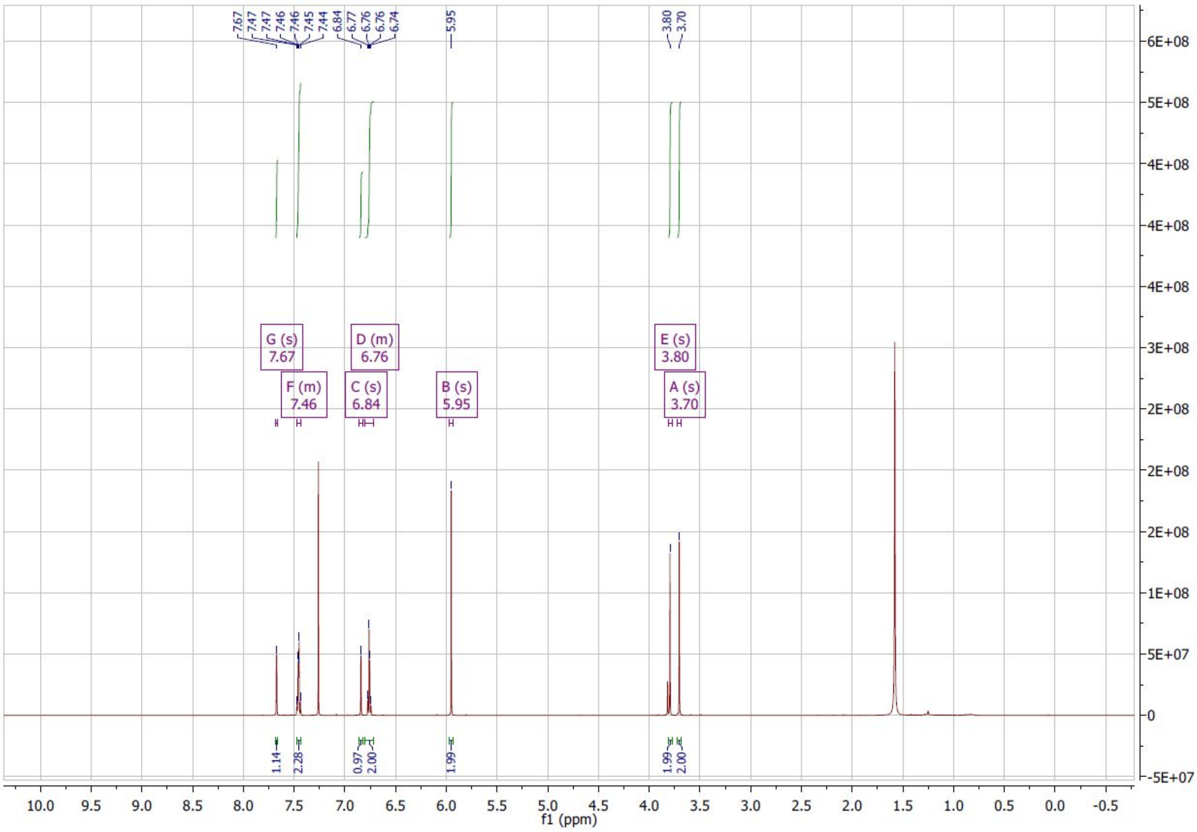

K: ^{13}C NMR for 28a.

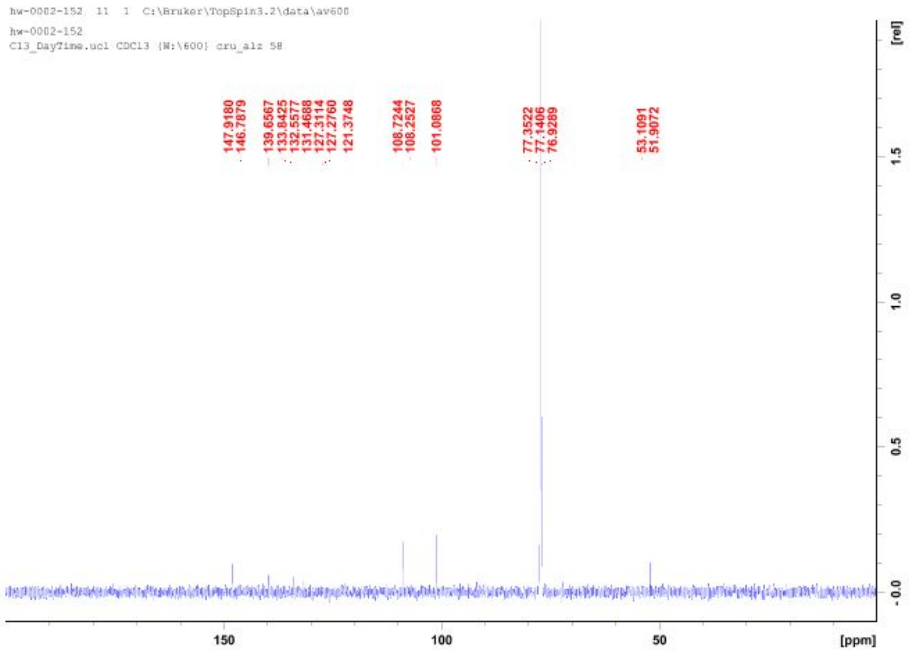

L: LCMS for 28a.

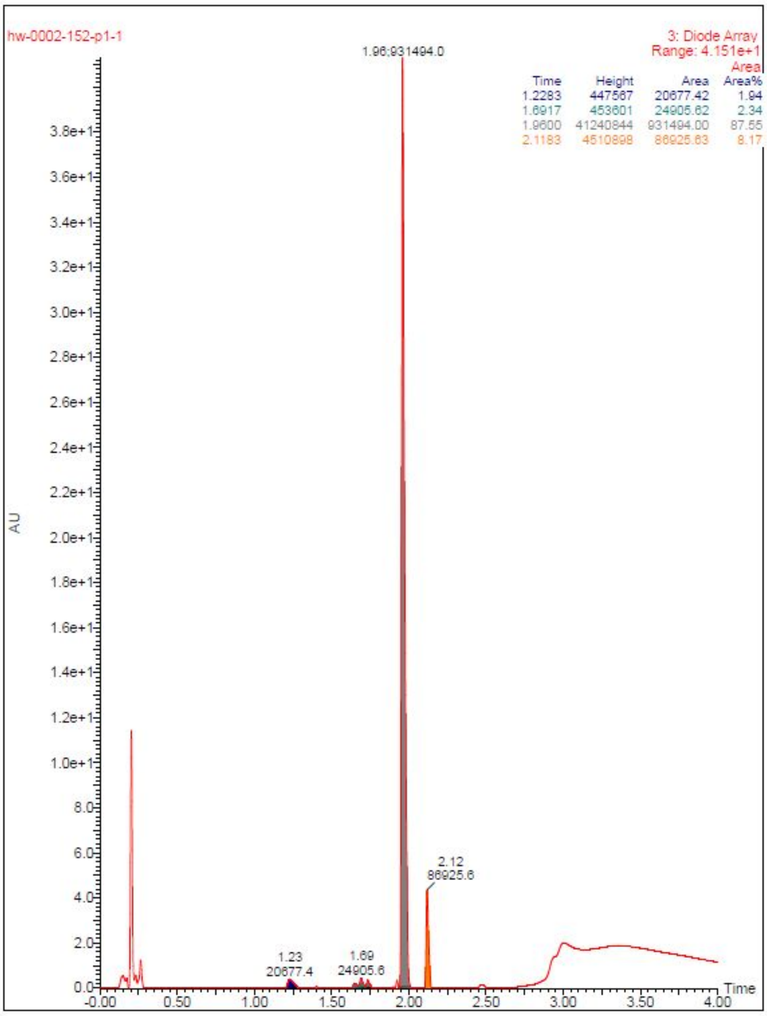

Peak ID	Compound	Time	Mass Found
10		1.97	Not Found

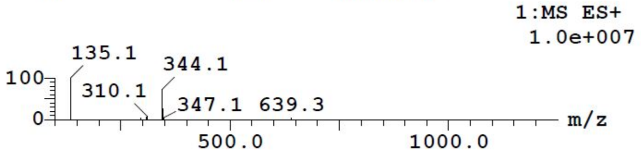

Figure S4. Analytical data for Advanced Lead 7d.

A. UHPLC (C18 CSH; Mobile Phase A: Water (0.1% TFA v/v)); Mobile Phase B: MeCN) for **7d**.

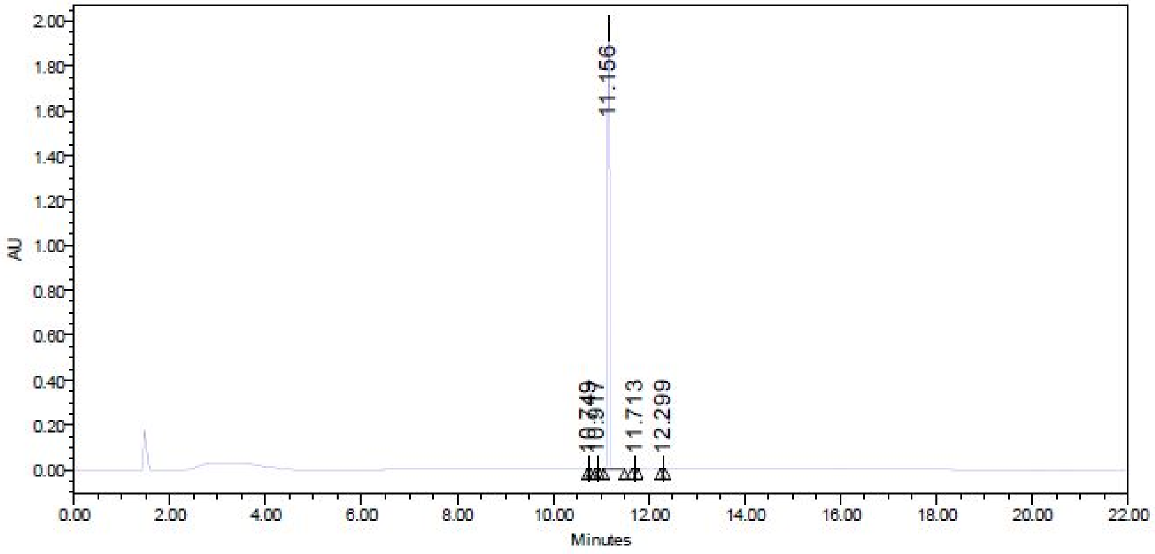

	Retention Time (min)	Area (µV*sec)	% Area	Width (sec)
1	10.75	247	0.00	5.001
2	10.92	895	0.01	6.101
3	11.16	6082021	99.97	26.106
4	11.71	330	0.01	8.352
5	12.30	166	0.00	6.151

Complete spectroscopic and analytical data for **7d** are reported in reference 43.
